# Supplementary material for: Reactions of Trifluorotriacetic Acid Lactone and Hexafluorodehydroacetic Acid with Amines: Synthesis of Trifluoromethylated 4-Pyridones and Aminoenones
Source: Molecules. 2022 Oct 20;27(20):7098. doi: 10.3390/molecules27207098 (PMC9610390; doi:10.3390/molecules27207098)
Supplement: Supplementary file 1 [file molecules-27-07098-s001.zip › molecules-1951436-supplementary.pdf]

Supplementary Materials

Reactions of Trifluorotriacetic Acid Lactone and Hexafluorodehydroacetic Acid with Amines: Synthesis of Trifluoromethylated 4-Pyridones and Aminoenones

Vladislav V. Fedin, Sergey A. Usachev, Dmitrii L. Obydennov and Vyacheslav Y. Sosnovskikh *

Institute of Natural Sciences and Mathematics, Ural Federal University, 51 Lenina Ave., 620000 Ekaterinburg, Russian Federation

* Correspondence: vy.sosnovskikh@urfu.ru; Tel.: +7 (343) 3899597

Table of contents

Figure S1. UV/Vis spectra of compounds **5** S2

Table S1. Characteristic chemical shifts in ^{1}H, ^{13}C, and ^{19}F NMR spectra of compounds **3** S3

Table S2. Characteristic chemical shifts in ^{1}H, ^{13}C, and ^{19}F NMR spectra of compounds **5** S4

Table S3. Characteristic chemical shifts in ^{1}H, ^{13}C, and ^{19}F NMR spectra of *E*- and *Z*-isomers of enaminones **9** S5

^{1}H, ^{19}F, and ^{13}C NMR spectra of compounds **1–5**, **9, 10**, **13**, **14** and **B′** S6-S70

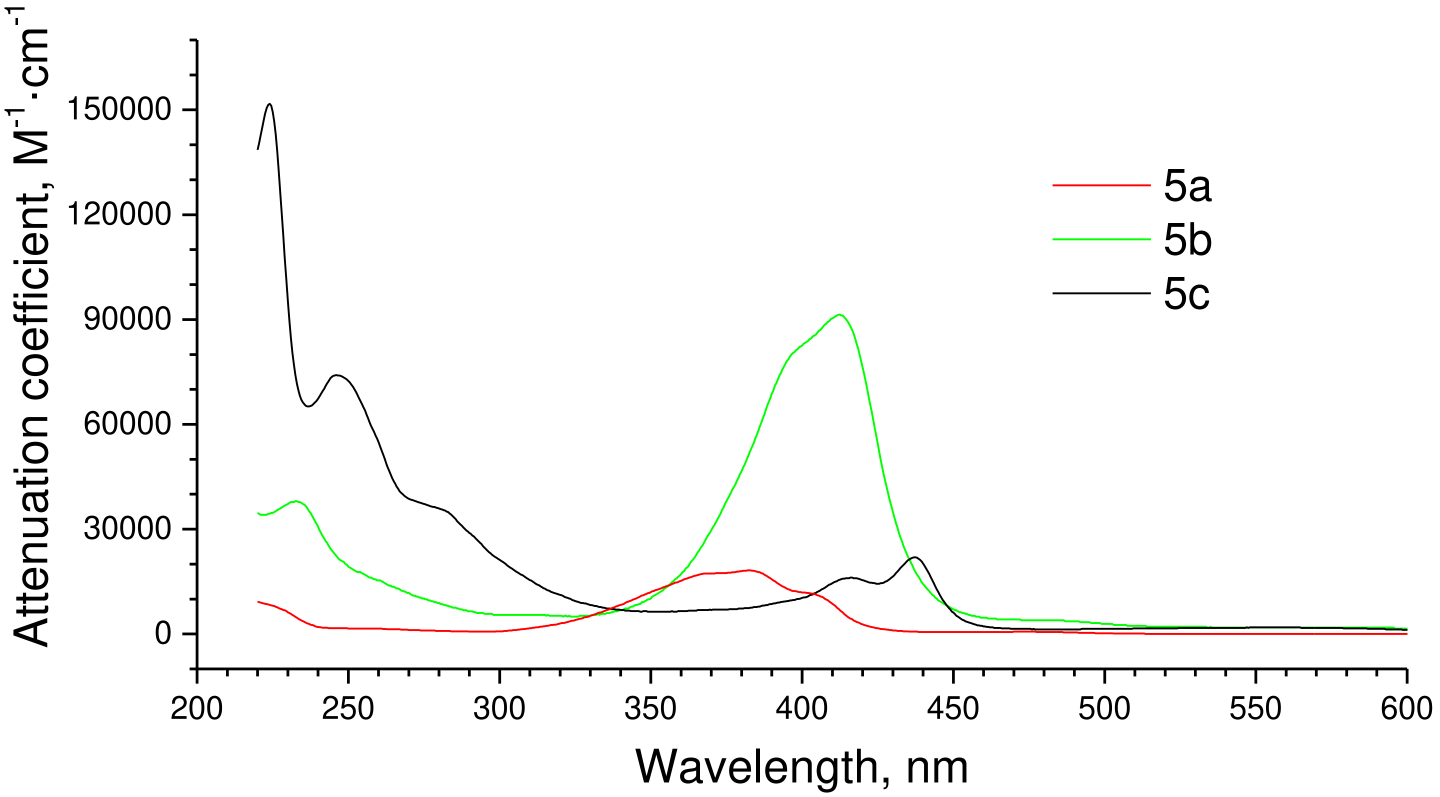

Figure S1. UV/Vis spectra of compounds **5** in EtOH (10^{-5}–10^{-6} M)

Table S1. Characteristic chemical shifts in the ^{1}H, ^{13}C, and ^{19}F NMR spectra of compounds **3** in $CDCl_3$, δ, ppm (J, Hz)

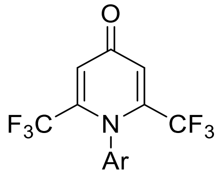

**3**	Ar	$C\boldsymbol{F}_3$	3-C$\boldsymbol{H}$,5-C$\boldsymbol{H}$	$\boldsymbol{C}F_3$	2-$\boldsymbol{C}CF_3$,6-$\boldsymbol{C}CF_3$	3-$\boldsymbol{C}$H,5-$\boldsymbol{C}$H	4-$\boldsymbol{C}$O
**a**	Ph	100.7	6.97	119.4 (275.7)	141.2 (33.3)	119.4 (4.7)	177.4
**b**	4-$MeOC_6H_4$	100.5	6.95				
**c**	3,4-$F_2C_6H_3$	100.7	6.96	119.3 (275.7)	140.9 (33.3)	119.4 (4.7)	177.1
**d**	4-BrC_6H_4	100.7	6.97	119.3 (275.8)	141.0 (32.8)	119.3 (4.5)	177.3
**e**	2,5-$F_2C_6H_3$	98.5	6.99	119.3 (275.5)	140.8 (34.1)	119.5 (4.6)	177.2
**f**	3-AcC_6H_4	100.8	6.99	119.2 (276.1)	141.6 (unresolved)	119.0 (unresolved)	177.2
**g**	3-$F_3CC_6H_4$	98.7	6.99	119.3 (275.5)	140.9 (33.4)	119.4 (4.7)	177.2
**h**	2-MeC_6H_4	99.0	7.01	119.3 (275.8)	141.3 (32.2)	119.6 (4.2)	177.9
**i**	2-ClC_6H_4	98.7	7.02	119.3 (275.8)	140.9 (33.9)	119.6 (4.5)	177.6
**j**	2,5-$Me_2C_6H_3$	99.0	7.01				
**k**	4-$O_2NC_6H_4$	101.0	7.01				

Table S2. Characteristic chemical shifts in the ^{1}H, ^{13}C, and ^{19}F NMR spectra of compounds **5** in DMSO-*d*$_6$, δ, ppm (*J*, Hz)

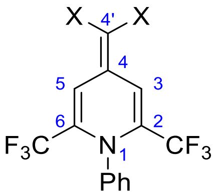

**5**	Structure	C***F***$_3$	***H***-3,5	***C***F$_3$	***C***-2, ***C***-6	***C***-3, ***C***-5	***C***-4	***C***-4’	2C Ph	2C Ph	C Ph	C Ph
**a**	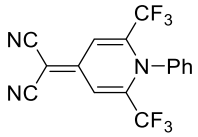	102.5	7.26	118.7 (275.9)	138.2 (33.0)	112.7 (5.3)	154.2	57.1	130.0	130.4	132.1	135.6
**b**	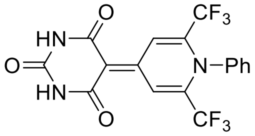	103.2	9.77	119.8 (275.5)	138.3 (32.3)	116.8 (5.7)	153.0	95.0	128.7	130.1	131.7	134.6
**c**	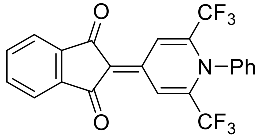	102.8	9.10	119.2 (275.2)	138.9 (32.7)	113.4 (5.7)	146.0	107.2	128.5	130.0	131.6	135.1

Table S3. Characteristic chemical shifts in the ^{1}H, ^{13}C, and ^{19}F NMR spectra of *E*- and *Z*-isomers of enaminones **9** used for analysis of equilibrium mixtures, δ, ppm (*J*, Hz)

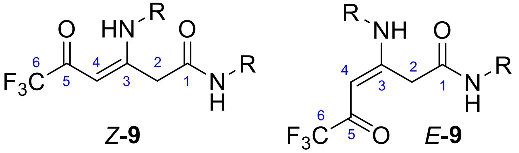

R	Solvent	Enaminone	C*F*$_3$	2-C*H*$_2$	4-C*H*	1-N*H*	3-N*H*	1-*C*O	2-*C*H$_2$	*C*-3	4-*C*H	5-*C*O	*C*F$_3$
Ph	DMSO-d$_6$	***Z*-9a**	87.1	3.65	5.79	10.08	12.56						
		***E*-9a**	86.6	4.06	5.59	10.23 and 10.31							
Ph	CDCl$_3$	***Z*-9a**	85.1	3.49	5.72	7.77	12.58	163.4	41.5	164.0	91.3	177.8 (33.7)	117.2 (288.3)
		***E*-9a**	84.8	4.04	5.82	8.71	9.70	162.5	42.3	166.3	88.7	177.6 (32.1)	117.6 (290.2)
4-BrC$_6$H$_4$	CDCl$_3$	***Z*-9b**	85.0	3.41	5.70	7.43	12.42						
		***E*-9b**	84.8	3.98	5.78	8.32	9.62						
4-MeOC$_6$H$_4$	CDCl$_3$	***Z*-9c**	85.1	3.38	5.68	7.12	12.39	164.0 or 164.2	41.4	164.0 or 164.2	90.9	177.5 (33.6)	117.3 (288.6)
		***E*-9c**	84.9	4.00	5.68	8.74	9.63	163.2	42.0	165.9	88.25	177.9 (31.3)	117.7 (290.2)
Bn	DMSO-d$_6$	***Z*-9d**		3.55	5.50	8.76	11.29						
		***E*-9d**		3.84	5.27	8.45	9.08						
Bn	CDCl$_3$	***Z*-9d**	85.1	3.31	5.44	6.04	11.32	164.9	41.1	165.3	90.2	177.0 (33.5)	117.3 (288.4)
		***E*-9d**	84.9	3.79	5.41	7.90	7.90	163.5	40.5	168.0	86.3	176.9 (32.1)	117.7 (290.5)
Bu	CDCl$_3$	***Z*-9e**	85.0	3.27	5.36	5.65	11.08						
		***E*-9e**	84.9	3.75	5.32	7.10	7.36						

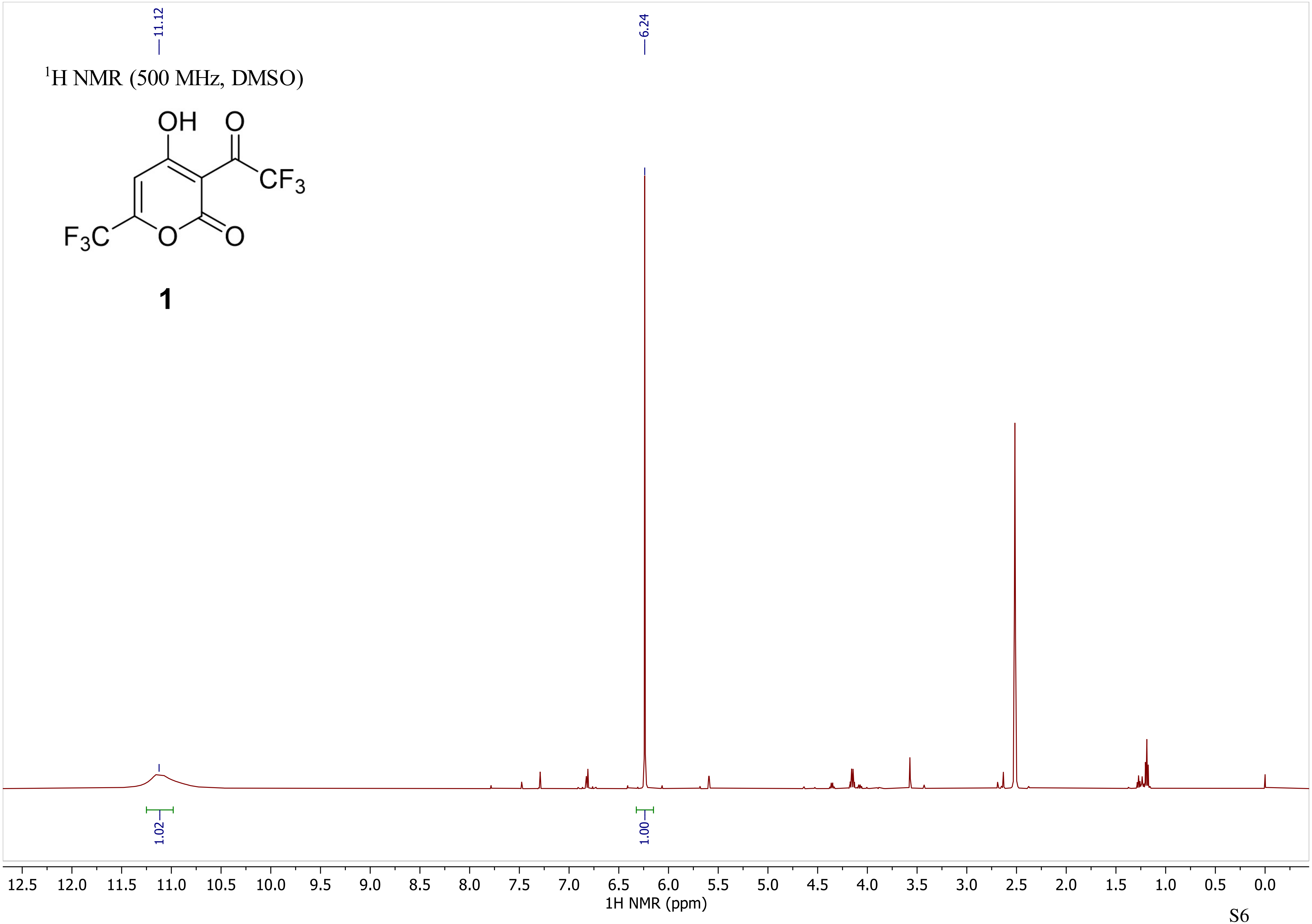

1H NMR (500 MHz, DMSO)
OH
O
CF3
F3C
O
O
1
11.12
6.24
1.02
1.00
12.5 12.0 11.5 11.0 10.5 10.0 9.5 9.0 8.5 8.0 7.5 7.0 6.5 6.0 5.5 5.0 4.5 4.0 3.5 3.0 2.5 2.0 1.5 1.0 0.5 0.0
1H NMR (ppm)

^{19}F NMR (471 MHz, DMSO)

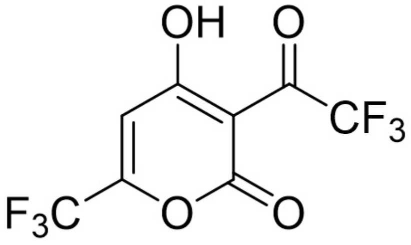

1

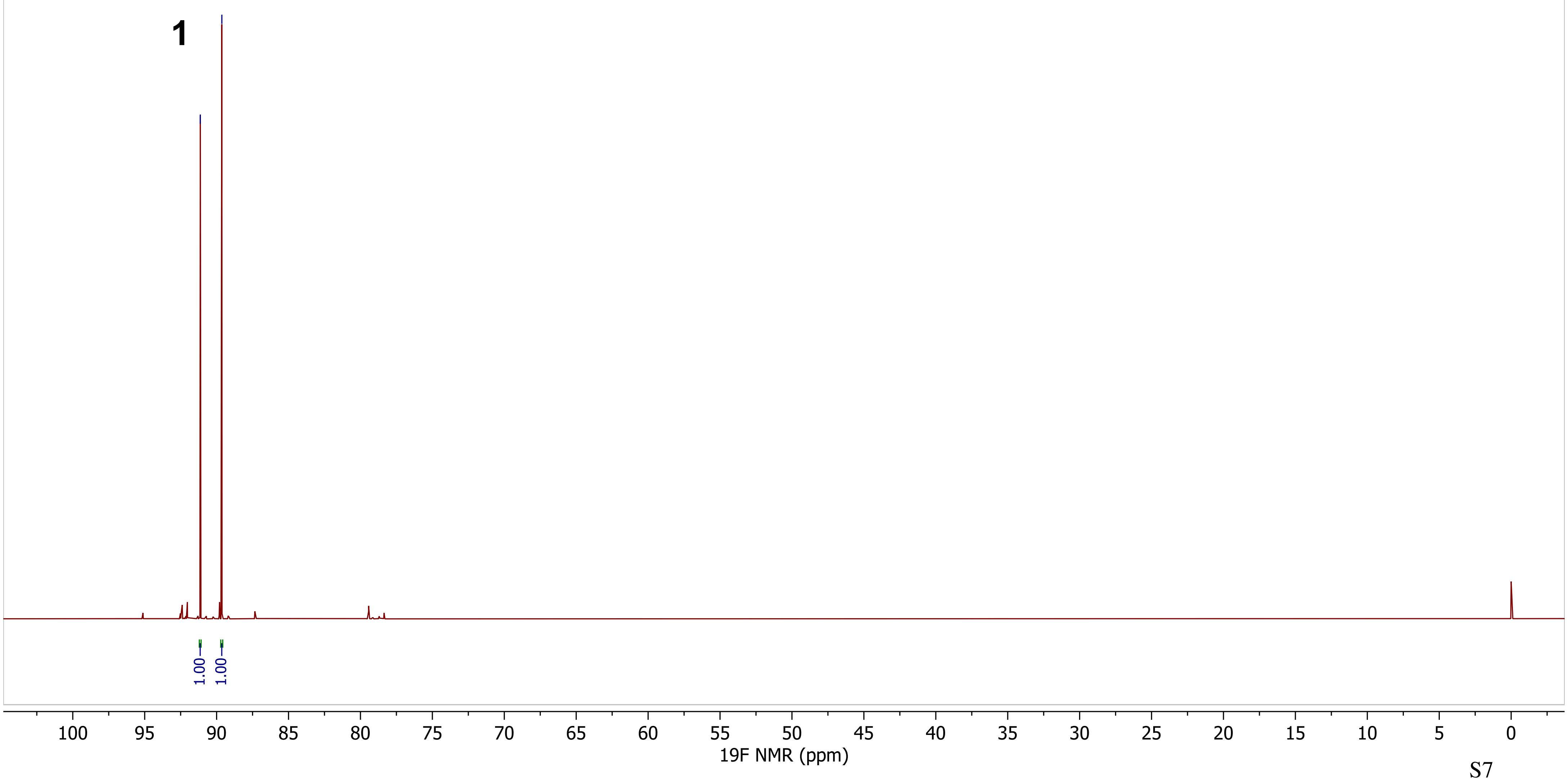

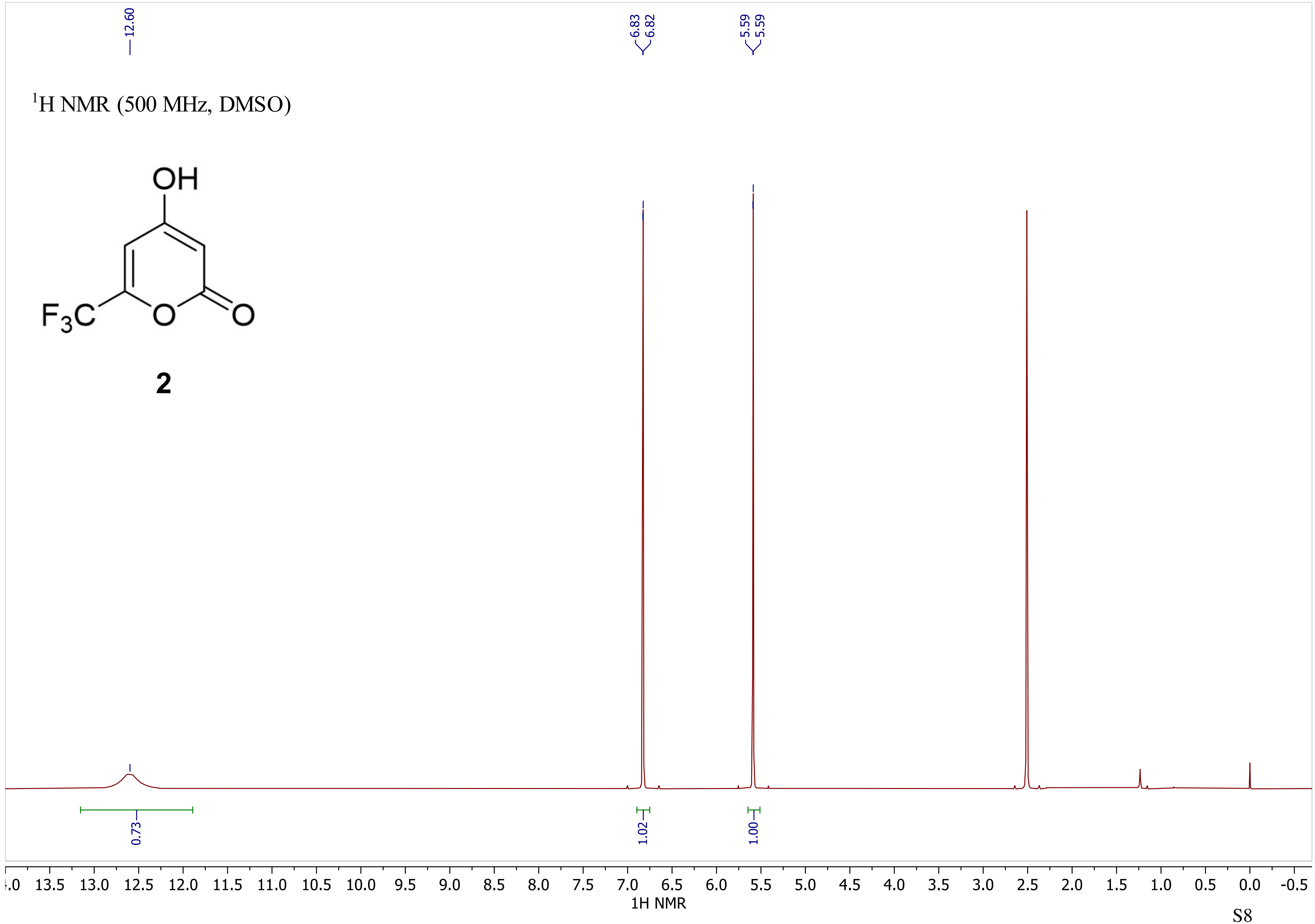

^{1}H NMR (500 MHz, DMSO)
OH
F3C
O
O
2
12.60
6.83
6.82
5.59
5.59
0.73
1.02
1.00
14.0 13.5 13.0 12.5 12.0 11.5 11.0 10.5 10.0 9.5 9.0 8.5 8.0 7.5 7.0 6.5 6.0 5.5 5.0 4.5 4.0 3.5 3.0 2.5 2.0 1.5 1.0 0.5 0.0 -0.5
1H NMR

^{19}F NMR (471 MHz, DMSO) δ 92.02.

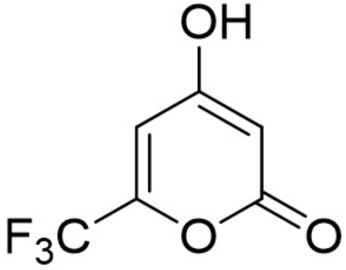

2

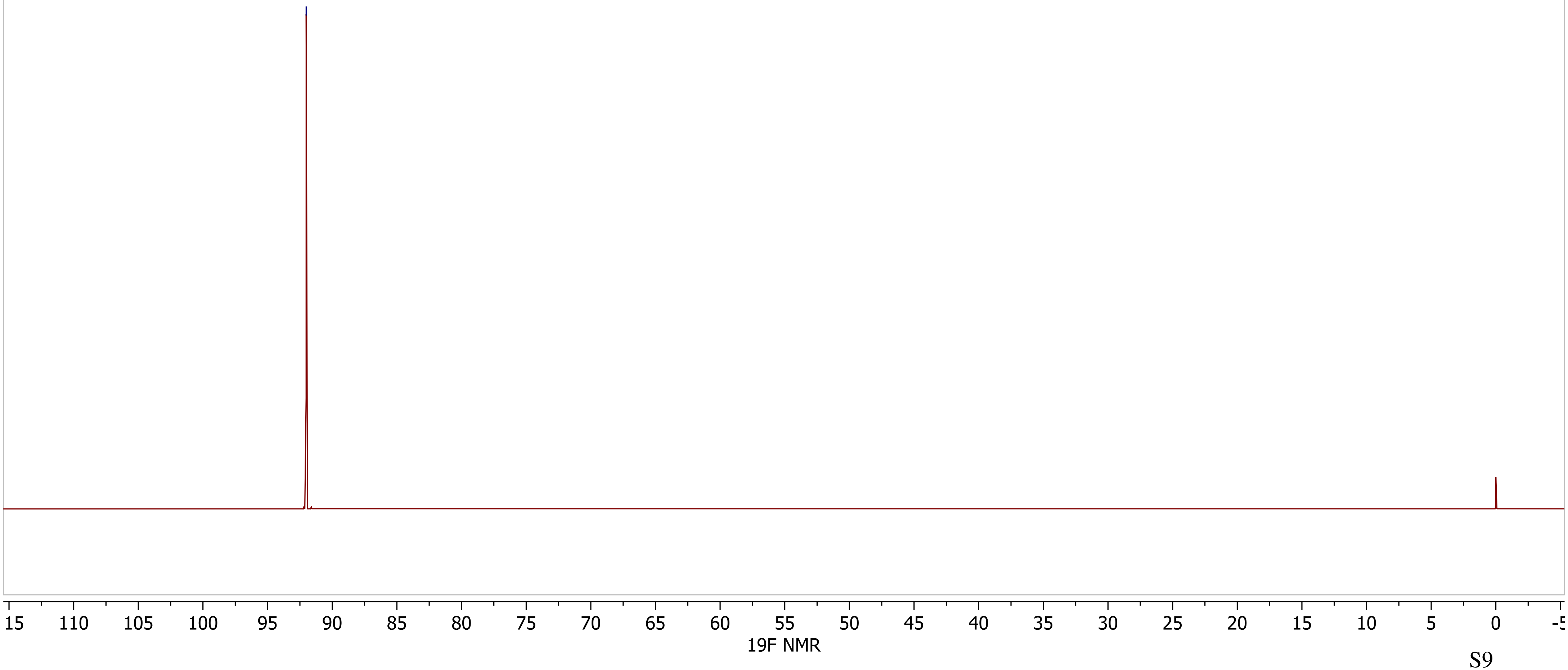

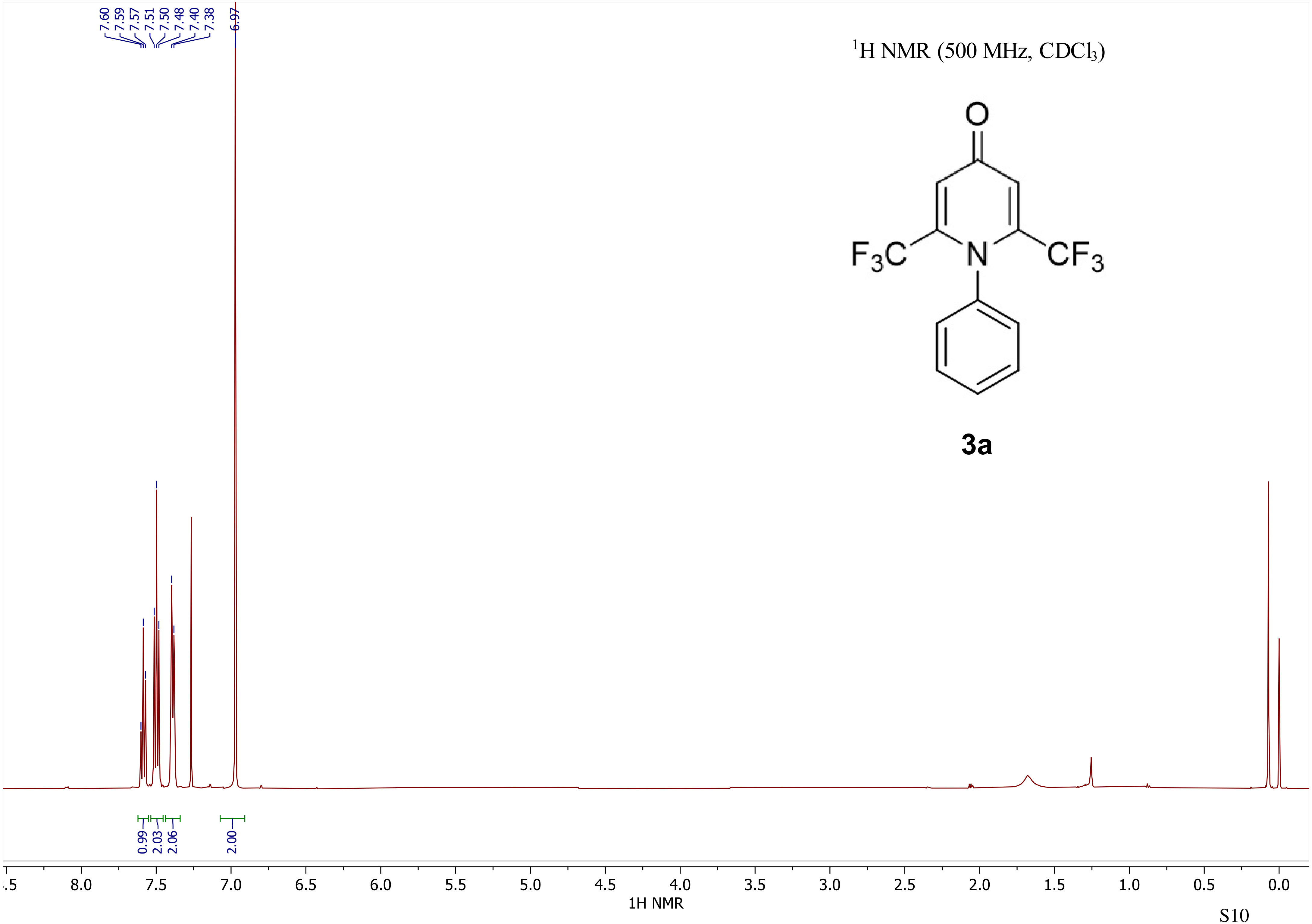

7.60
7.59
7.57
7.51
7.50
7.48
7.40
7.38
6.97
1H NMR (500 MHz, CDCl3)
O
F3C
N
CF3
3a
0.99
2.03
2.06
2.00
8.0
7.5
7.0
6.5
6.0
5.5
5.0
4.5
4.0
3.5
3.0
2.5
2.0
1.5
1.0
0.5
0.0
1H NMR

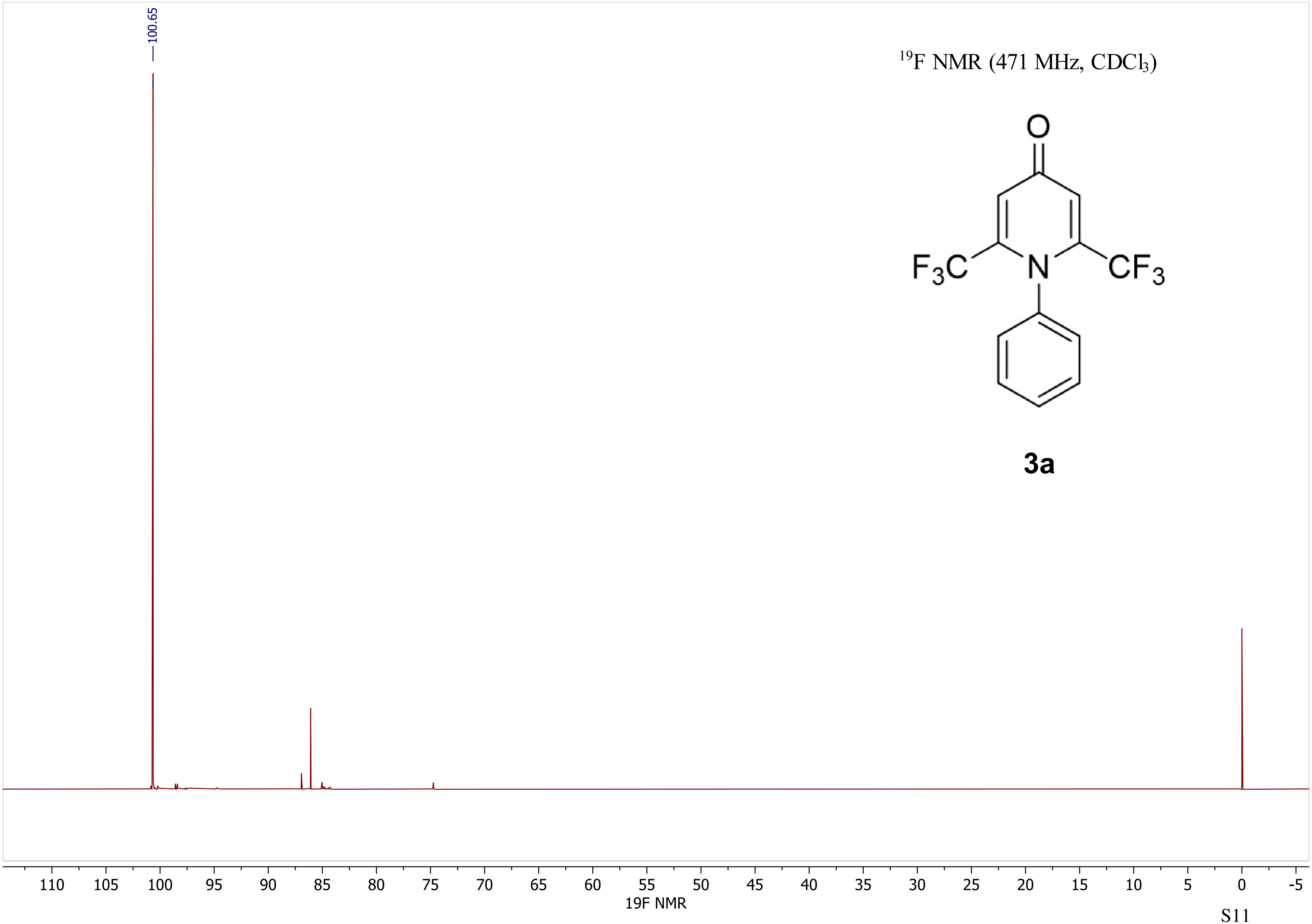

100.65
^{19}F NMR (471 MHz, $CDCl_3$)
O
F_3C
N
CF_3
3a
110 105 100 95 90 85 80 75 70 65 60 55 50 45 40 35 30 25 20 15 10 5 0 -5
19F NMR

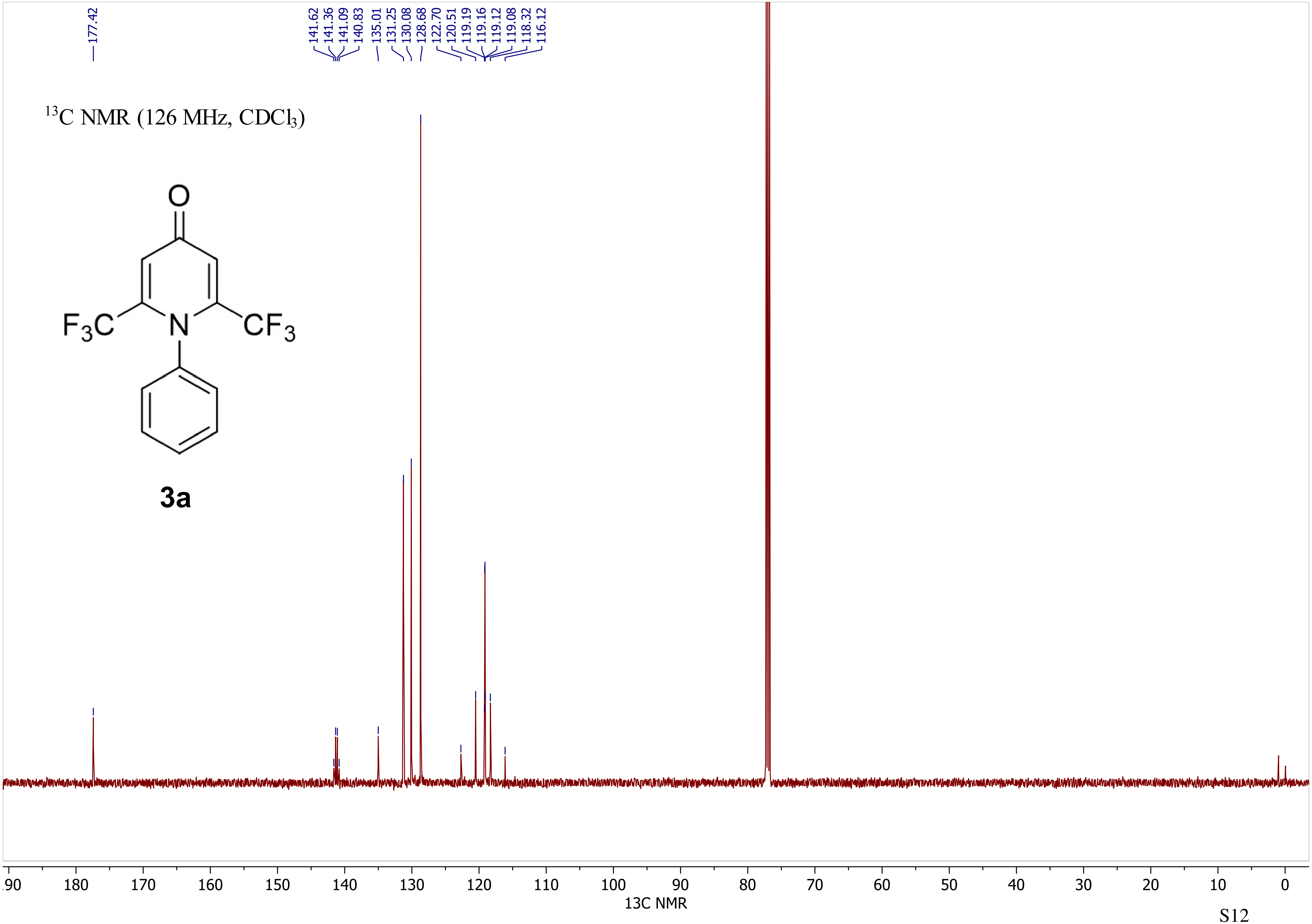
177.42
141.62
141.36
141.09
140.83
135.01
131.25
130.08
128.68
122.70
120.51
119.19
119.16
119.12
119.08
118.32
116.12
^{13}C NMR (126 MHz, $CDCl_3$)
O
F_3C
N
CF_3
3a
190 180 170 160 150 140 130 120 110 100 90 80 70 60 50 40 30 20 10 0
13C NMR

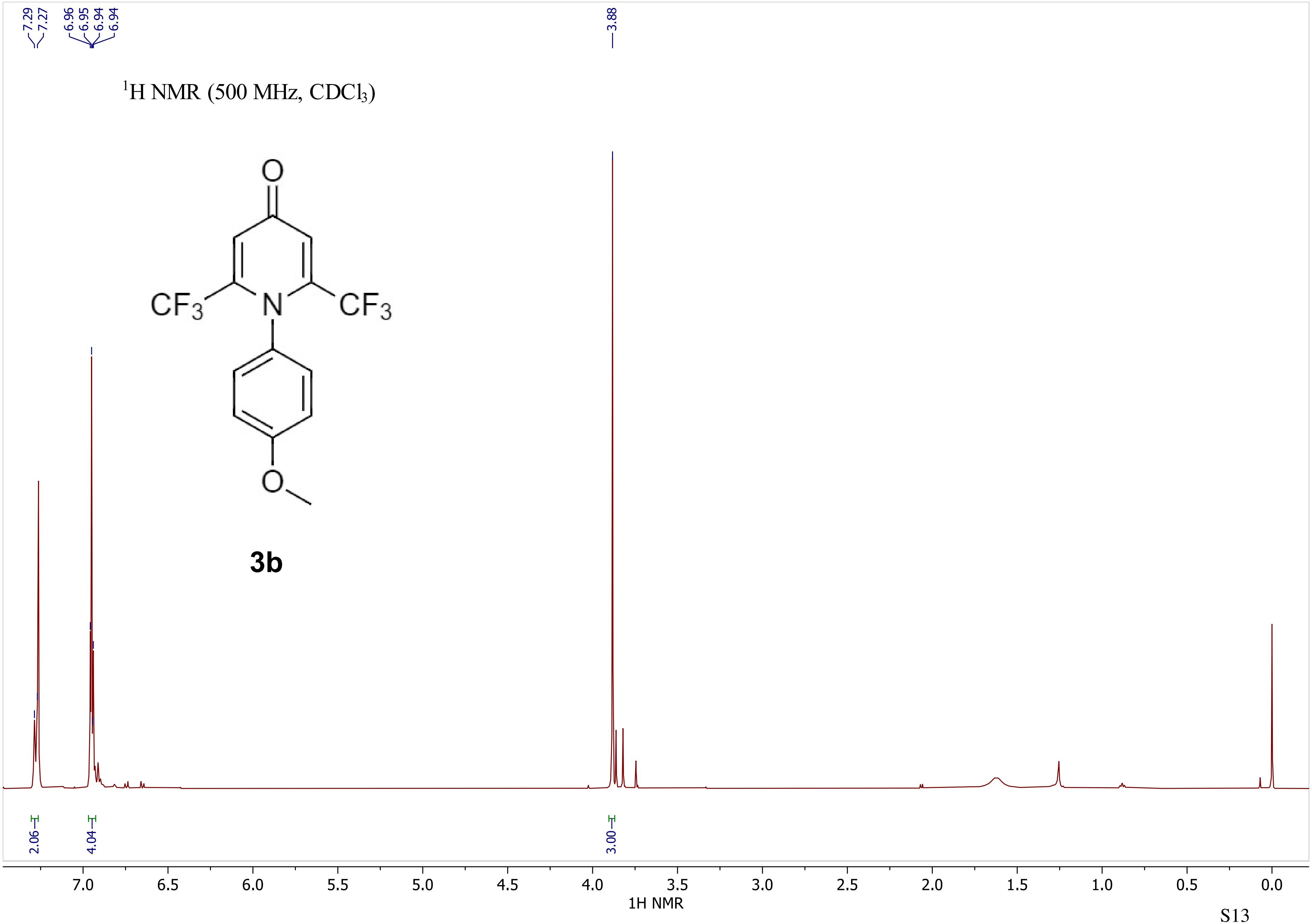

7.29
7.27
6.96
6.95
6.94
6.94
3.88
^{1}H NMR (500 MHz, $CDCl_3$)
O
N
CF_3
CF_3
O
3b
2.06
4.04
3.00
7.0
6.5
6.0
5.5
5.0
4.5
4.0
3.5
3.0
2.5
2.0
1.5
1.0
0.5
0.0
1H NMR

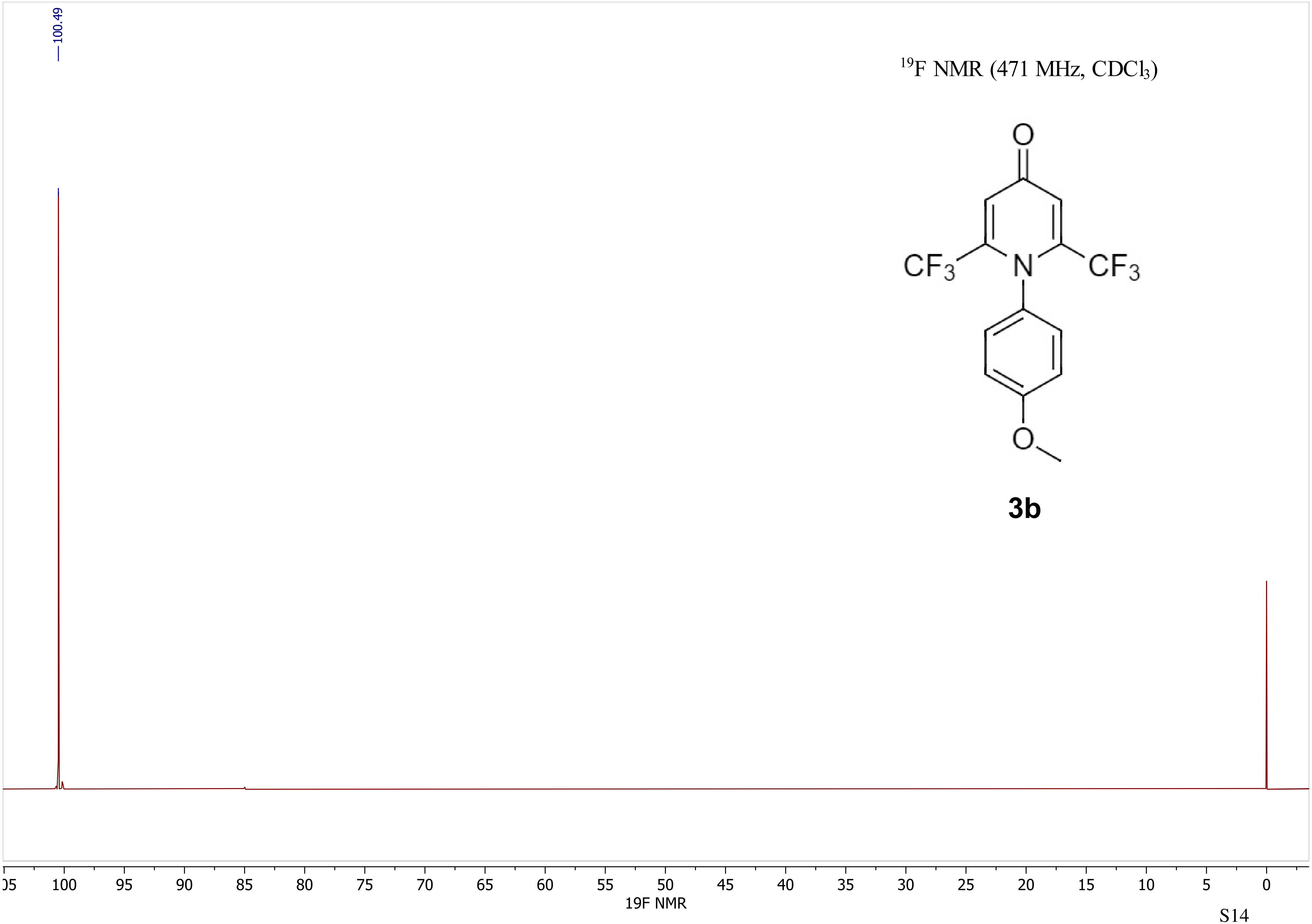

100.49
19F NMR (471 MHz, CDCl3)
O
CF3
N
CF3
O
3b
05
100
95
90
85
80
75
70
65
60
55
50
45
40
35
30
25
20
15
10
5
0
19F NMR

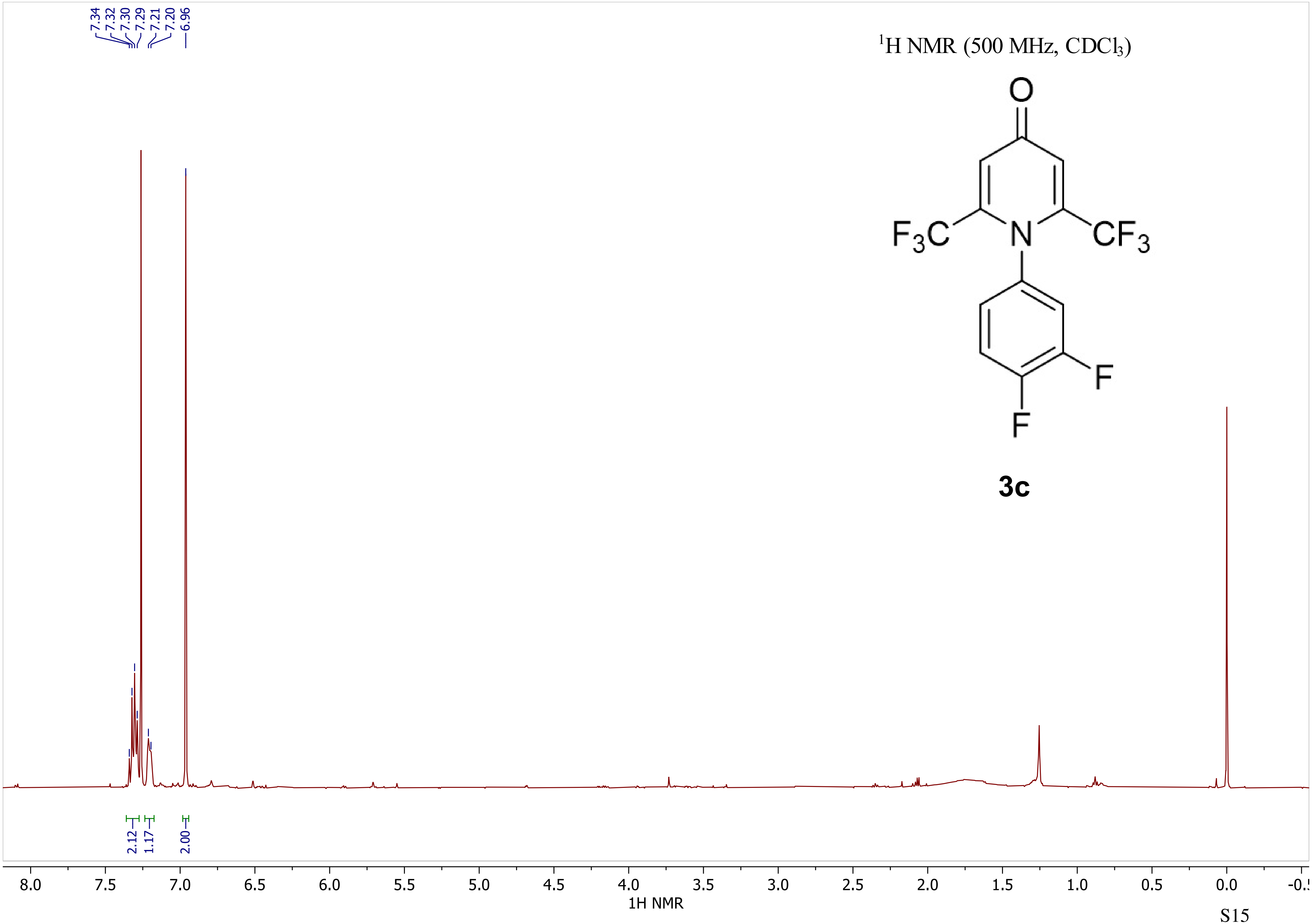
1H NMR (500 MHz, CDCl3)
3c
7.34
7.32
7.30
7.29
7.21
7.20
6.96
2.12
1.17
2.00
8.0
7.5
7.0
6.5
6.0
5.5
5.0
4.5
4.0
3.5
3.0
2.5
2.0
1.5
1.0
0.5
0.0
1H NMR

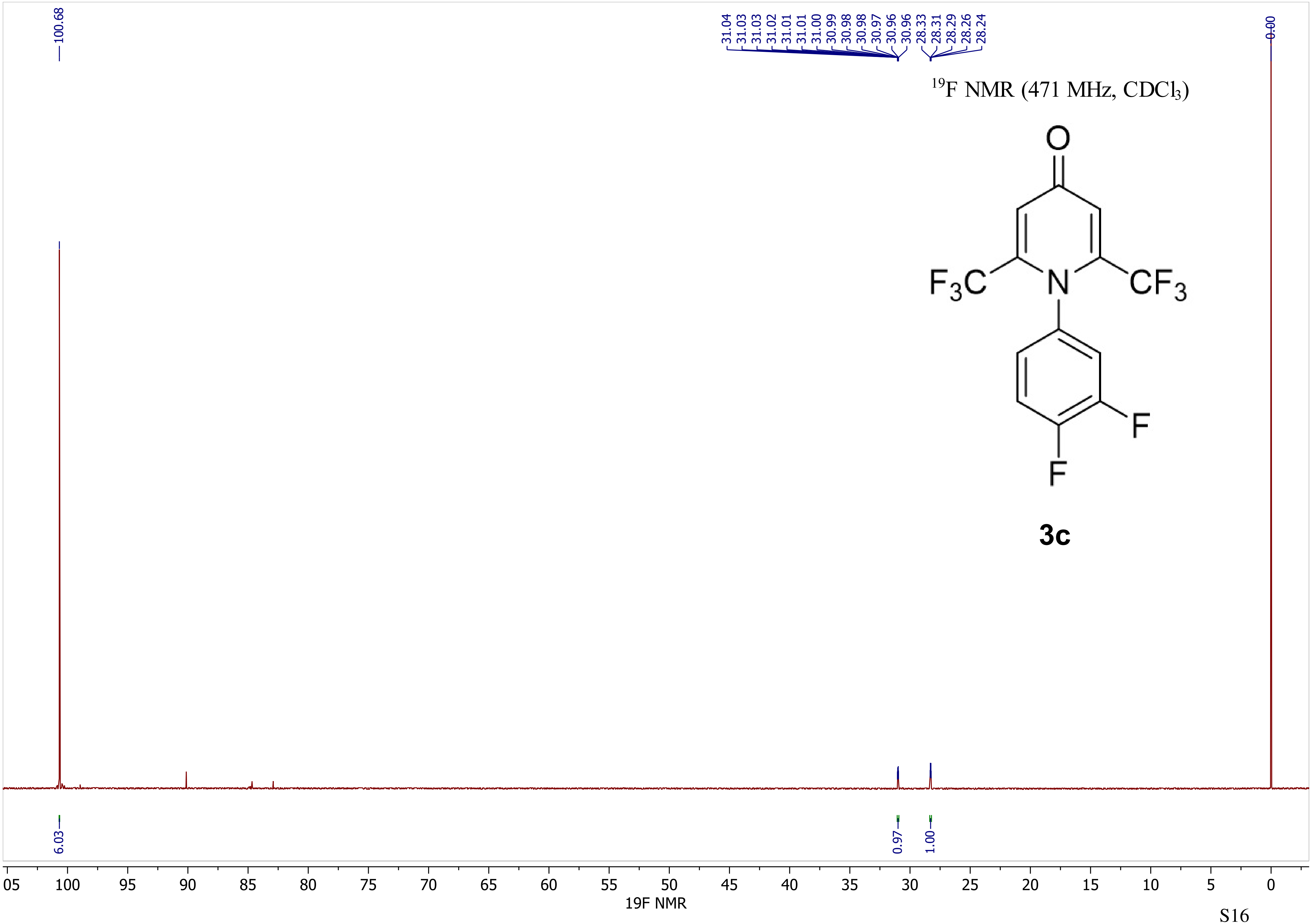

100.68
31.04
31.03
31.03
31.02
31.01
31.01
31.00
30.99
30.98
30.98
30.97
30.96
30.96
28.33
28.31
28.29
28.26
28.24
0.00
19F NMR (471 MHz, CDCl3)
O
N
F3C
CF3
F
F
3c
6.03
0.97
1.00
105
100
95
90
85
80
75
70
65
60
55
50
45
40
35
30
25
20
15
10
5
0
19F NMR

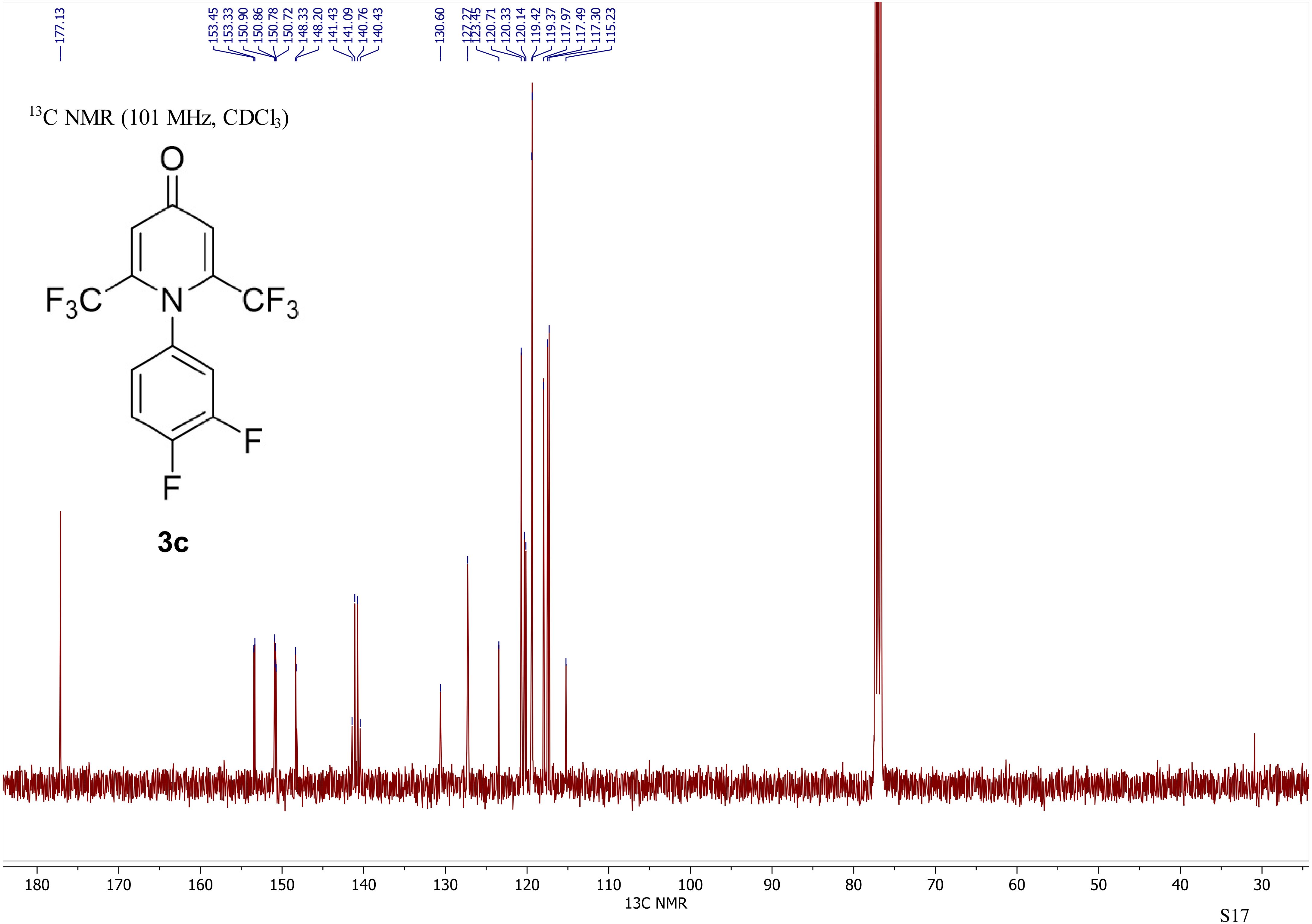

177.13
153.45
153.33
150.90
150.86
150.78
150.72
148.33
148.20
141.43
141.09
140.76
140.43
130.60
127.27
123.45
120.71
120.33
120.14
119.42
119.37
117.97
117.49
117.30
115.23
13C NMR (101 MHz, CDCl3)
O
N
F3C
CF3
F
F
3c
180 170 160 150 140 130 120 110 100 90 80 70 60 50 40 30
13C NMR

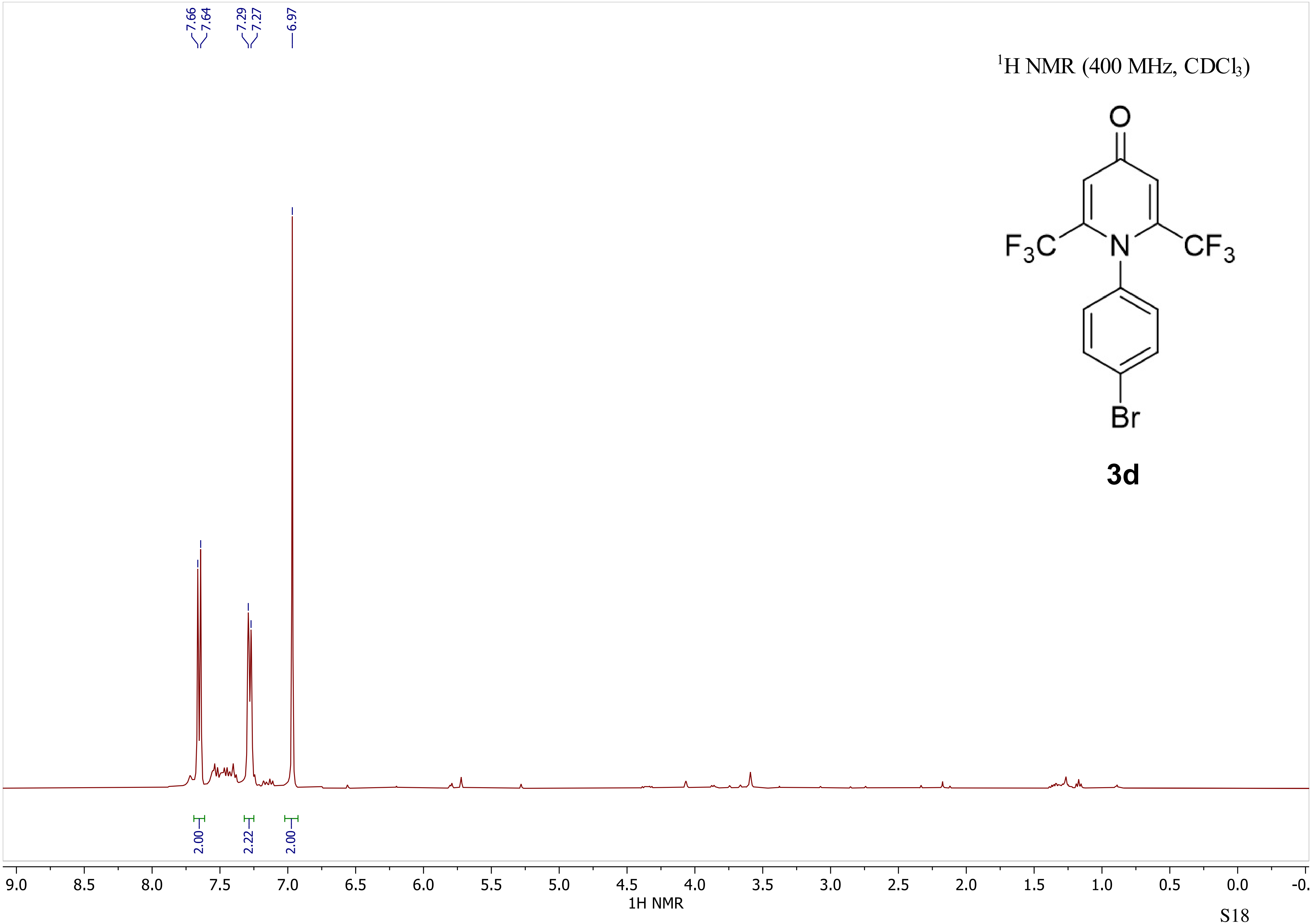

7.66
7.64
7.29
7.27
6.97
¹H NMR (400 MHz, CDCl3)
O
F3C
N
CF3
Br
3d
2.00
2.22
2.00
9.0
8.5
8.0
7.5
7.0
6.5
6.0
5.5
5.0
4.5
4.0
3.5
3.0
2.5
2.0
1.5
1.0
0.5
0.0
-0.
1H NMR

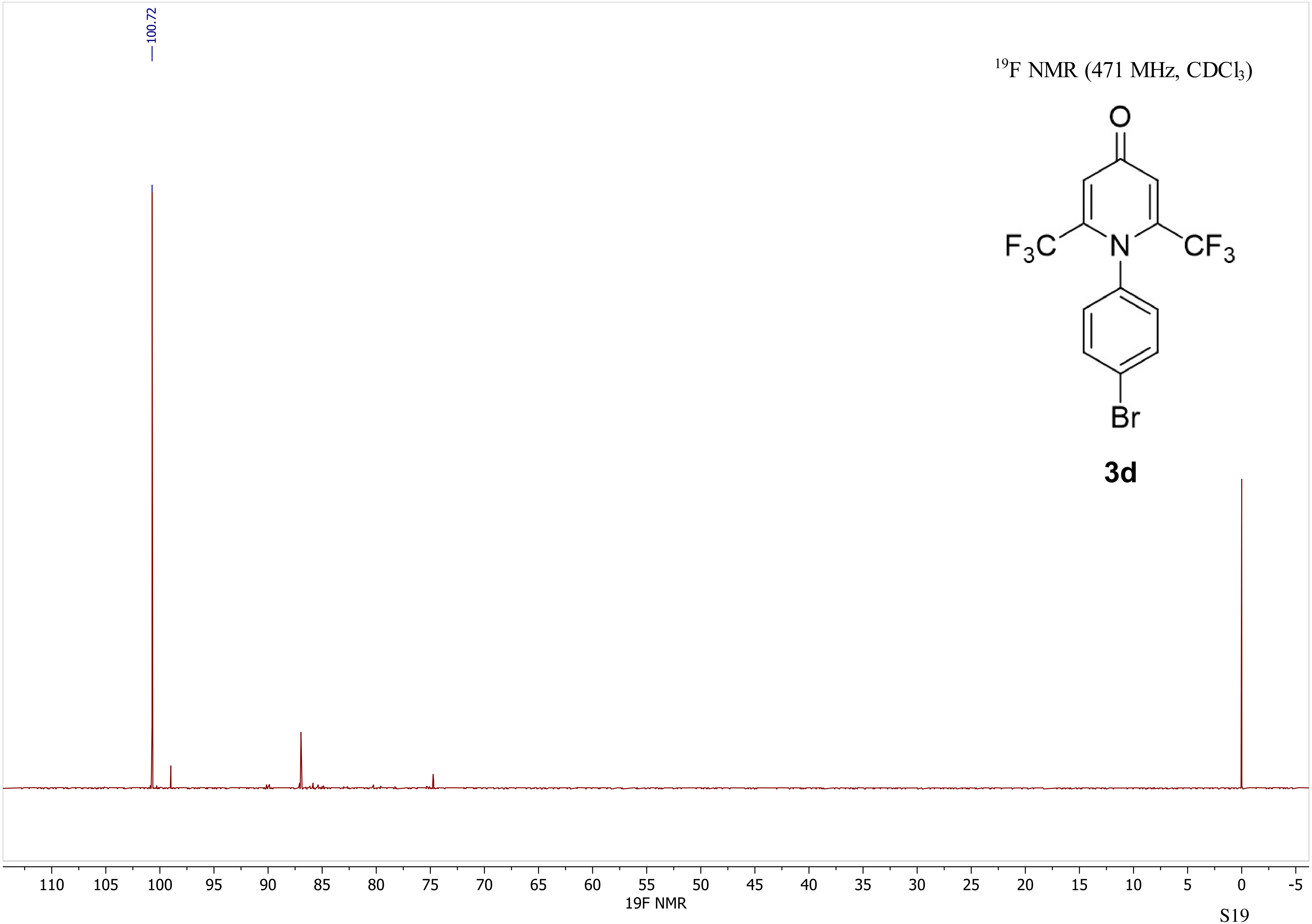
100.72
^{19}F NMR (471 MHz, $CDCl_3$)
O
F_3C
N
CF_3
Br
3d
110
105
100
95
90
85
80
75
70
65
60
55
50
45
40
35
30
25
20
15
10
5
0
-5
19F NMR

^{13}C NMR (101 MHz, $CDCl_3$)

3d

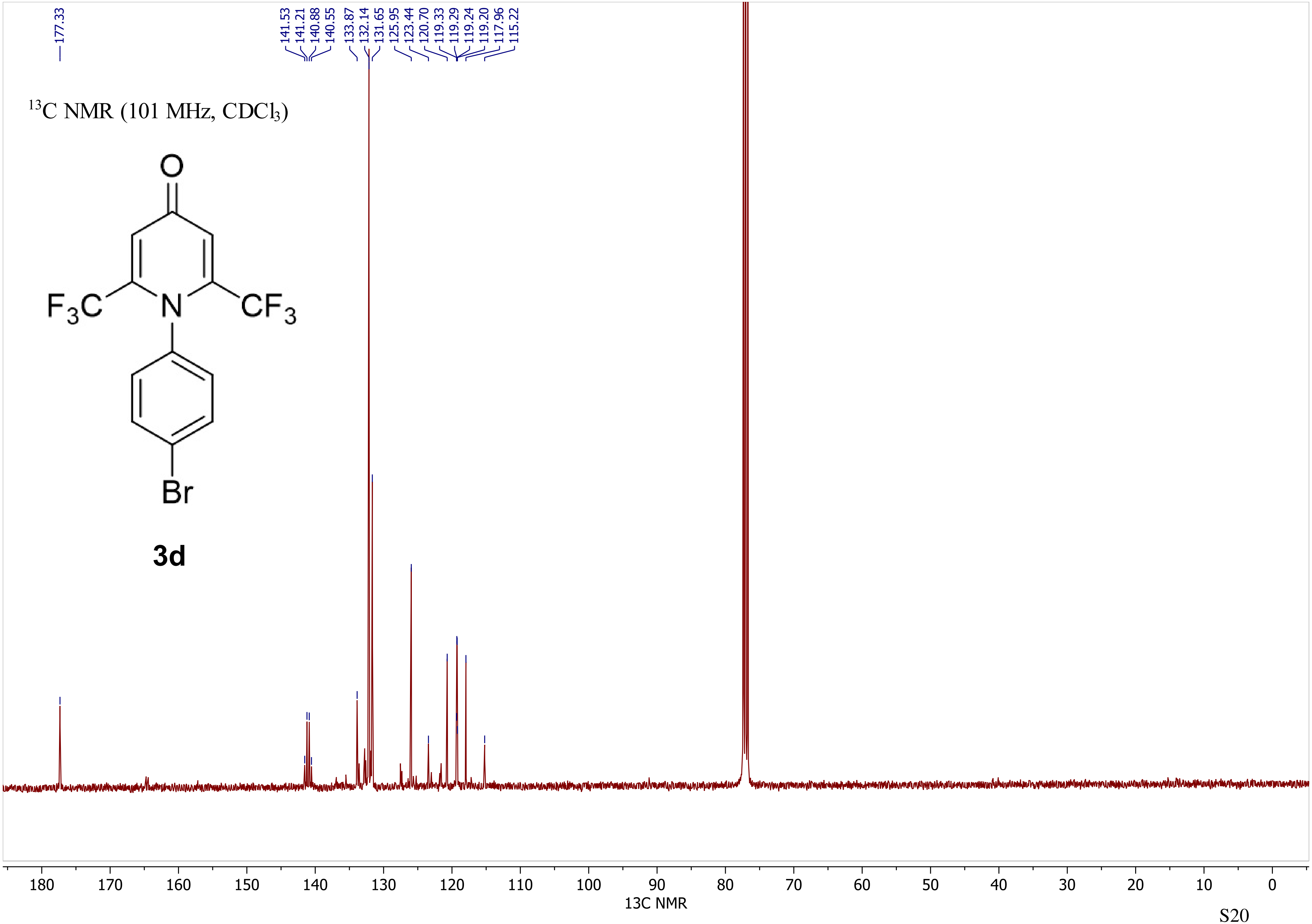

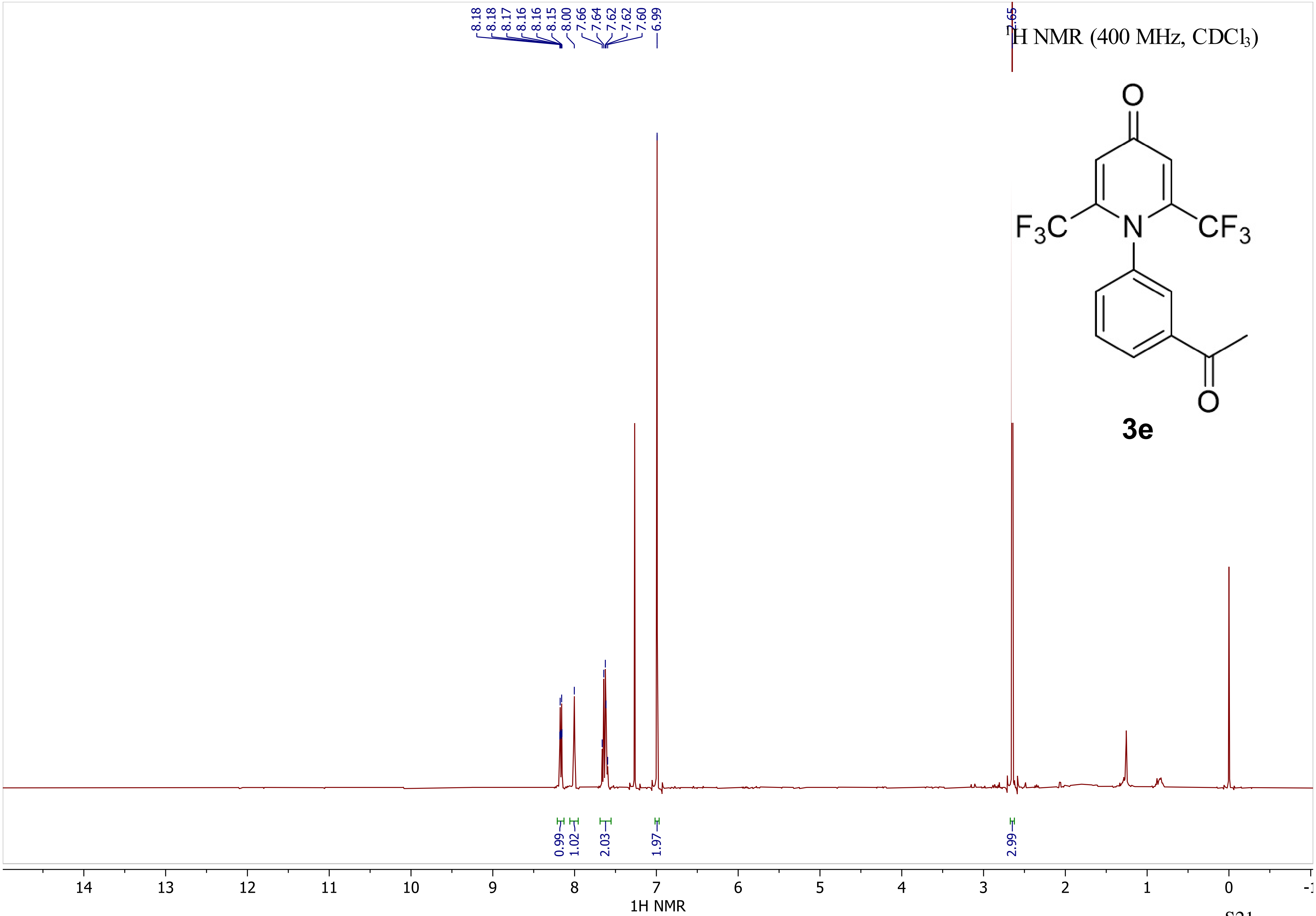
8.18
8.18
8.17
8.16
8.16
8.15
8.00
7.66
7.64
7.62
7.62
7.60
6.99
2.65
1H NMR (400 MHz, CDCl3)
O
F3C
N
CF3
O
3e
0.99
1.02
2.03
1.97
2.99
14
13
12
11
10
9
8
7
6
5
4
3
2
1
0
1H NMR

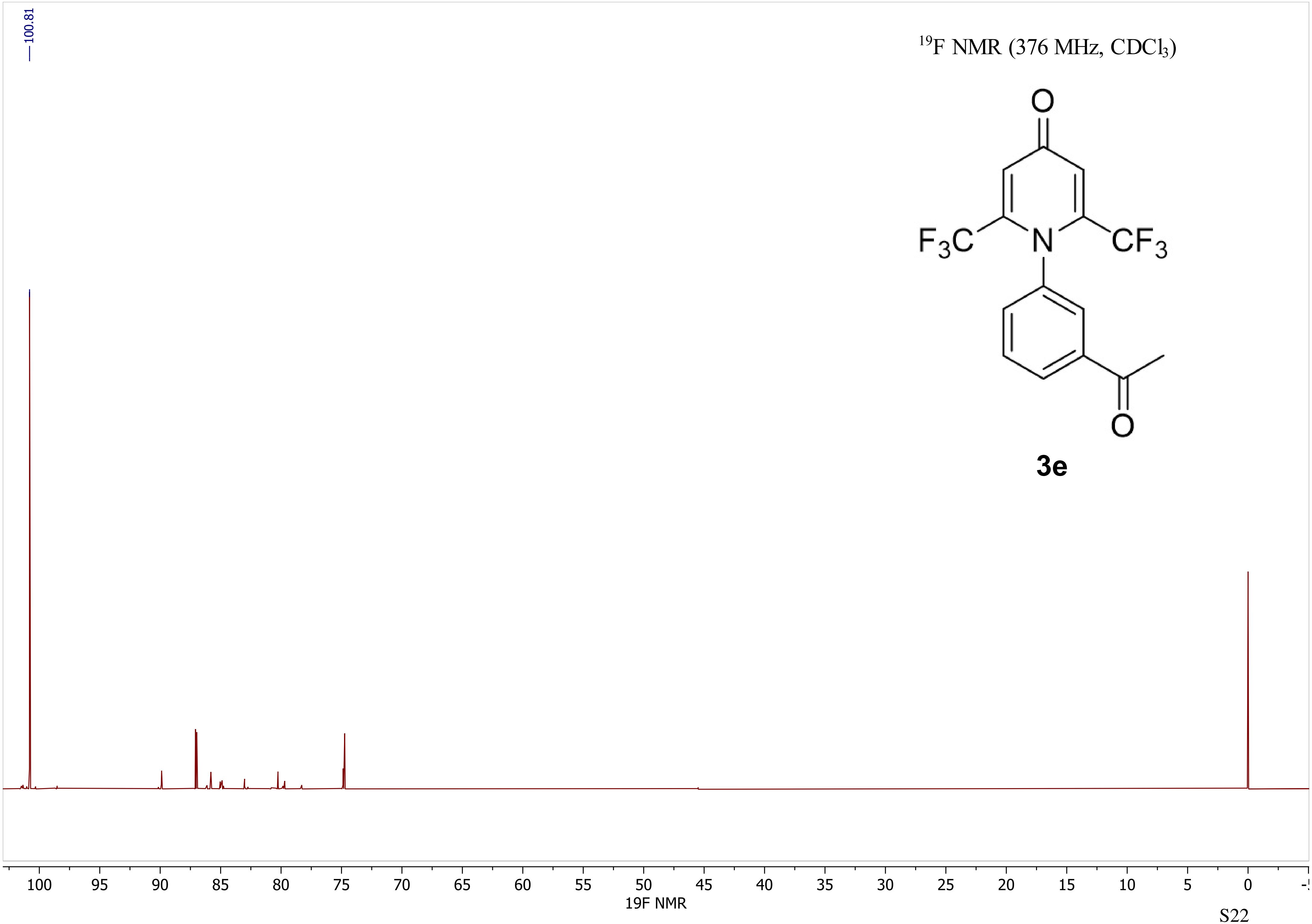

^{19}F NMR (376 MHz, $CDCl_3$)

3e

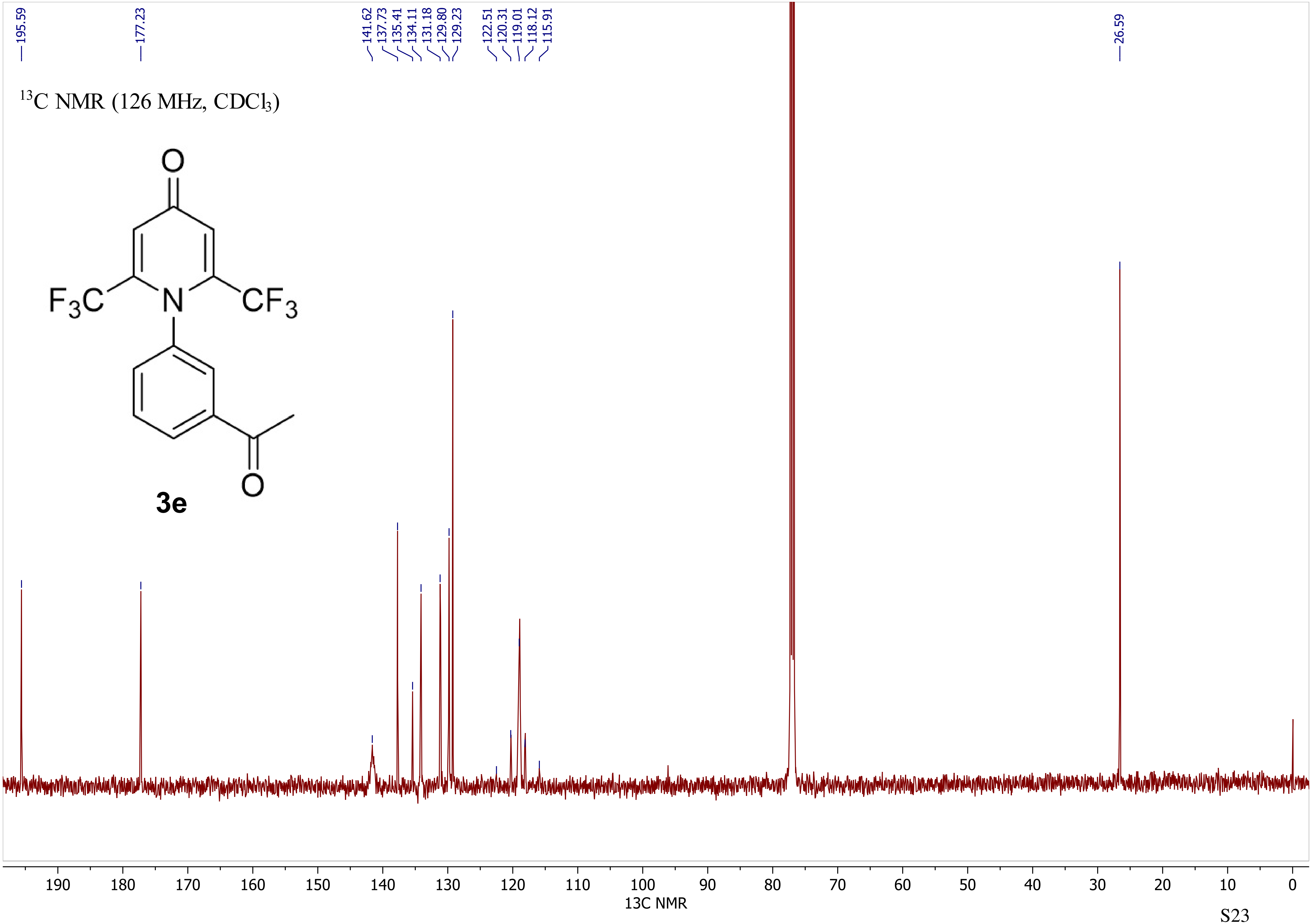
195.59
177.23
141.62
137.73
135.41
134.11
131.18
129.80
129.23
122.51
120.31
119.01
118.12
115.91
26.59
^{13}C NMR (126 MHz, $CDCl_3$)
O
N
F_3C
CF_3
O
3e
190 180 170 160 150 140 130 120 110 100 90 80 70 60 50 40 30 20 10 0
13C NMR

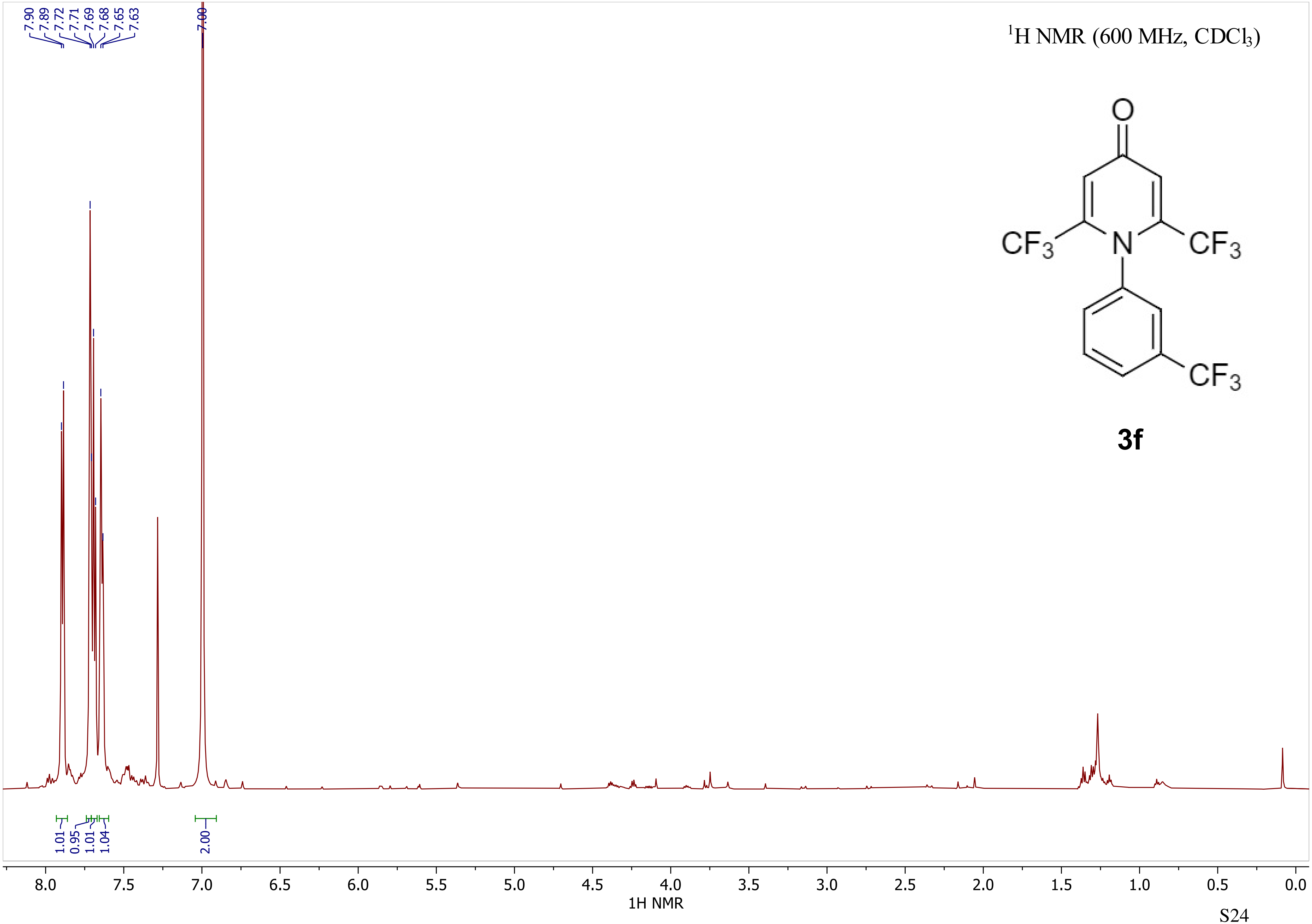

^{1}H NMR (600 MHz, CDCl$_3$)
7.90
7.89
7.72
7.71
7.69
7.68
7.65
7.63
7.00
1.01
0.95
1.01
1.04
2.00
O
N
CF3
CF3
CF3
3f
8.0
7.5
7.0
6.5
6.0
5.5
5.0
4.5
4.0
3.5
3.0
2.5
2.0
1.5
1.0
0.5
0.0
1H NMR

^{19}F NMR (376 MHz, $CDCl_3$)

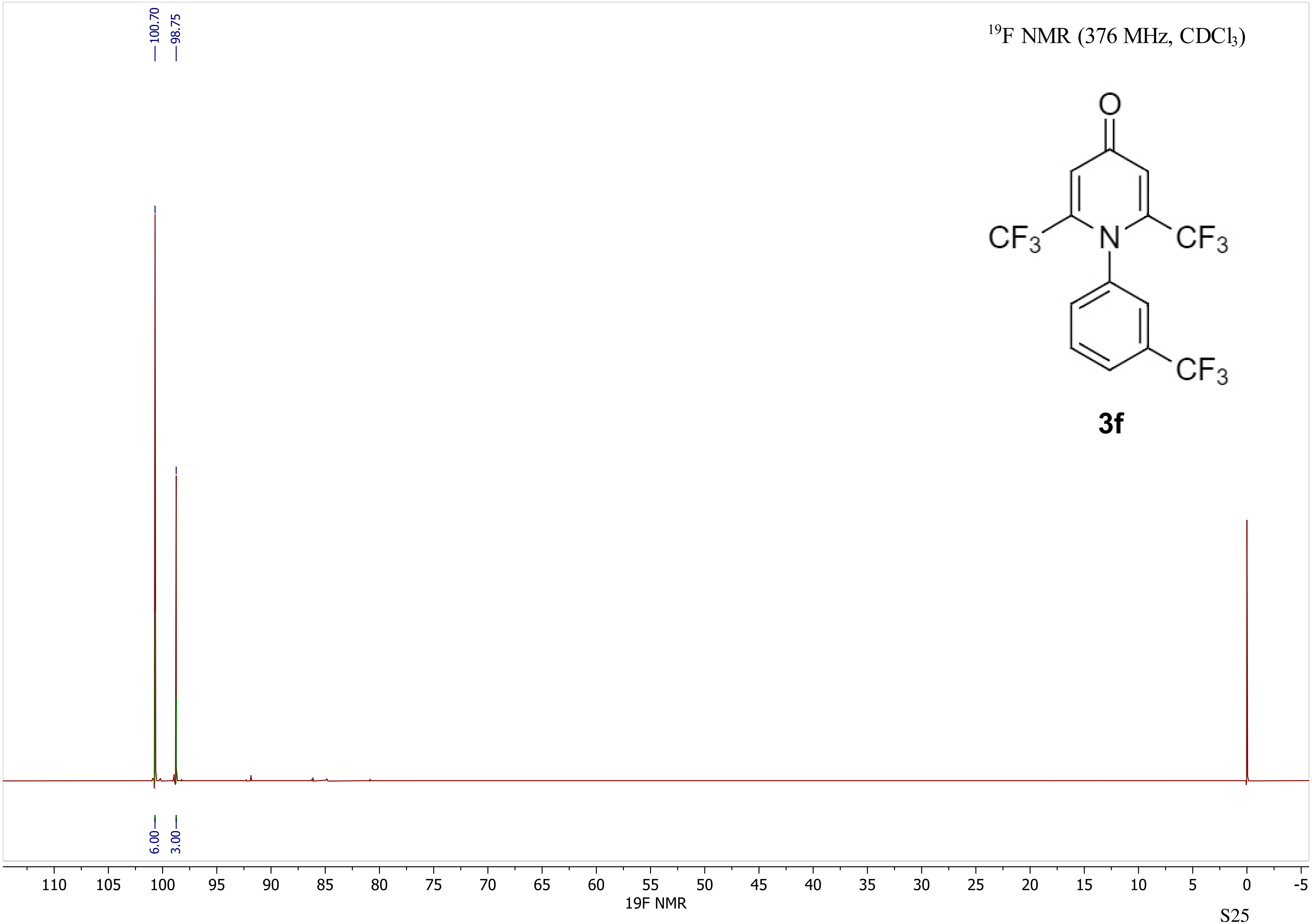

3f

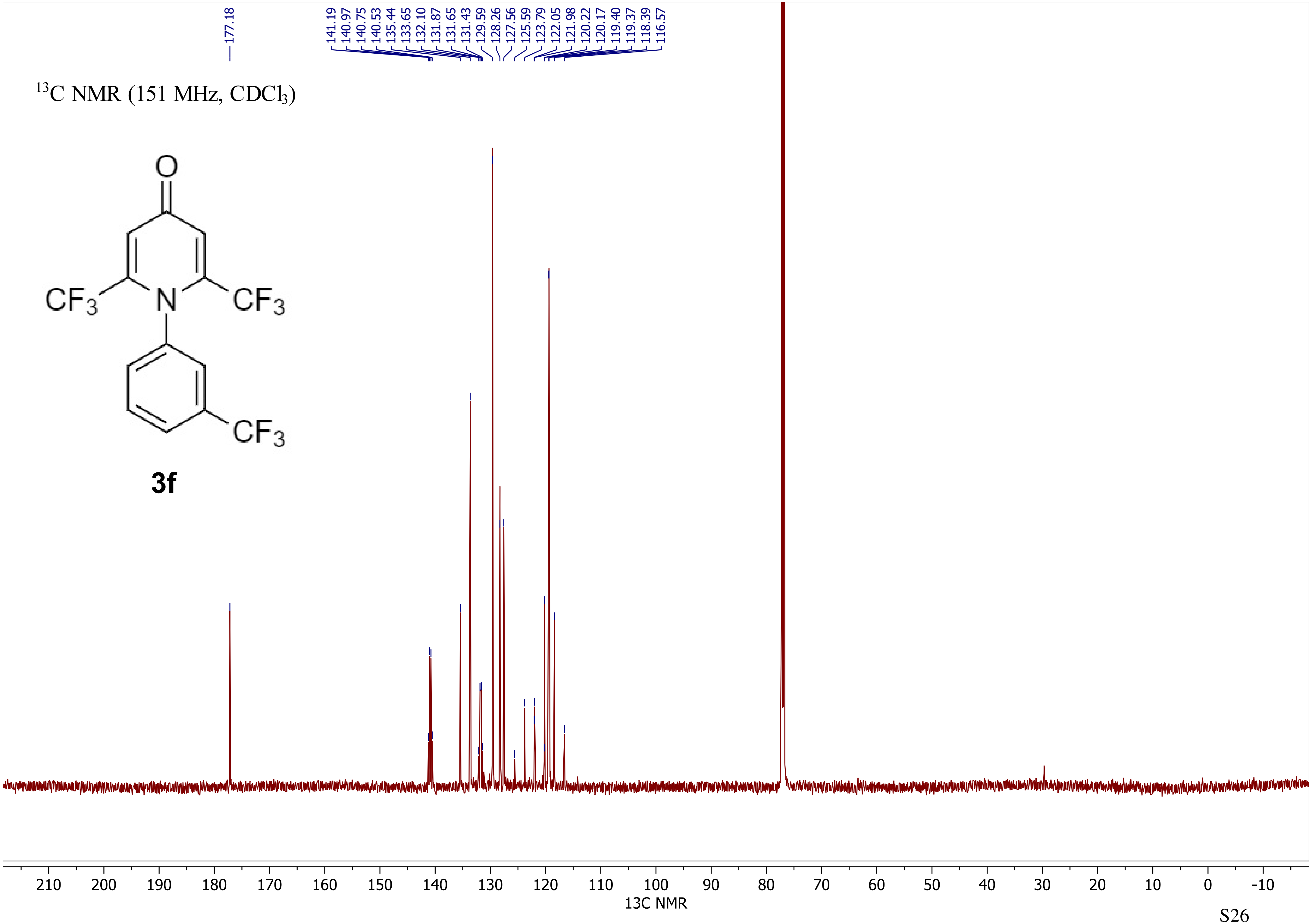

177.18
141.19
140.97
140.75
140.53
135.44
133.65
132.10
131.87
131.65
131.43
129.59
128.26
127.56
125.59
123.79
122.05
121.98
120.22
120.17
119.40
119.37
118.39
116.57
13C NMR (151 MHz, CDCl3)
O
N
CF3
CF3
CF3
3f
210 200 190 180 170 160 150 140 130 120 110 100 90 80 70 60 50 40 30 20 10 0 -10
13C NMR

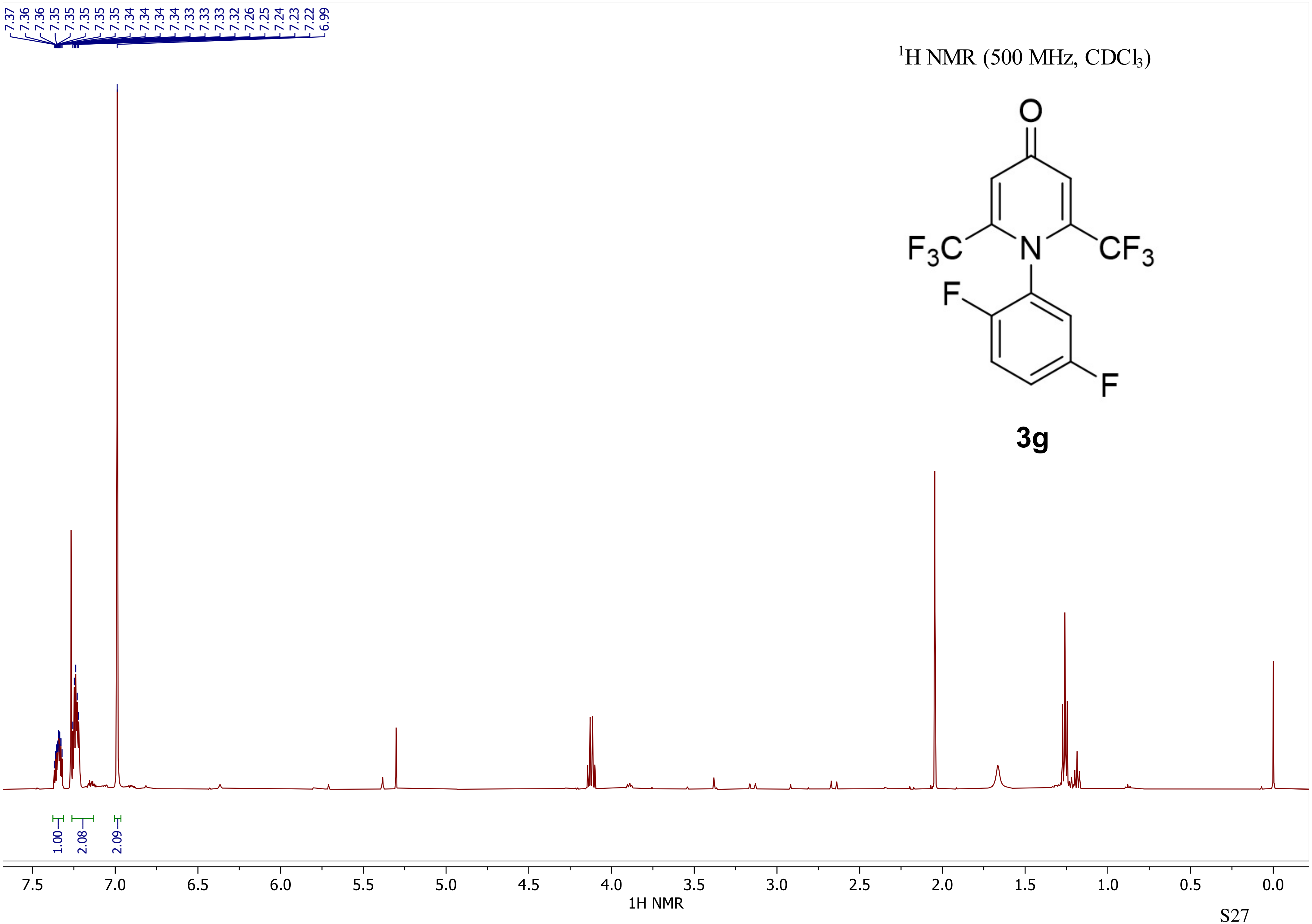

7.37
7.36
7.36
7.35
7.35
7.35
7.35
7.35
7.34
7.34
7.34
7.34
7.33
7.33
7.33
7.32
7.26
7.25
7.24
7.23
7.22
6.99
^{1}H NMR (500 MHz, CDCl$_3$)
O
F_3C
N
CF_3
F
F
3g
1.00
2.08
2.09
7.5
7.0
6.5
6.0
5.5
5.0
4.5
4.0
3.5
3.0
2.5
2.0
1.5
1.0
0.5
0.0
1H NMR

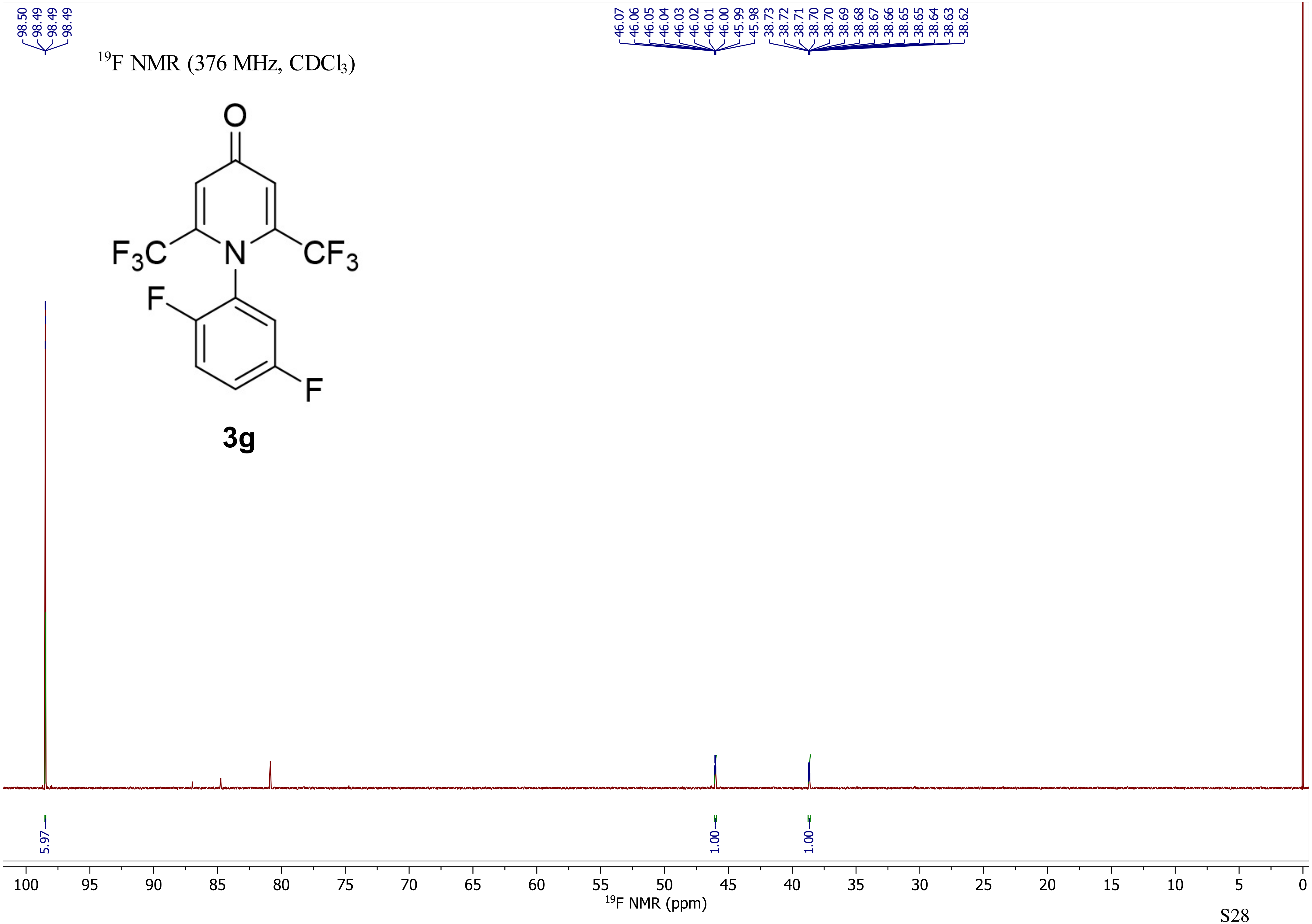
98.50
98.49
98.49
98.49
46.07
46.06
46.05
46.04
46.03
46.02
46.01
46.00
45.99
45.98
38.73
38.72
38.71
38.70
38.70
38.69
38.68
38.67
38.66
38.65
38.65
38.64
38.63
38.62
19F NMR (376 MHz, CDCl3)
O
F3C
N
CF3
F
F
3g
5.97
1.00
1.00
100
95
90
85
80
75
70
65
60
55
50
45
40
35
30
25
20
15
10
5
0
19F NMR (ppm)

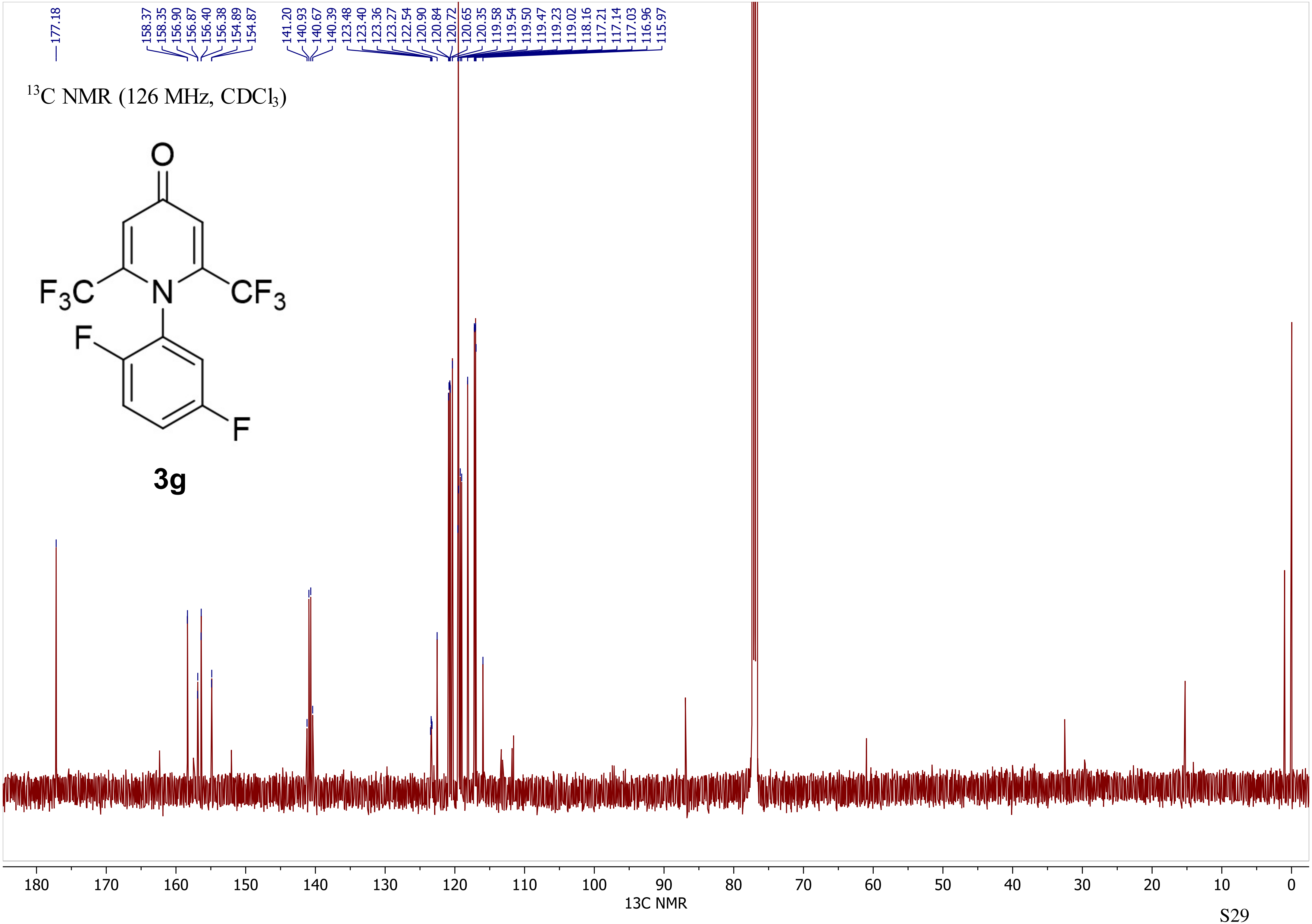

177.18
158.37
158.35
156.90
156.87
156.40
156.38
154.89
154.87
141.20
140.93
140.67
140.39
123.48
123.40
123.36
123.27
122.54
120.90
120.84
120.72
120.65
120.35
119.58
119.54
119.50
119.47
119.23
119.02
118.16
117.21
117.14
117.03
116.96
115.97
^{13}C NMR (126 MHz, CDCl$_3$)
180 170 160 150 140 130 120 110 100 90 80 70 60 50 40 30 20 10 0
13C NMR

3g

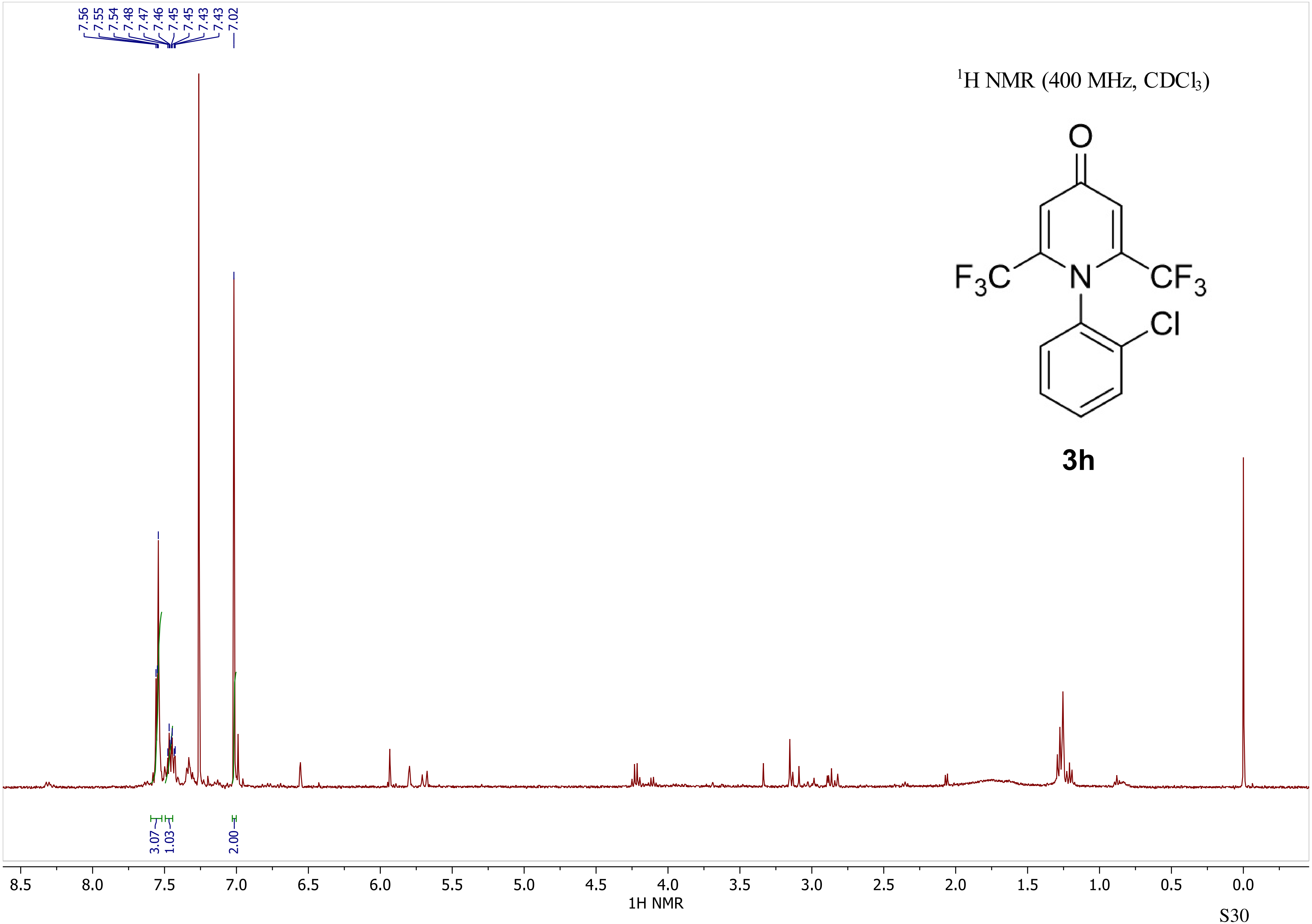

7.56
7.55
7.54
7.48
7.47
7.46
7.45
7.45
7.43
7.43
7.02
^{1}H NMR (400 MHz, $CDCl_3$)
O
F_3C
N
CF_3
Cl
3h
3.07
1.03
2.00
8.5
8.0
7.5
7.0
6.5
6.0
5.5
5.0
4.5
4.0
3.5
3.0
2.5
2.0
1.5
1.0
0.5
0.0
1H NMR

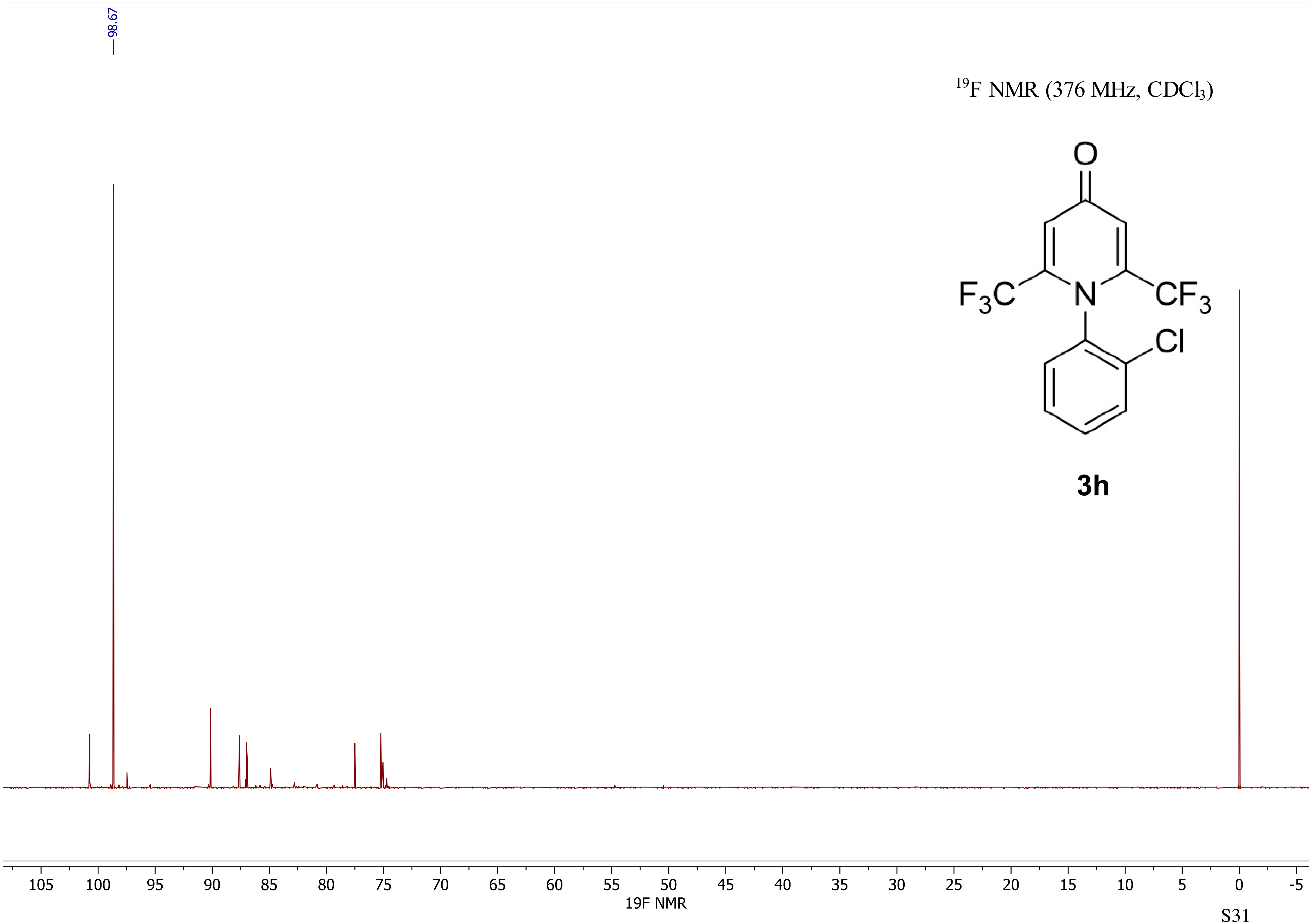
98.67
^{19}F NMR (376 MHz, $CDCl_3$)
O
F_3C
N
CF_3
Cl
3h
105 100 95 90 85 80 75 70 65 60 55 50 45 40 35 30 25 20 15 10 5 0 -5
19F NMR

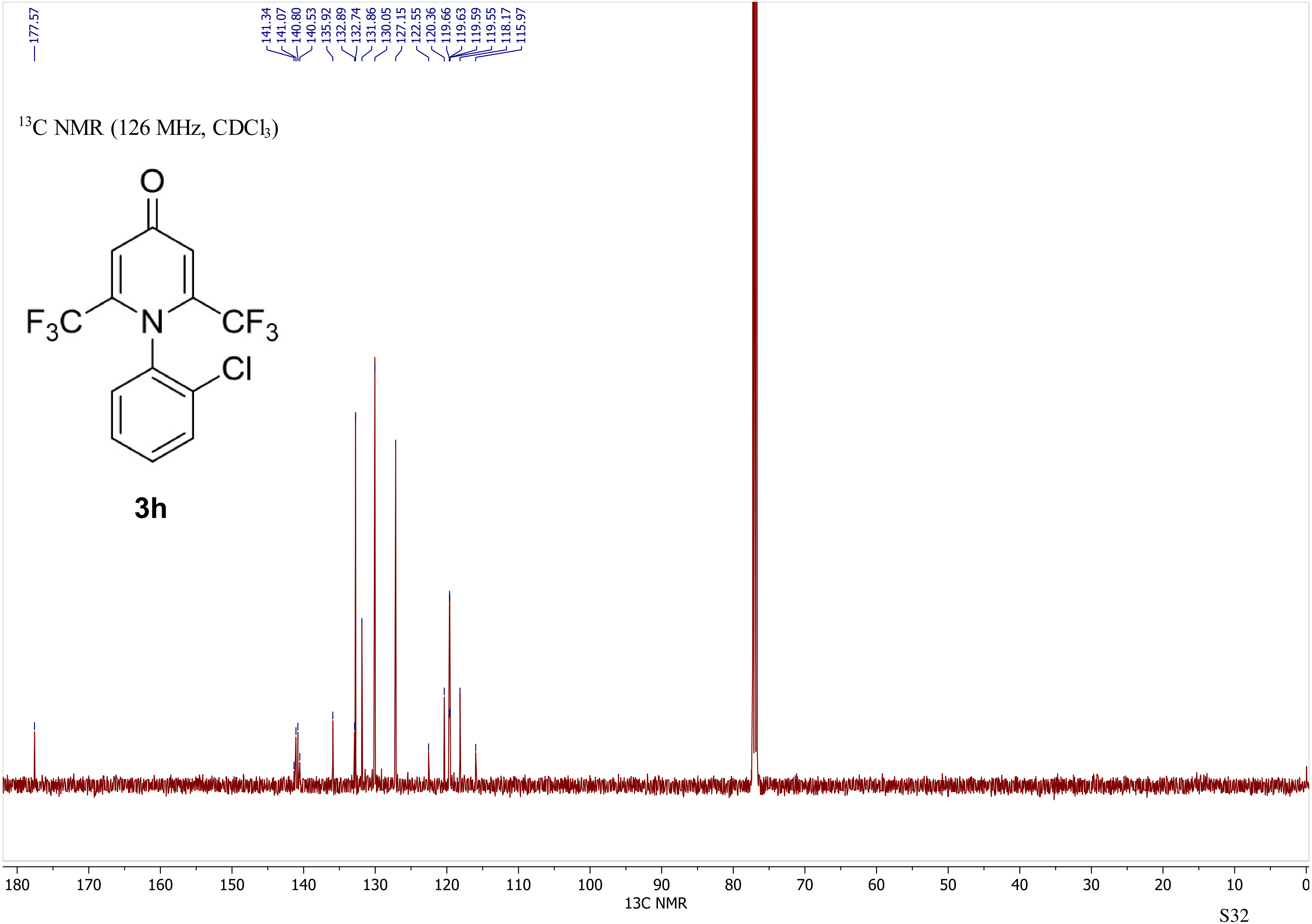
177.57
141.34
141.07
140.80
140.53
135.92
132.89
132.74
131.86
130.05
127.15
122.55
120.36
119.66
119.63
119.59
119.55
118.17
115.97
^{13}C NMR (126 MHz, $CDCl_3$)
O
F_3C
N
CF_3
Cl
3h
180
170
160
150
140
130
120
110
100
90
80
70
60
50
40
30
20
10
0
13C NMR

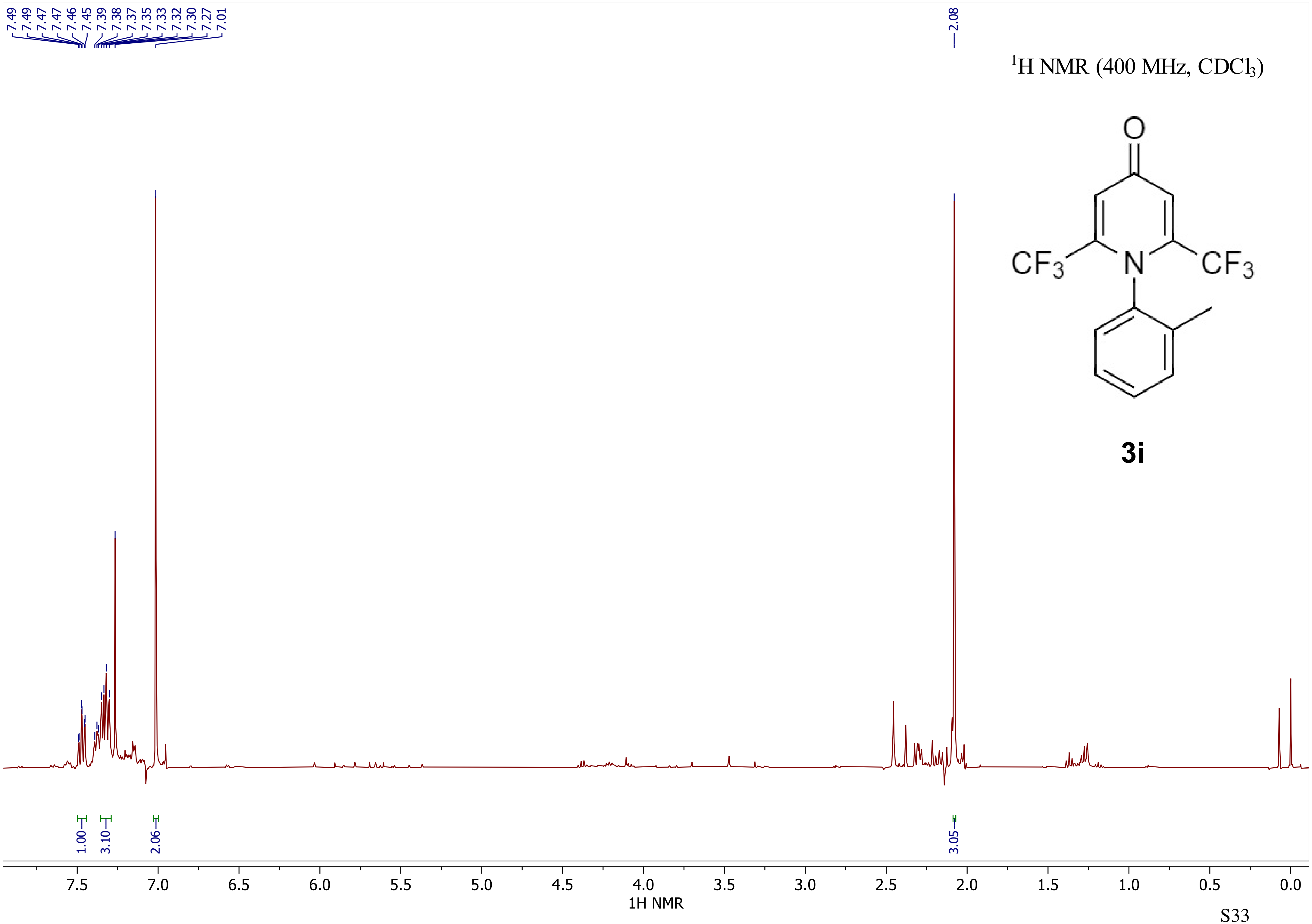

7.49
7.49
7.47
7.47
7.46
7.45
7.39
7.38
7.37
7.35
7.33
7.32
7.30
7.27
7.01
2.08
^{1}H NMR (400 MHz, $CDCl_3$)
O
CF_3
N
CF_3
3i
1.00
3.10
2.06
3.05
7.5
7.0
6.5
6.0
5.5
5.0
4.5
4.0
3.5
3.0
2.5
2.0
1.5
1.0
0.5
0.0
1H NMR

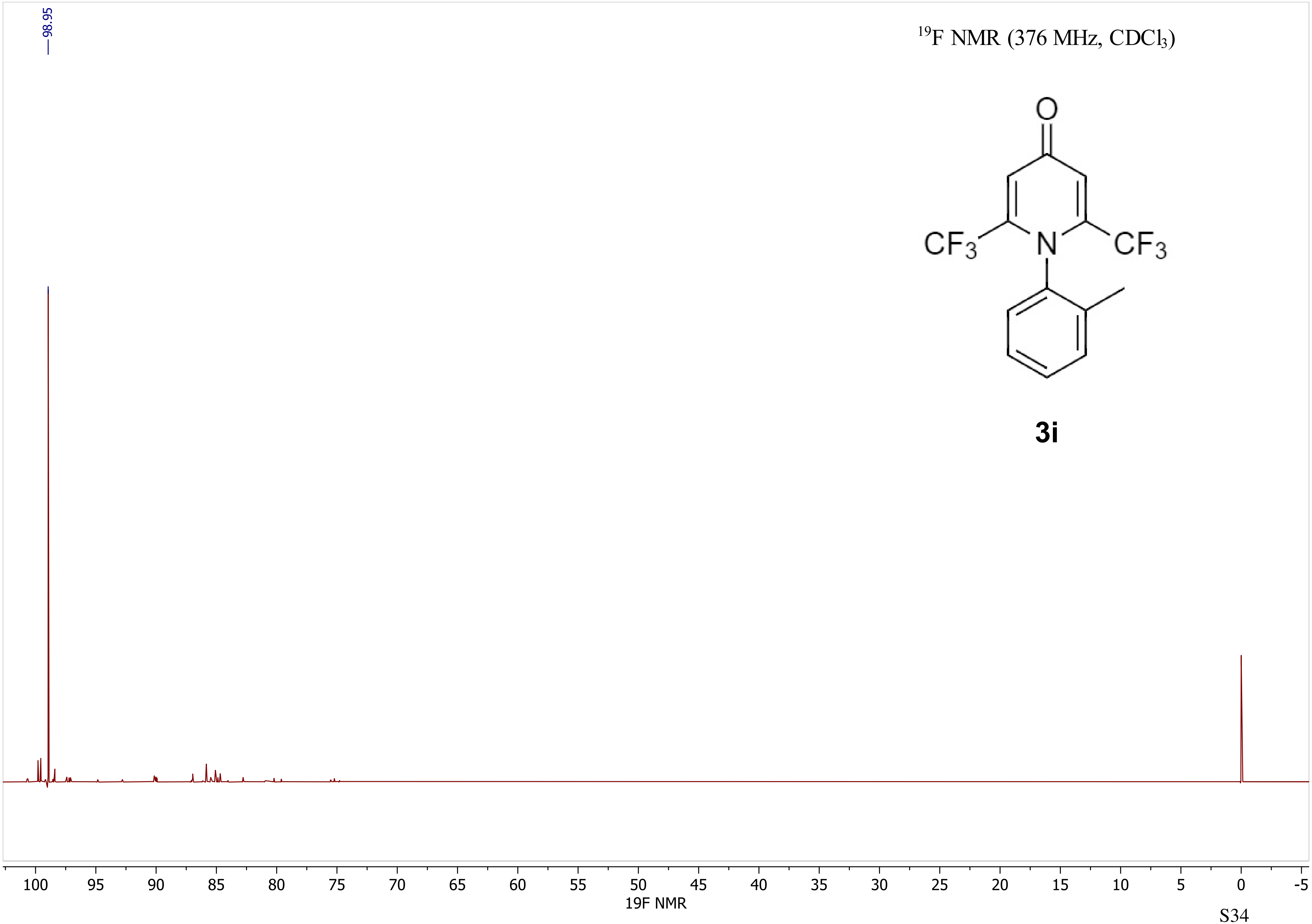
—98.95
^{19}F NMR (376 MHz, $CDCl_3$)
O
N
CF_3
CF_3
3i
100 95 90 85 80 75 70 65 60 55 50 45 40 35 30 25 20 15 10 5 0 -5
19F NMR

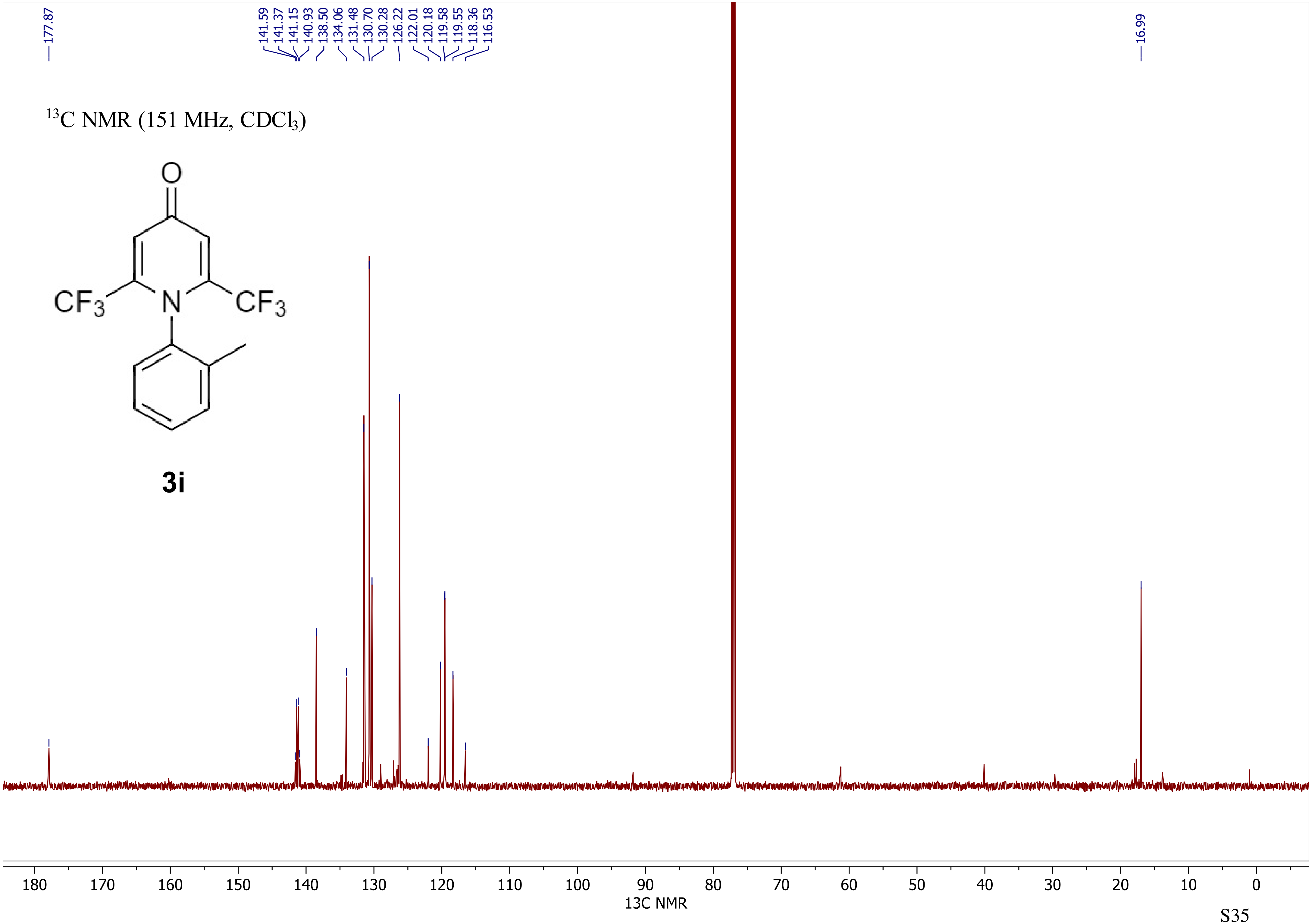
177.87
141.59
141.37
141.15
140.93
138.50
134.06
131.48
130.70
130.28
126.22
122.01
120.18
119.58
119.55
118.36
116.53
16.99
13C NMR (151 MHz, CDCl3)
O
N
CF3
CF3
3i
180 170 160 150 140 130 120 110 100 90 80 70 60 50 40 30 20 10 0
13C NMR

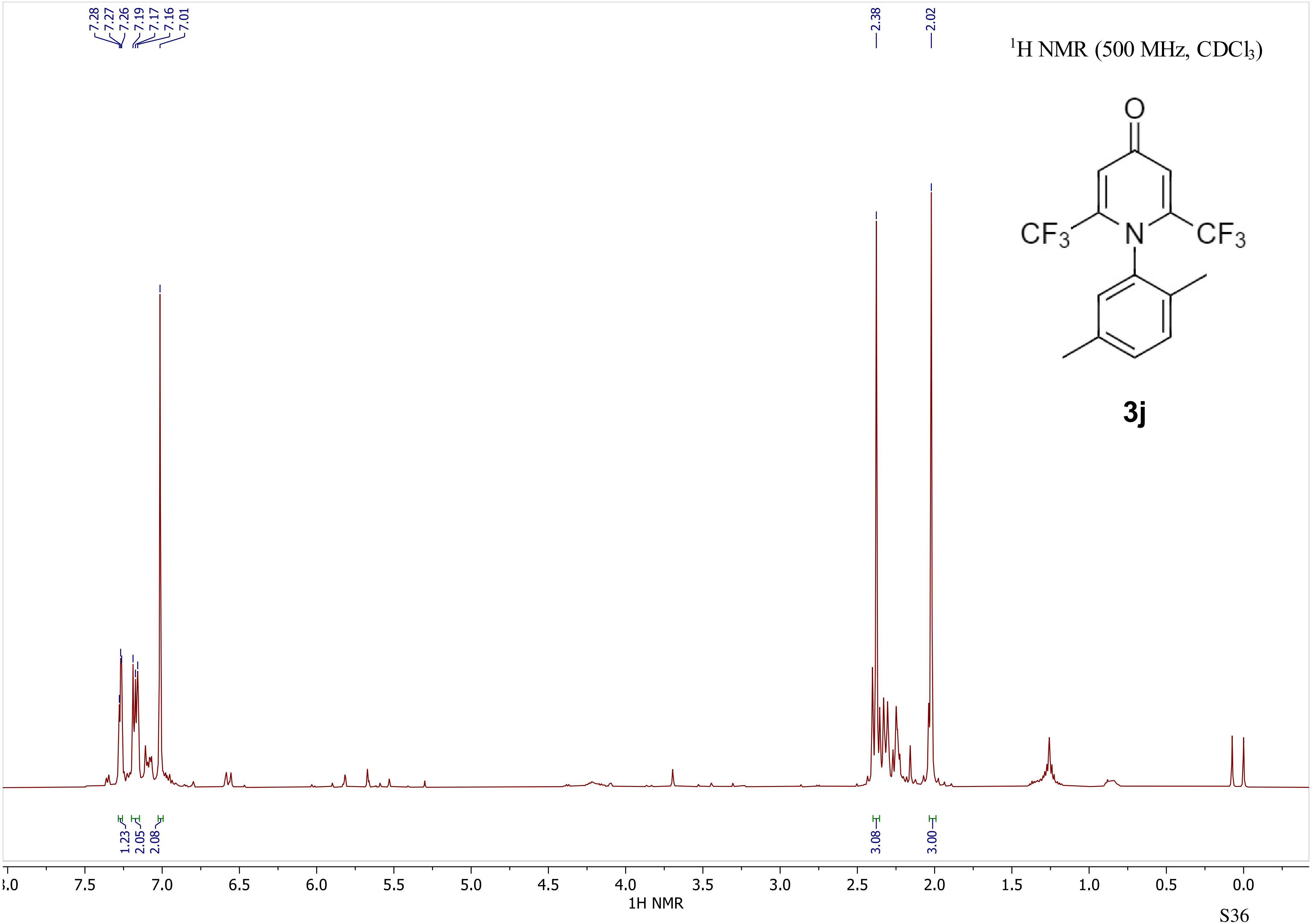

7.28
7.27
7.26
7.19
7.17
7.16
7.01
2.38
2.02
^{1}H NMR (500 MHz, CDCl$_3$)
O
CF3
N
CF3
3j
1.23
2.05
2.08
3.08
3.00
8.0
7.5
7.0
6.5
6.0
5.5
5.0
4.5
4.0
3.5
3.0
2.5
2.0
1.5
1.0
0.5
0.0
1H NMR

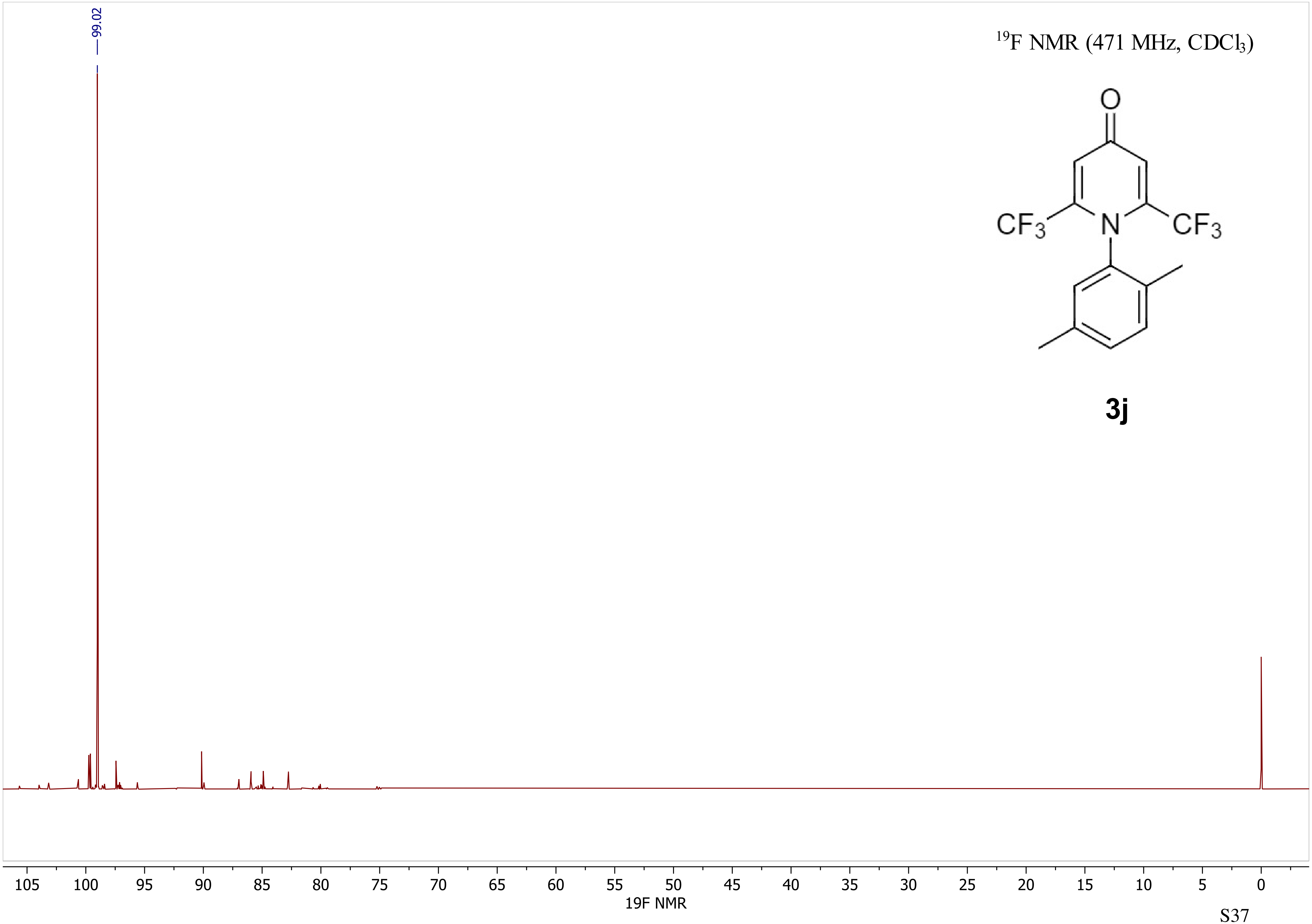

99.02
19F NMR (471 MHz, CDCl3)
105
100
95
90
85
80
75
70
65
60
55
50
45
40
35
30
25
20
15
10
5
0
19F NMR
O
CF3
N
CF3

3j

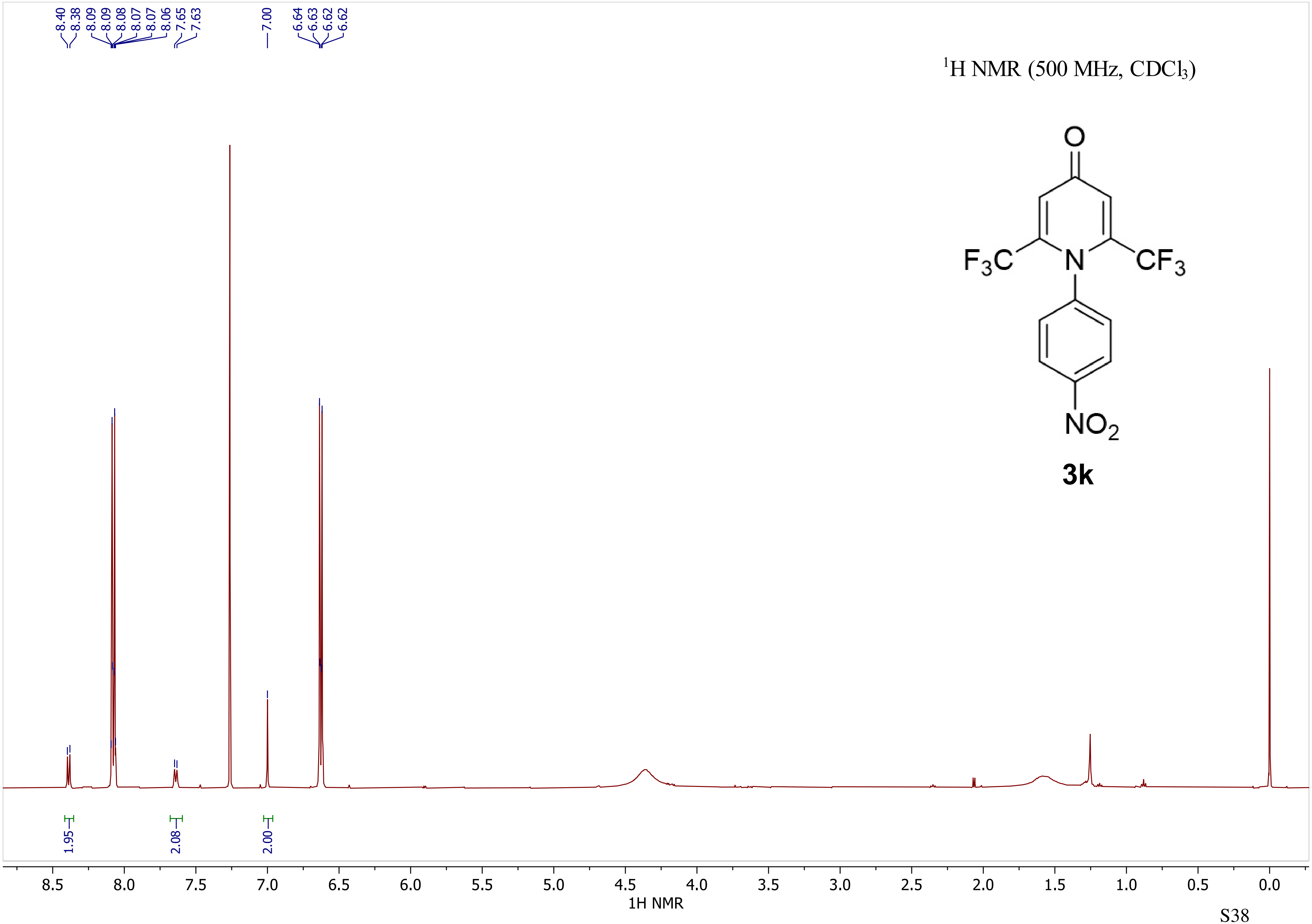

8.40
8.38
8.09
8.09
8.08
8.07
8.07
8.06
7.65
7.63
7.00
6.64
6.63
6.62
6.62
1H NMR (500 MHz, CDCl3)
O
F3C
N
CF3
NO2
3k
1.95
2.08
2.00
8.5
8.0
7.5
7.0
6.5
6.0
5.5
5.0
4.5
4.0
3.5
3.0
2.5
2.0
1.5
1.0
0.5
0.0
1H NMR

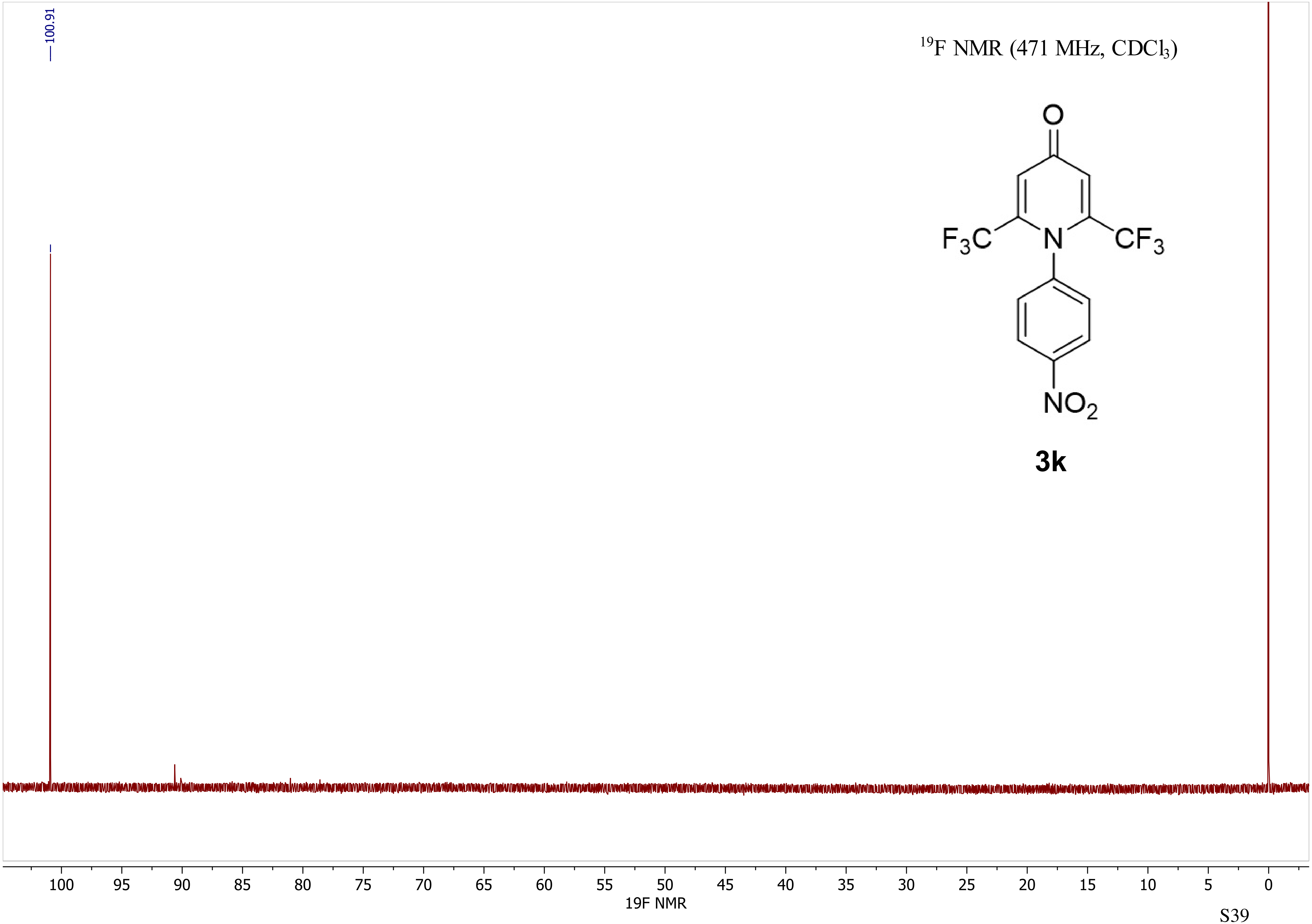

-100.91
^{19}F NMR (471 MHz, $CDCl_3$)
O
F_3C
N
CF_3
NO_2
3k
100
95
90
85
80
75
70
65
60
55
50
45
40
35
30
25
20
15
10
5
0
19F NMR

^{1}H NMR (400 MHz, $CDCl_3$)

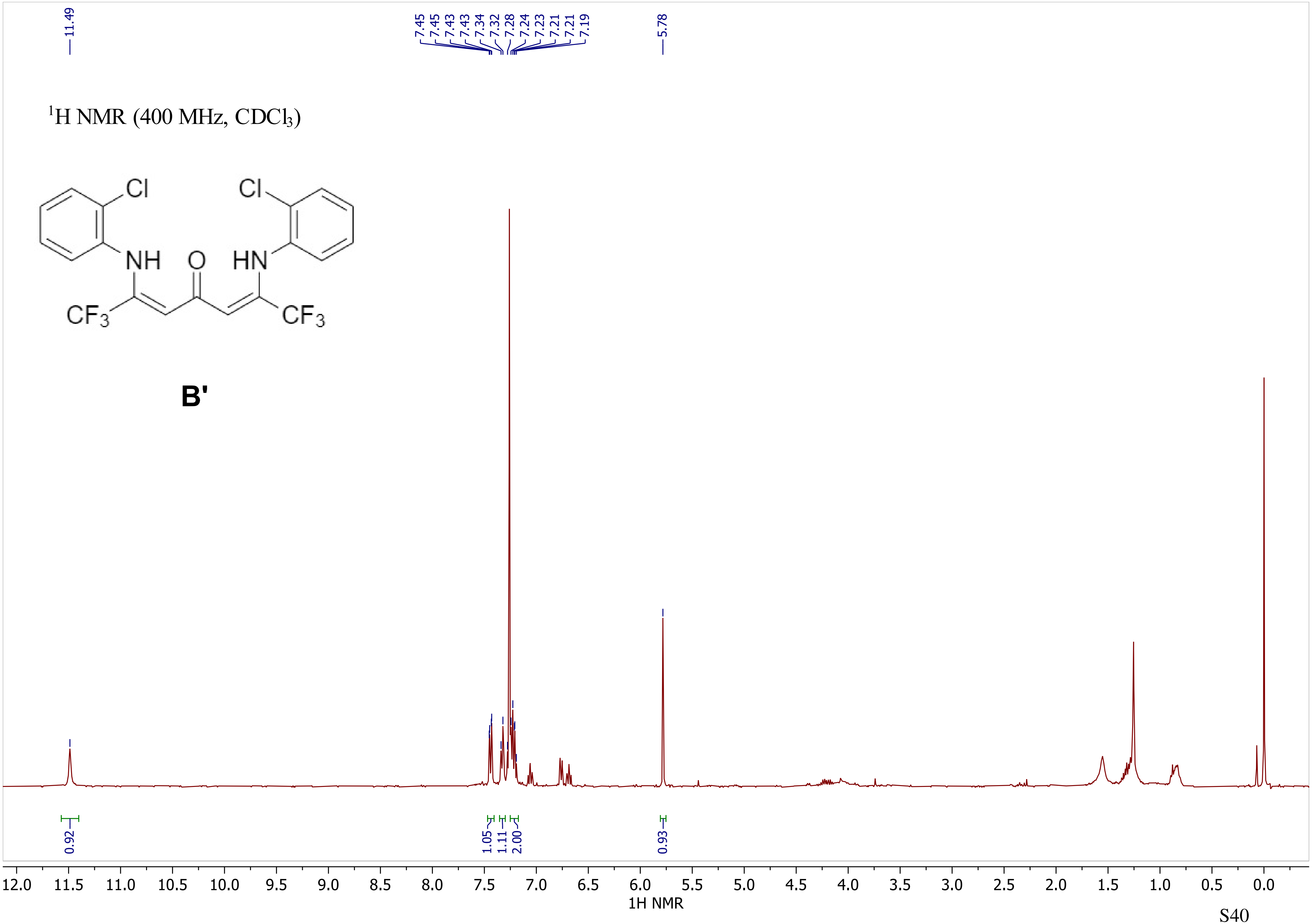

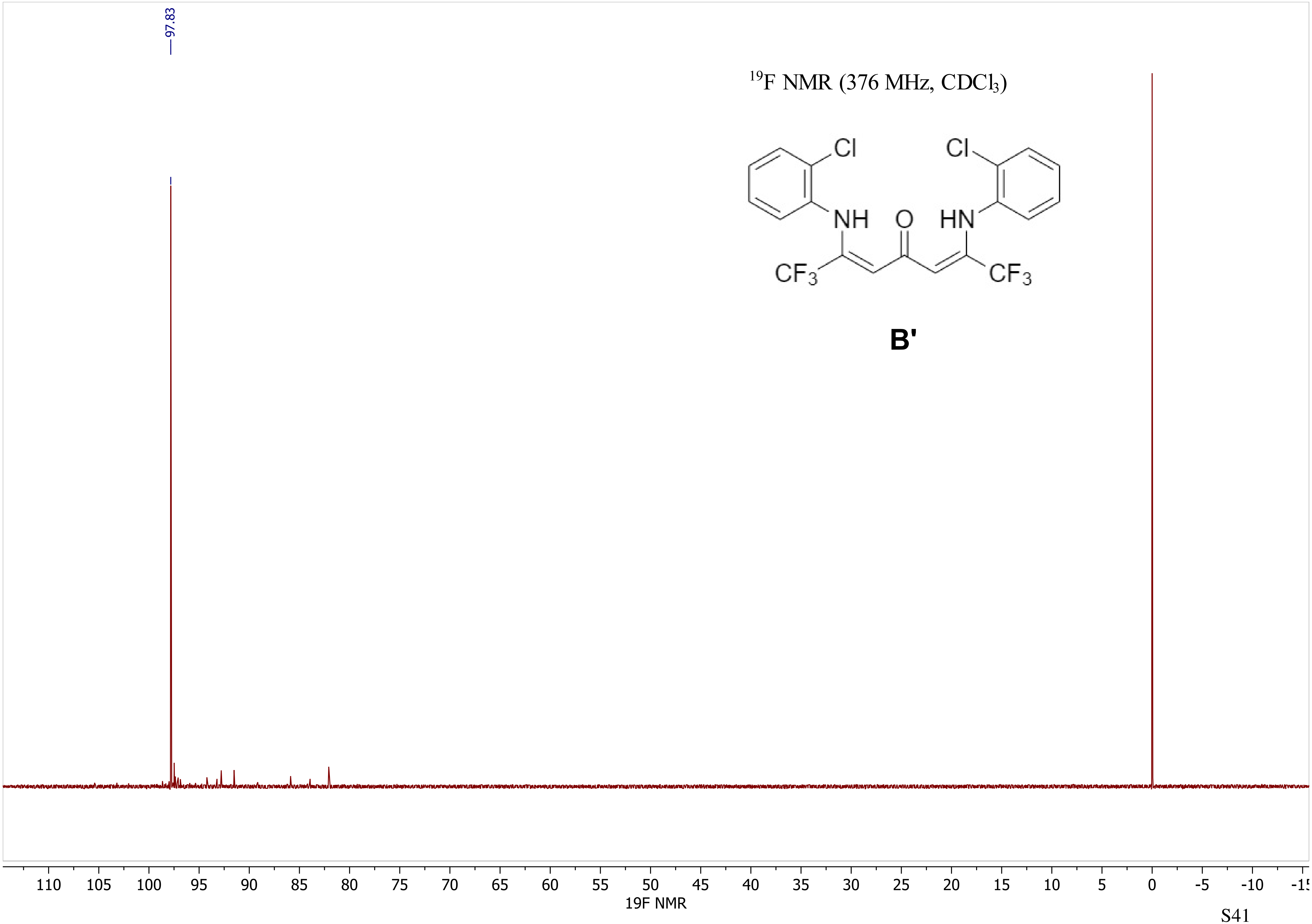
-97.83
19F NMR (376 MHz, CDCl3)
Cl
Cl
NH
O
HN
CF3
CF3
B'
110 105 100 95 90 85 80 75 70 65 60 55 50 45 40 35 30 25 20 15 10 5 0 -5 -10
19F NMR

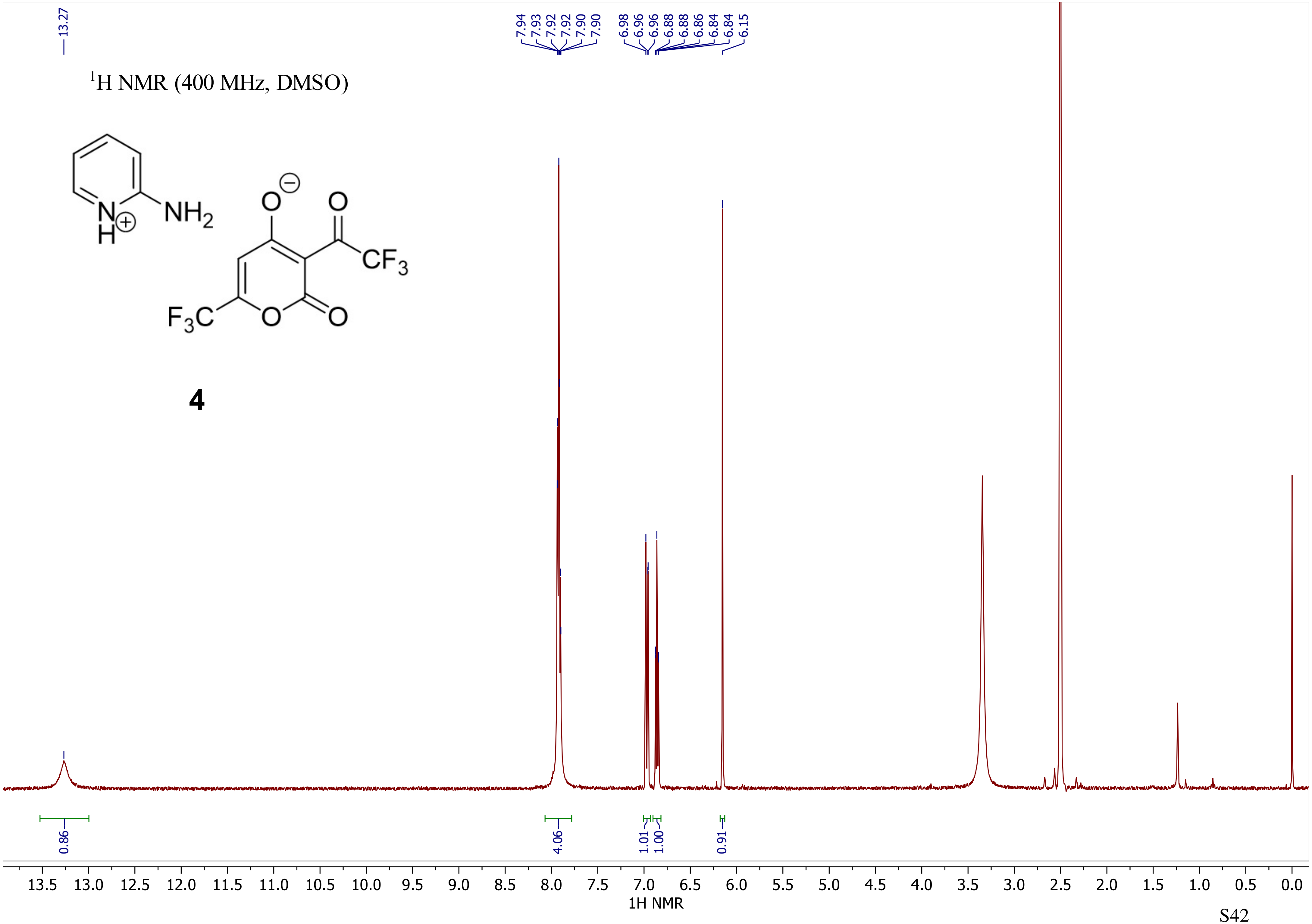

1H NMR (400 MHz, DMSO)
4
13.27
7.94 7.93 7.92 7.92 7.90 7.90
6.98 6.96 6.96 6.88 6.88 6.86 6.84 6.84 6.15
0.86
4.06
1.01
1.00
0.91
13.5 13.0 12.5 12.0 11.5 11.0 10.5 10.0 9.5 9.0 8.5 8.0 7.5 7.0 6.5 6.0 5.5 5.0 4.5 4.0 3.5 3.0 2.5 2.0 1.5 1.0 0.5 0.0
1H NMR

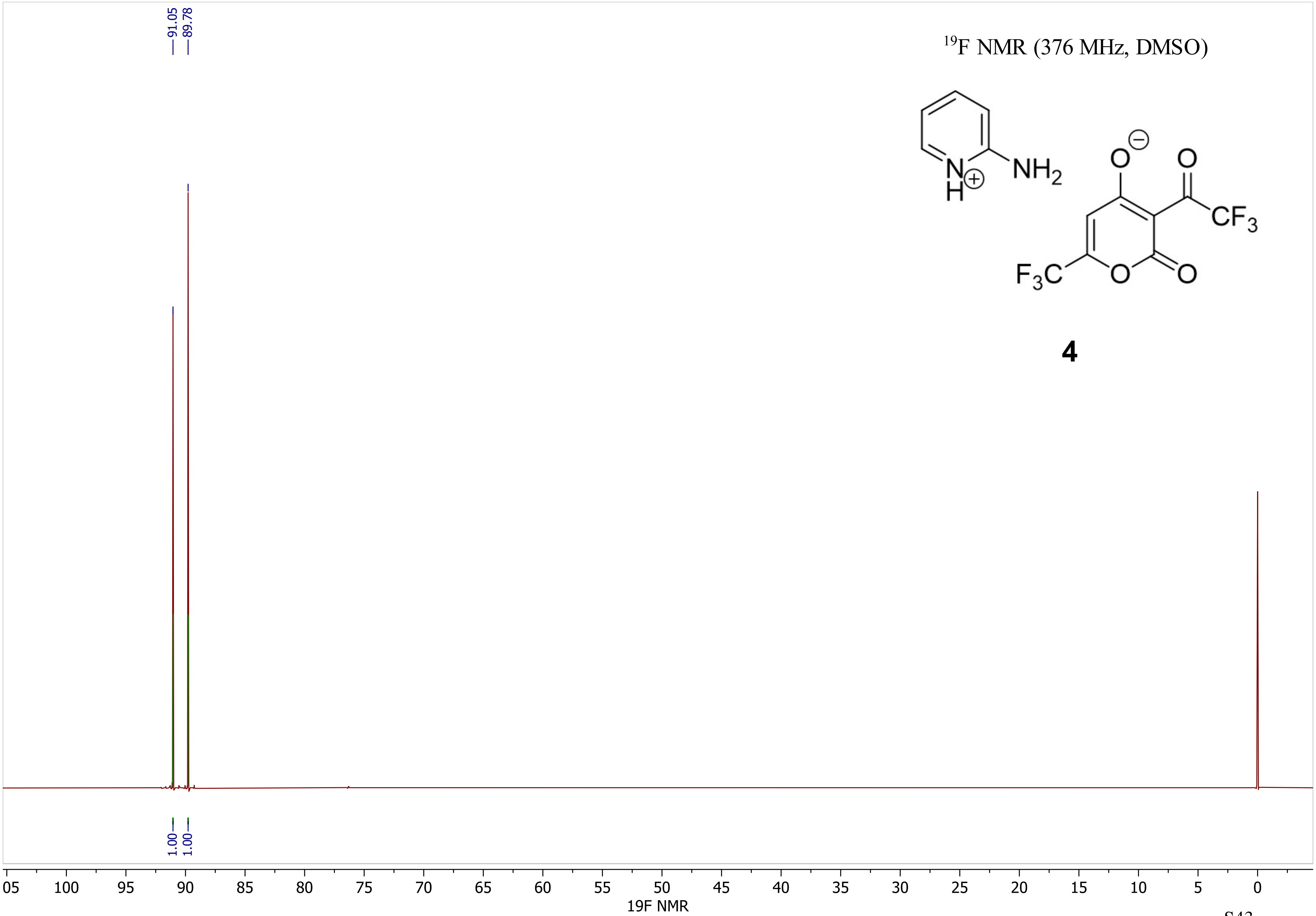

^{19}F NMR (376 MHz, DMSO)

4

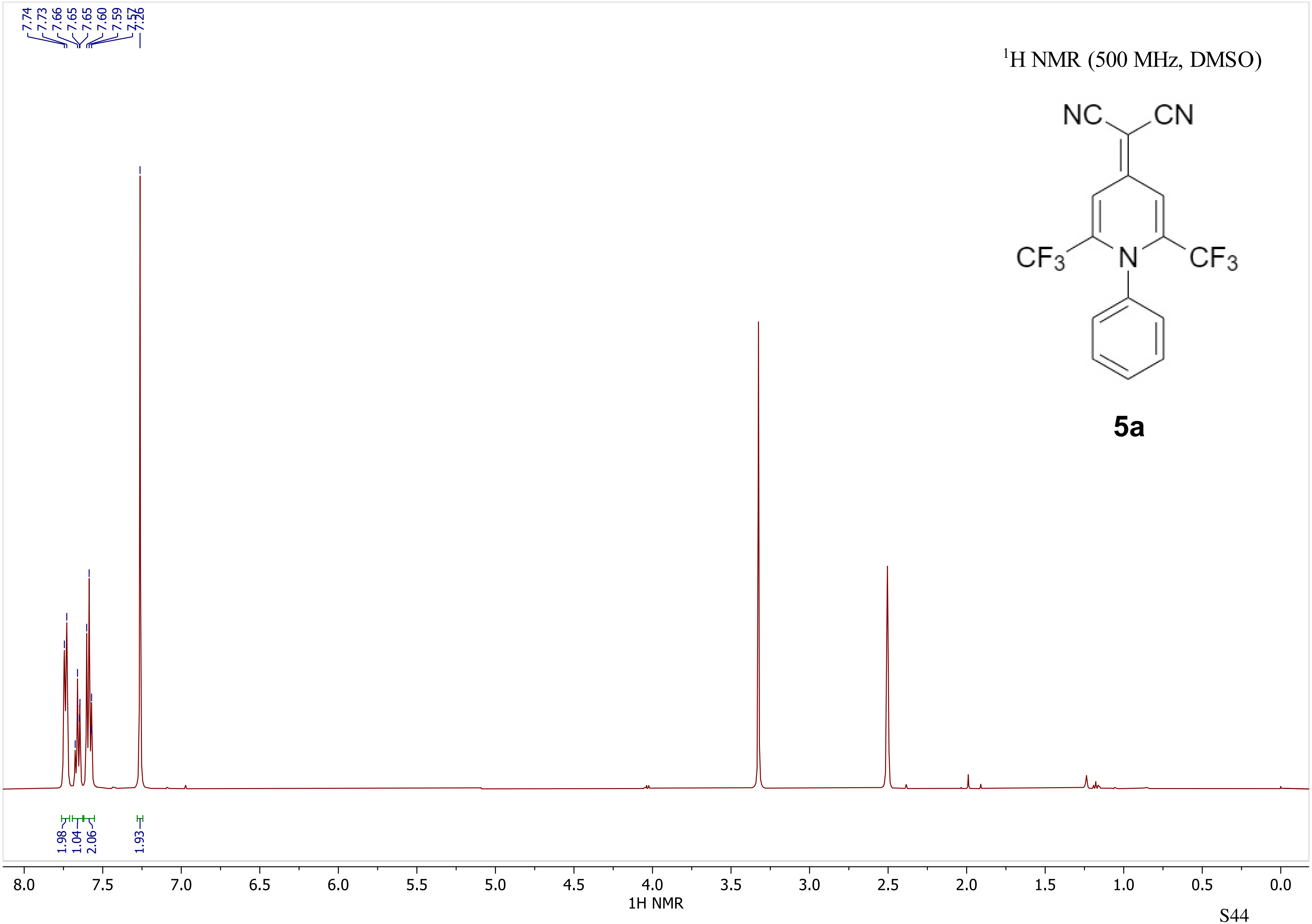

7.74
7.73
7.66
7.65
7.65
7.60
7.59
7.57
7.26
^{1}H NMR (500 MHz, DMSO)
NC
CN
CF_3
N
CF_3
5a
1.98
1.04
2.06
1.93
8.0
7.5
7.0
6.5
6.0
5.5
5.0
4.5
4.0
3.5
3.0
2.5
2.0
1.5
1.0
0.5
0.0
1H NMR

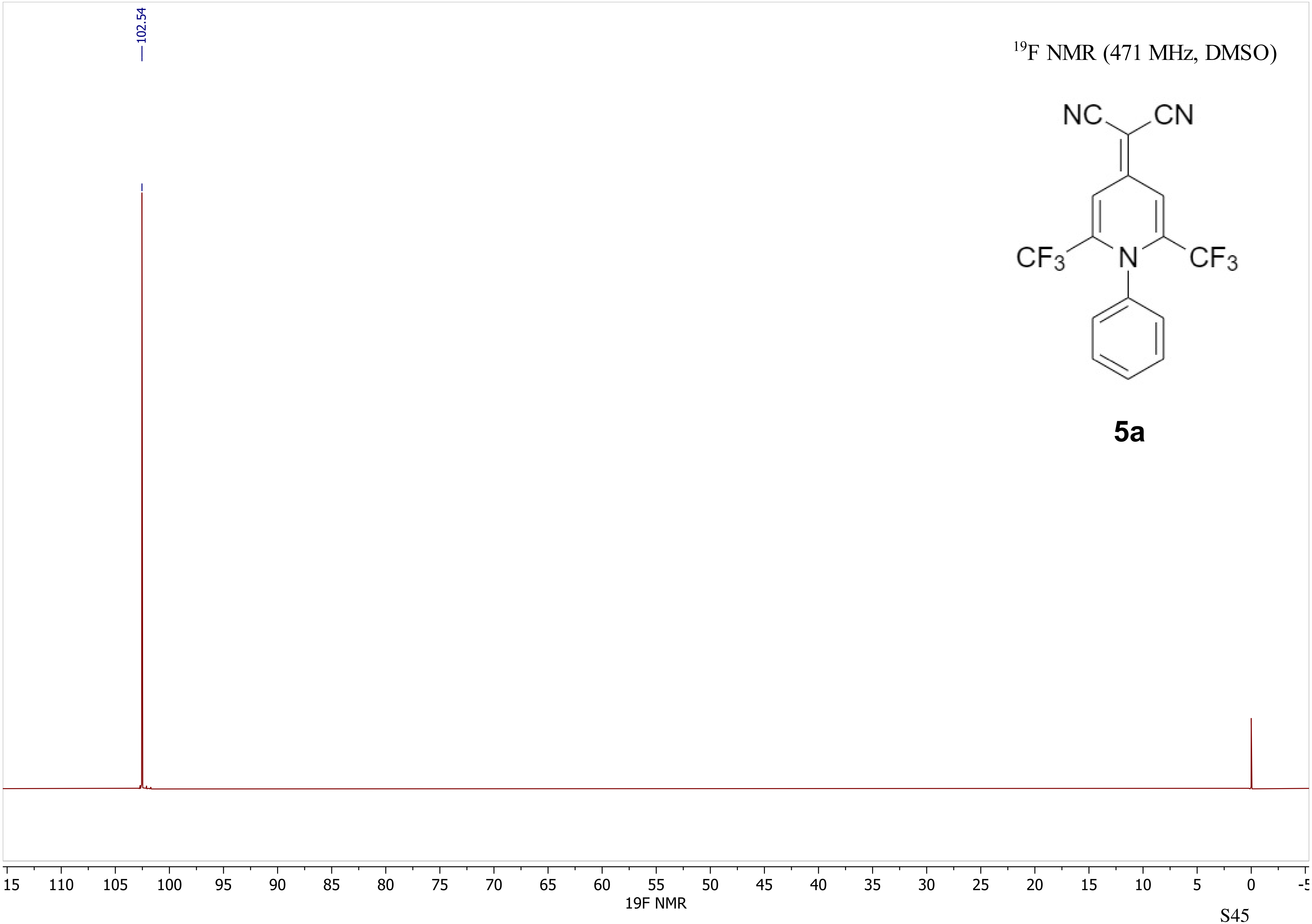

102.54
19F NMR (471 MHz, DMSO)
NC
CN
CF3
N
CF3
5a
15
110
105
100
95
90
85
80
75
70
65
60
55
50
45
40
35
30
25
20
15
10
5
0
19F NMR

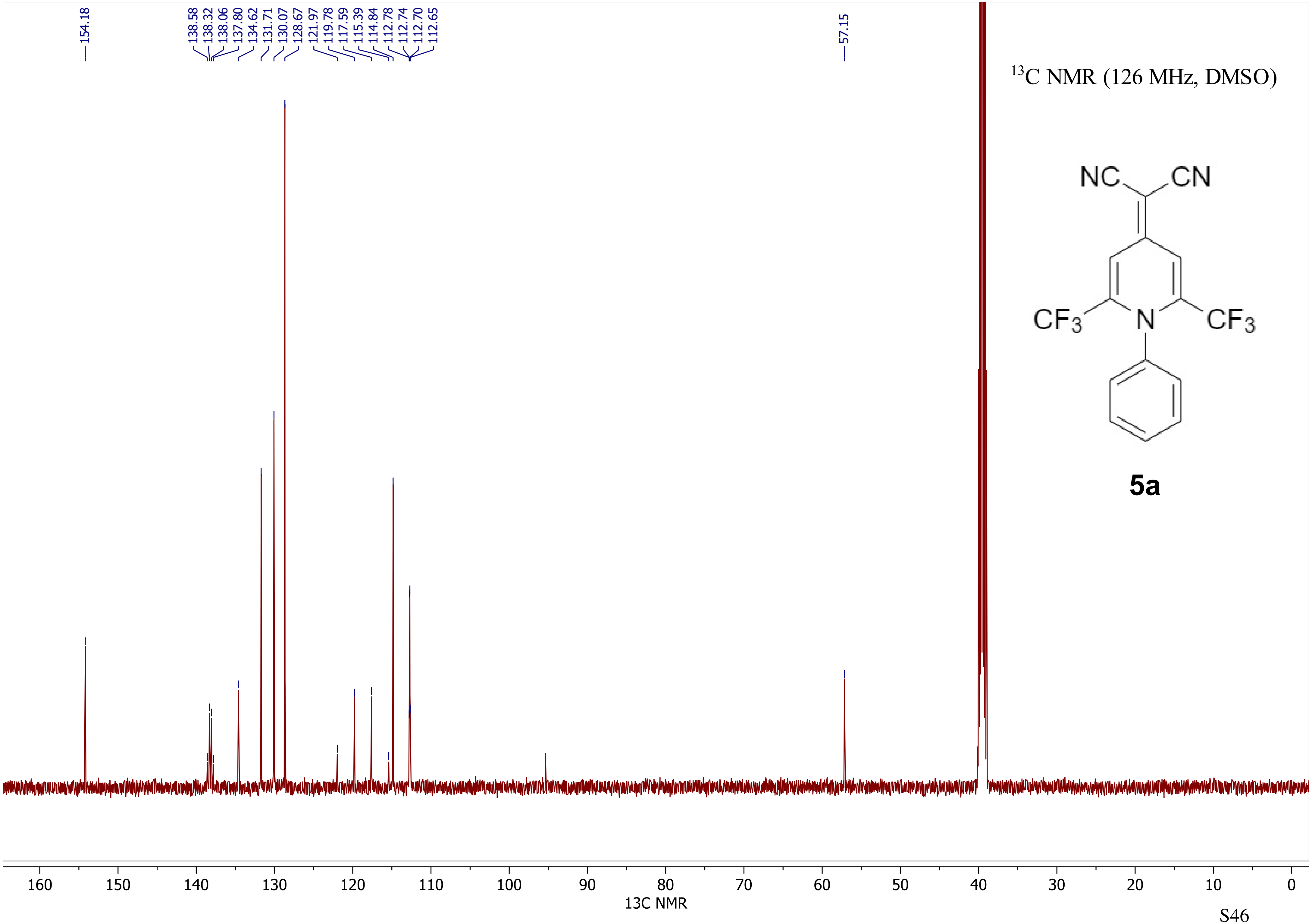
154.18
138.58
138.32
138.06
137.80
134.62
131.71
130.07
128.67
121.97
119.78
117.59
115.39
114.84
112.78
112.74
112.70
112.65
57.15
^{13}C NMR (126 MHz, DMSO)
NC
CN
CF_3
N
CF_3
5a
160
150
140
130
120
110
100
90
80
70
60
50
40
30
20
10
0
13C NMR

^{1}H NMR (500 MHz, DMSO)

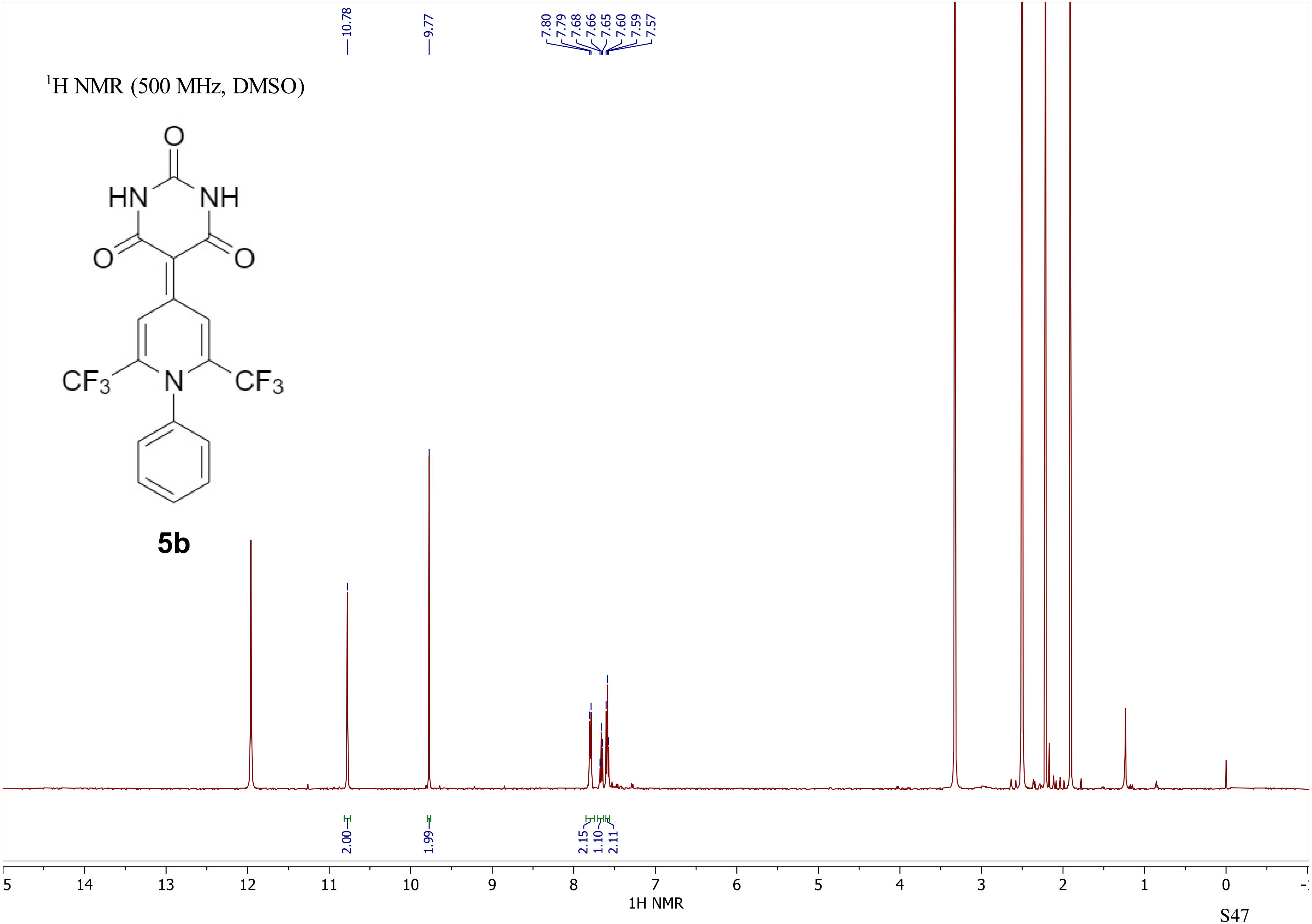

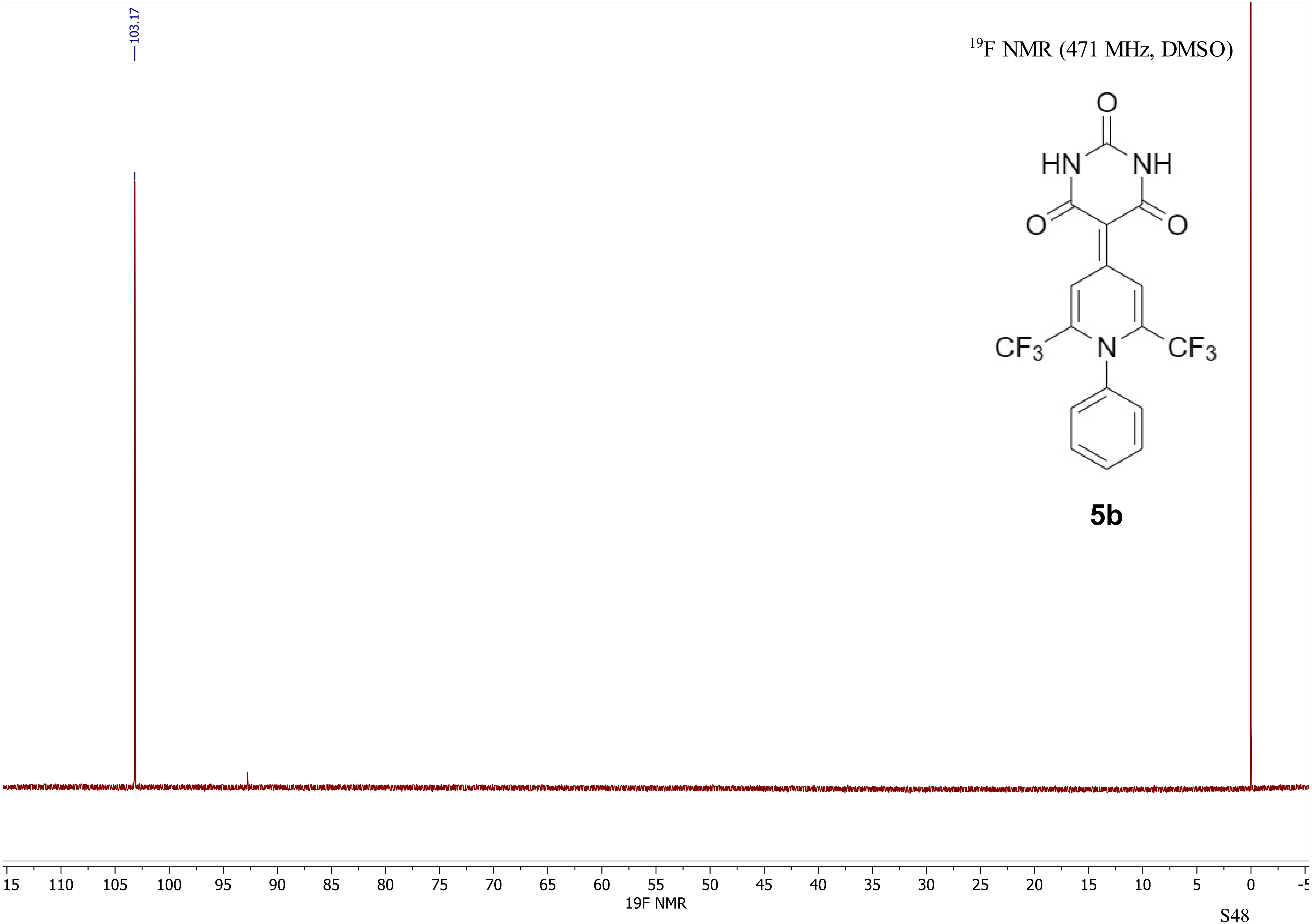
103.17
19F NMR (471 MHz, DMSO)
HN
NH
O
CF3
N
5b
15 110 105 100 95 90 85 80 75 70 65 60 55 50 45 40 35 30 25 20 15 10 5 0
19F NMR

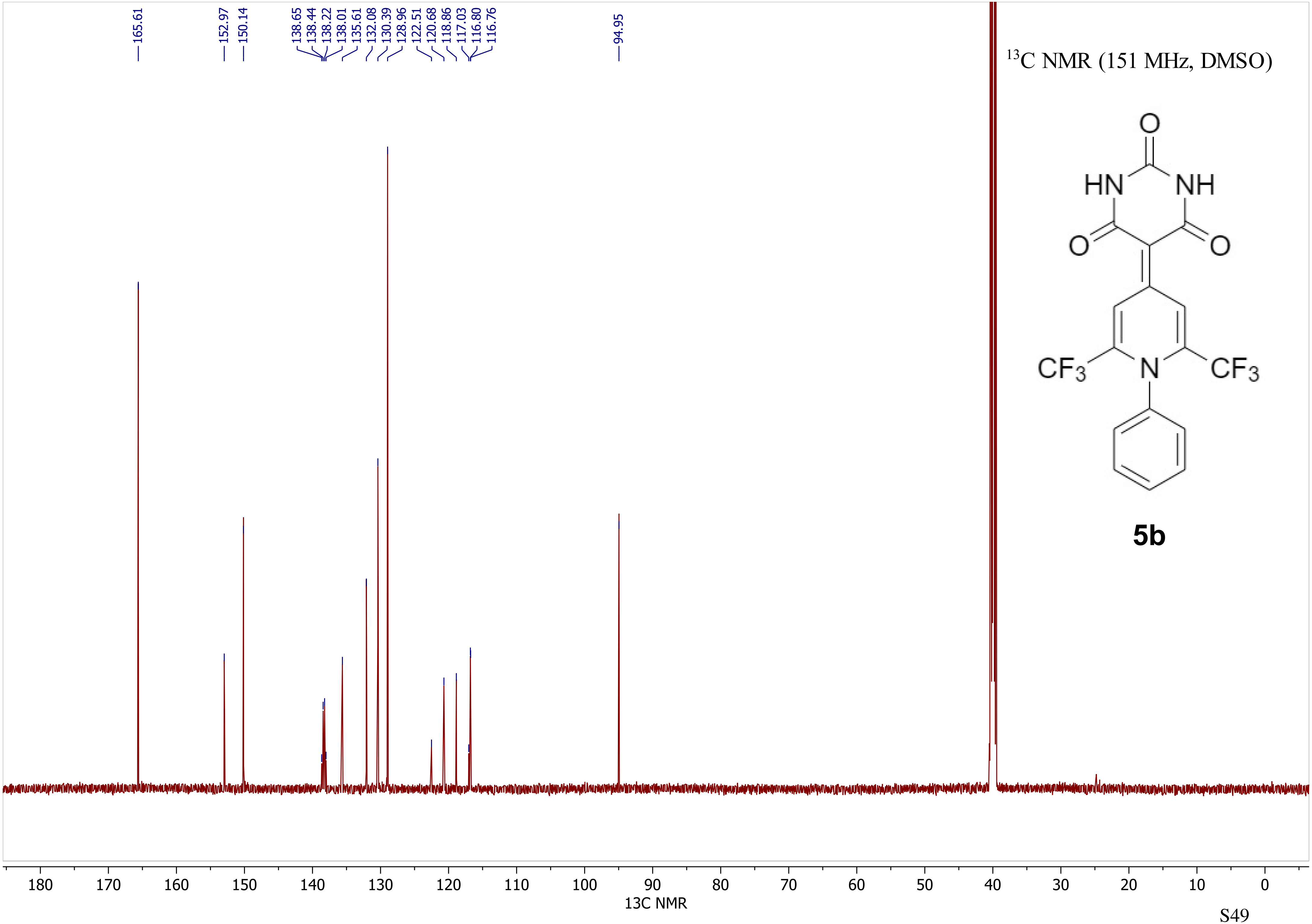
165.61
152.97
150.14
138.65
138.44
138.22
138.01
135.61
132.08
130.39
128.96
122.51
120.68
118.86
117.03
116.80
116.76
94.95
^{13}C NMR (151 MHz, DMSO)
HN
NH
O
O
O
CF3
N
CF3
5b
180 170 160 150 140 130 120 110 100 90 80 70 60 50 40 30 20 10 0
13C NMR

^{1}H NMR (500 MHz, DMSO)

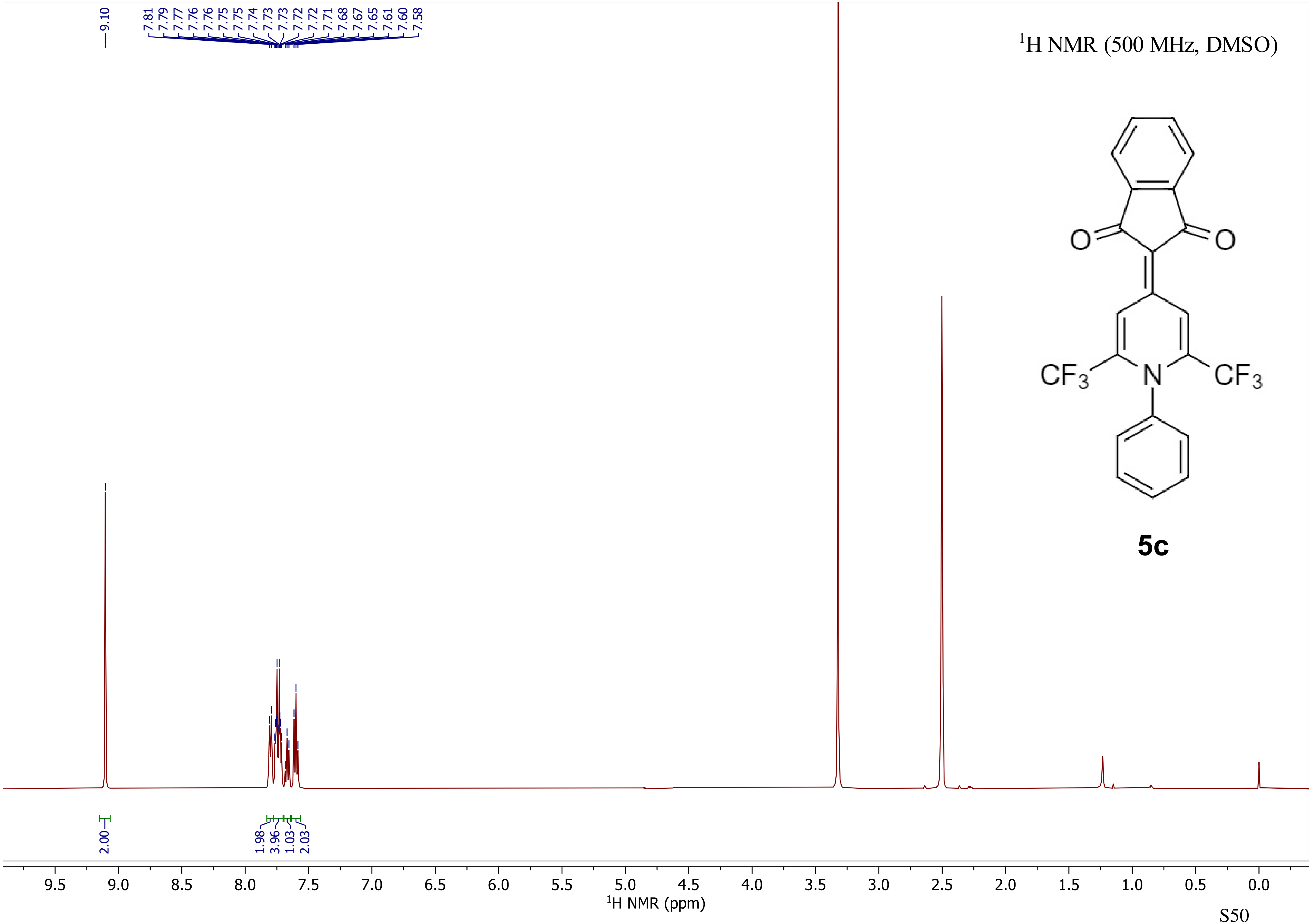

5c

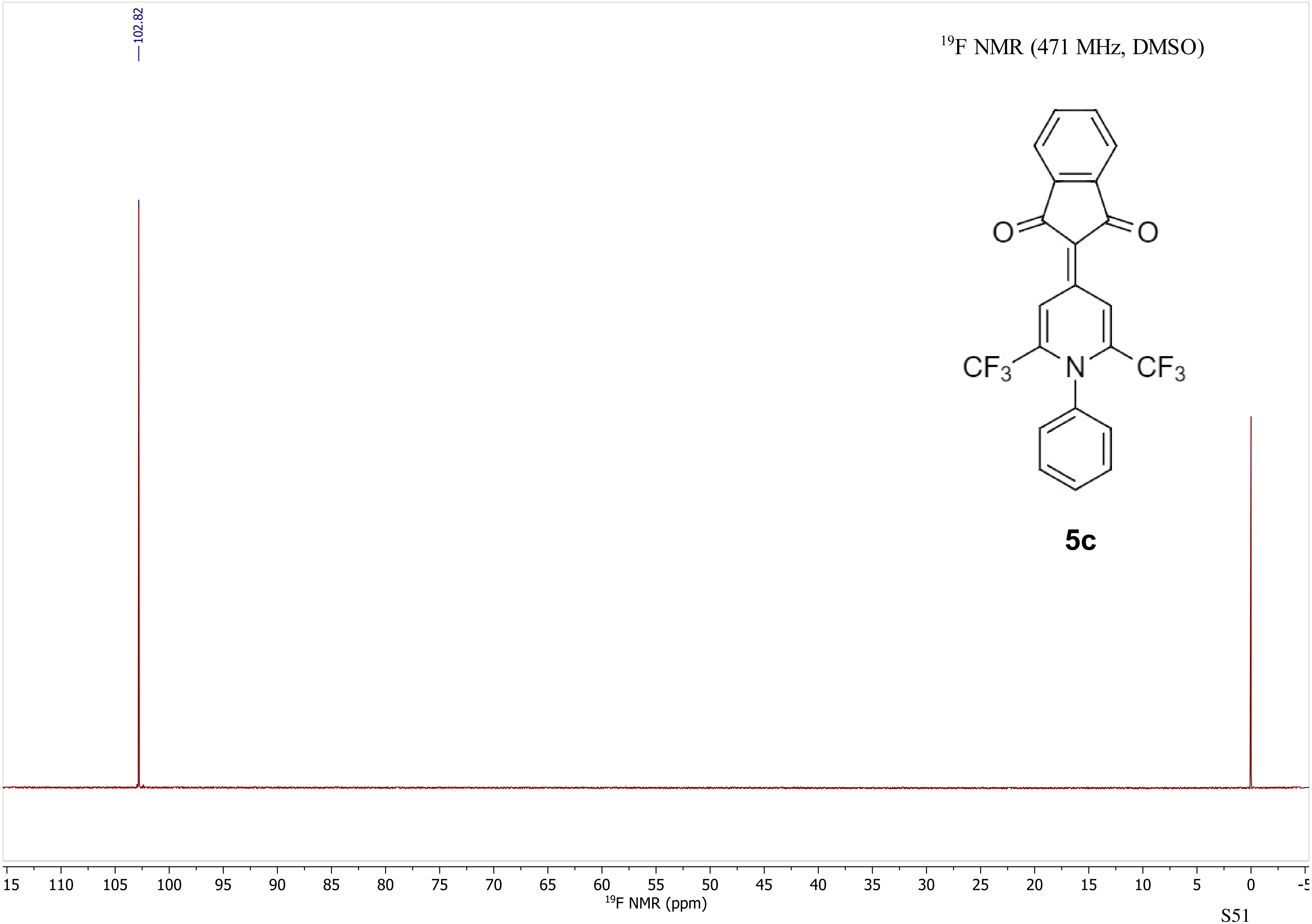
102.82
19F NMR (471 MHz, DMSO)
O
O
CF3
N
CF3
5c
15 110 105 100 95 90 85 80 75 70 65 60 55 50 45 40 35 30 25 20 15 10 5 0
19F NMR (ppm)

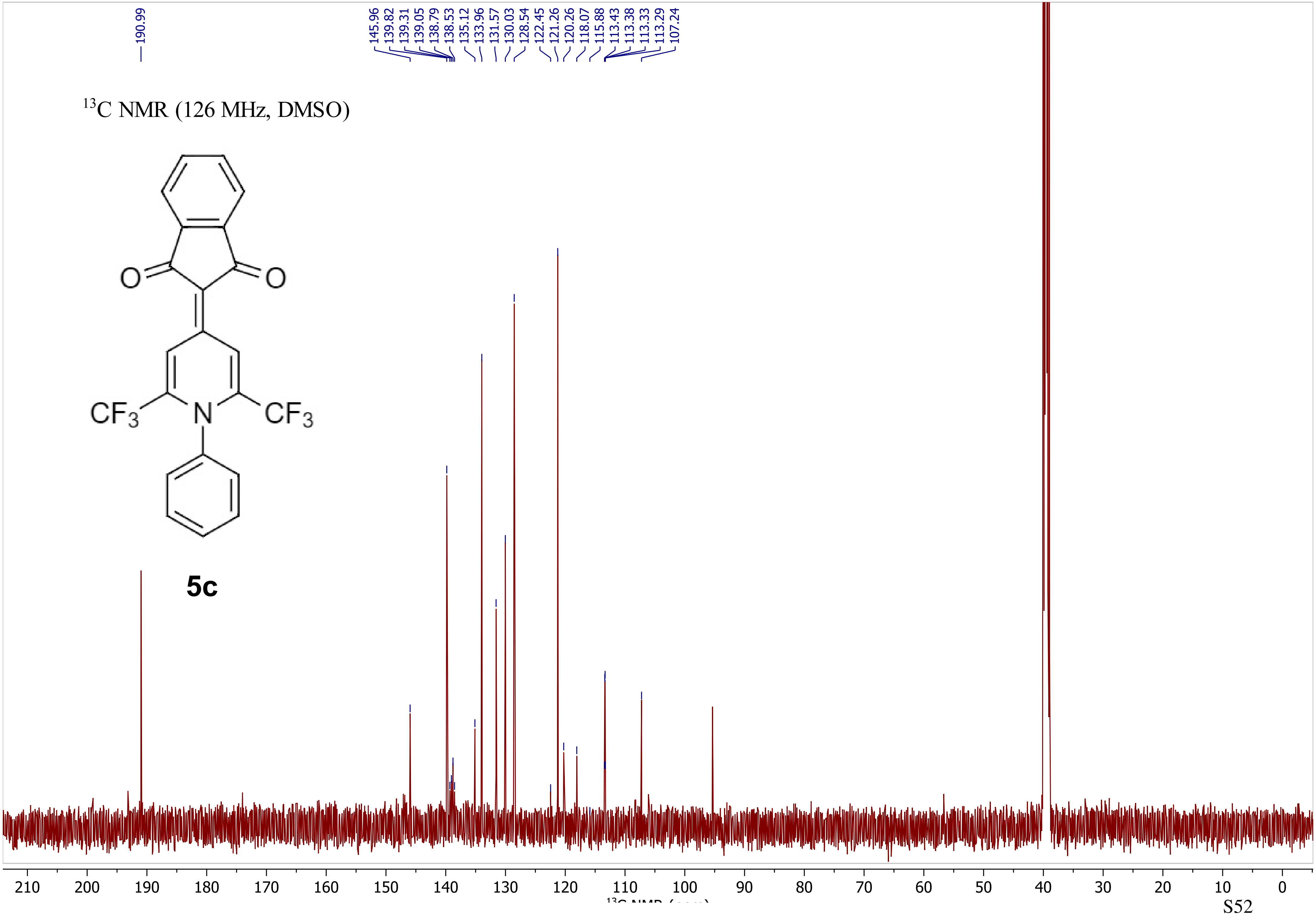

190.99
145.96
139.82
139.31
139.05
138.79
138.53
135.12
133.96
131.57
130.03
128.54
122.45
121.26
120.26
118.07
115.88
113.43
113.38
113.33
113.29
107.24
^{13}C NMR (126 MHz, DMSO)
O
O
CF3
N
CF3
5c
210
200
190
180
170
160
150
140
130
120
110
100
90
80
70
60
50
40
30
20
10
0

^{1}H NMR (500 MHz, DMSO)

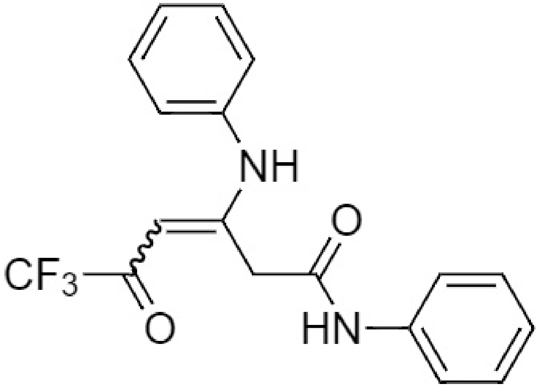

9a

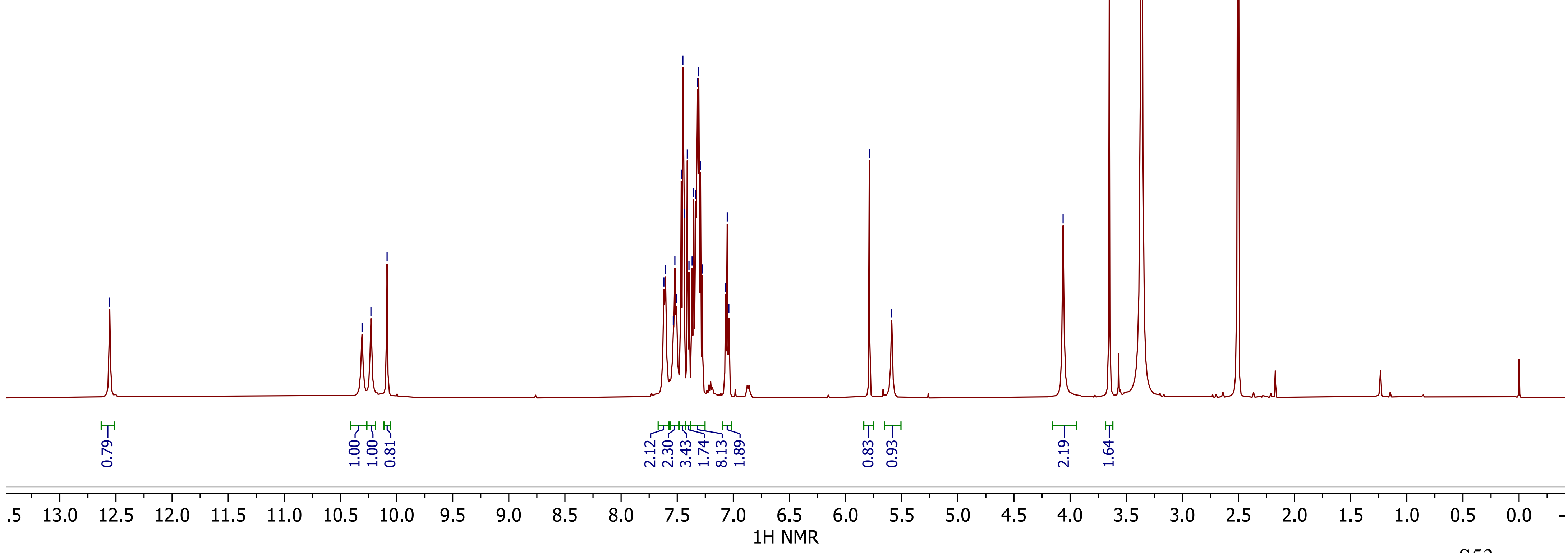

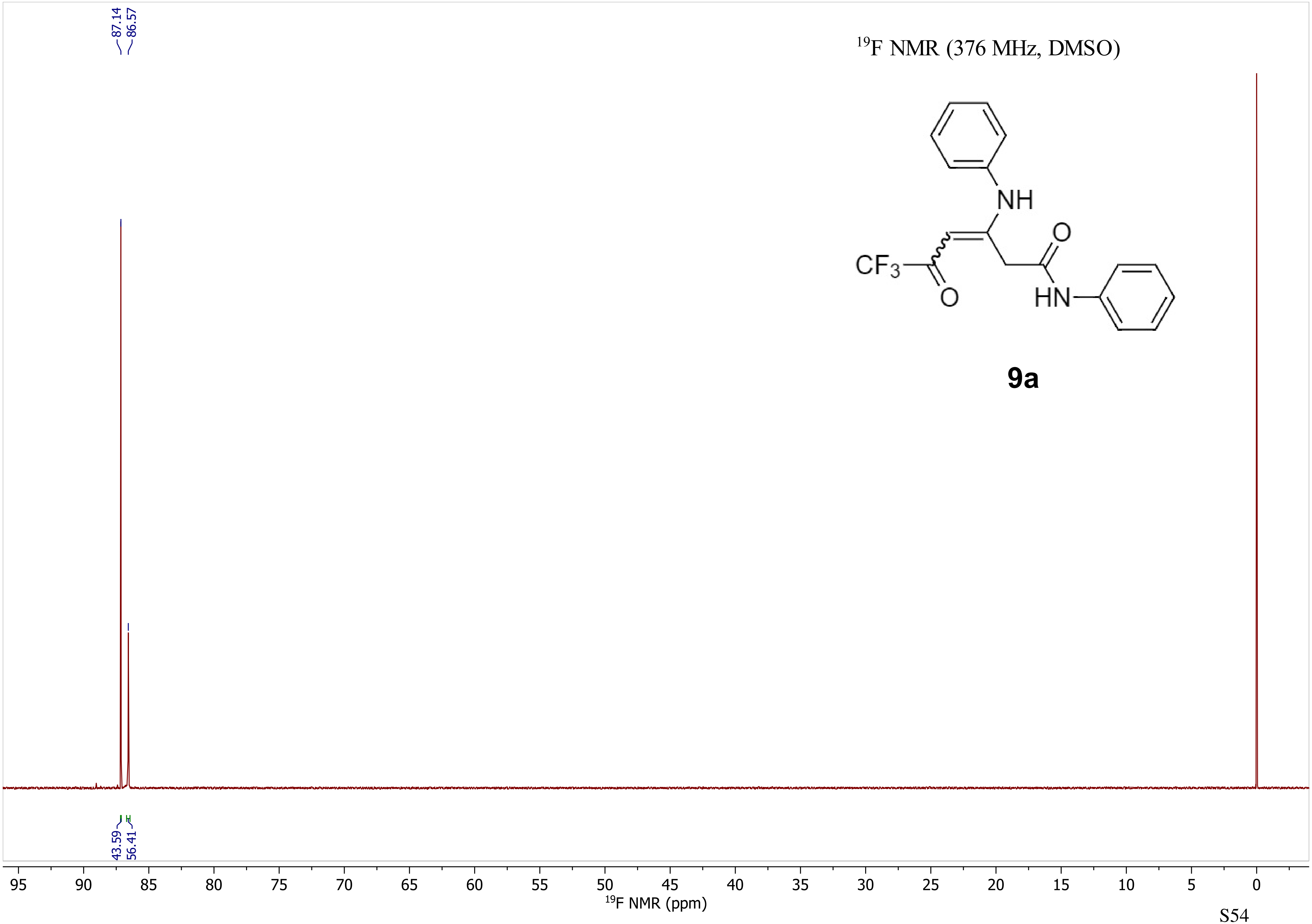

87.14
86.57
19F NMR (376 MHz, DMSO)
NH
O
CF3
O
HN
9a
43.59
56.41
95 90 85 80 75 70 65 60 55 50 45 40 35 30 25 20 15 10 5 0
19F NMR (ppm)

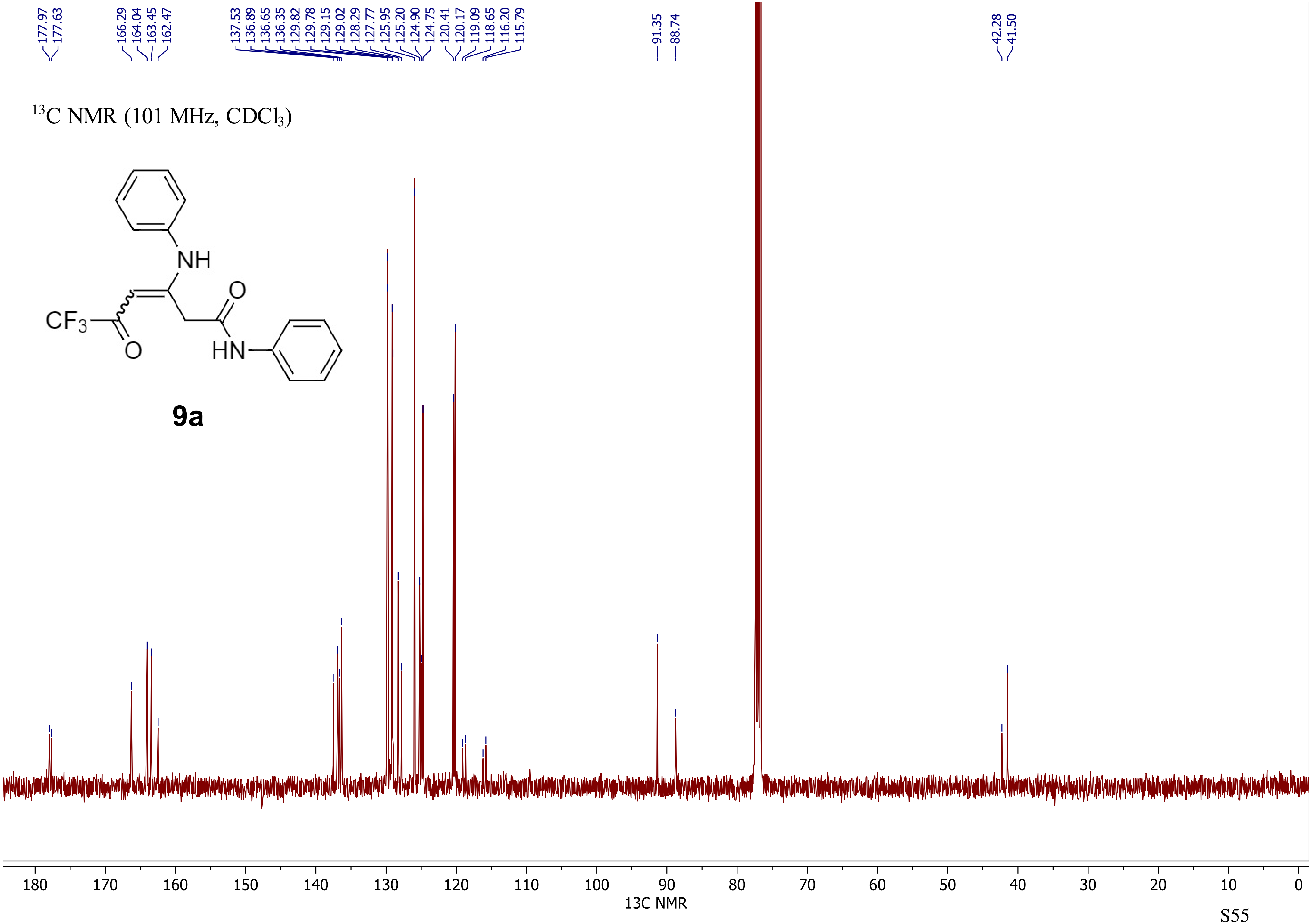
177.97
177.63
166.29
164.04
163.45
162.47
137.53
136.89
136.65
136.35
129.82
129.78
129.15
129.02
128.29
127.77
125.95
125.20
124.90
124.75
120.41
120.17
119.09
118.65
116.20
115.79
91.35
88.74
42.28
41.50
13C NMR (101 MHz, CDCl3)
NH
O
CF3
O
HN
9a
180 170 160 150 140 130 120 110 100 90 80 70 60 50 40 30 20 10 0
13C NMR

^{1}H NMR (400 MHz, CDCl$_3$)

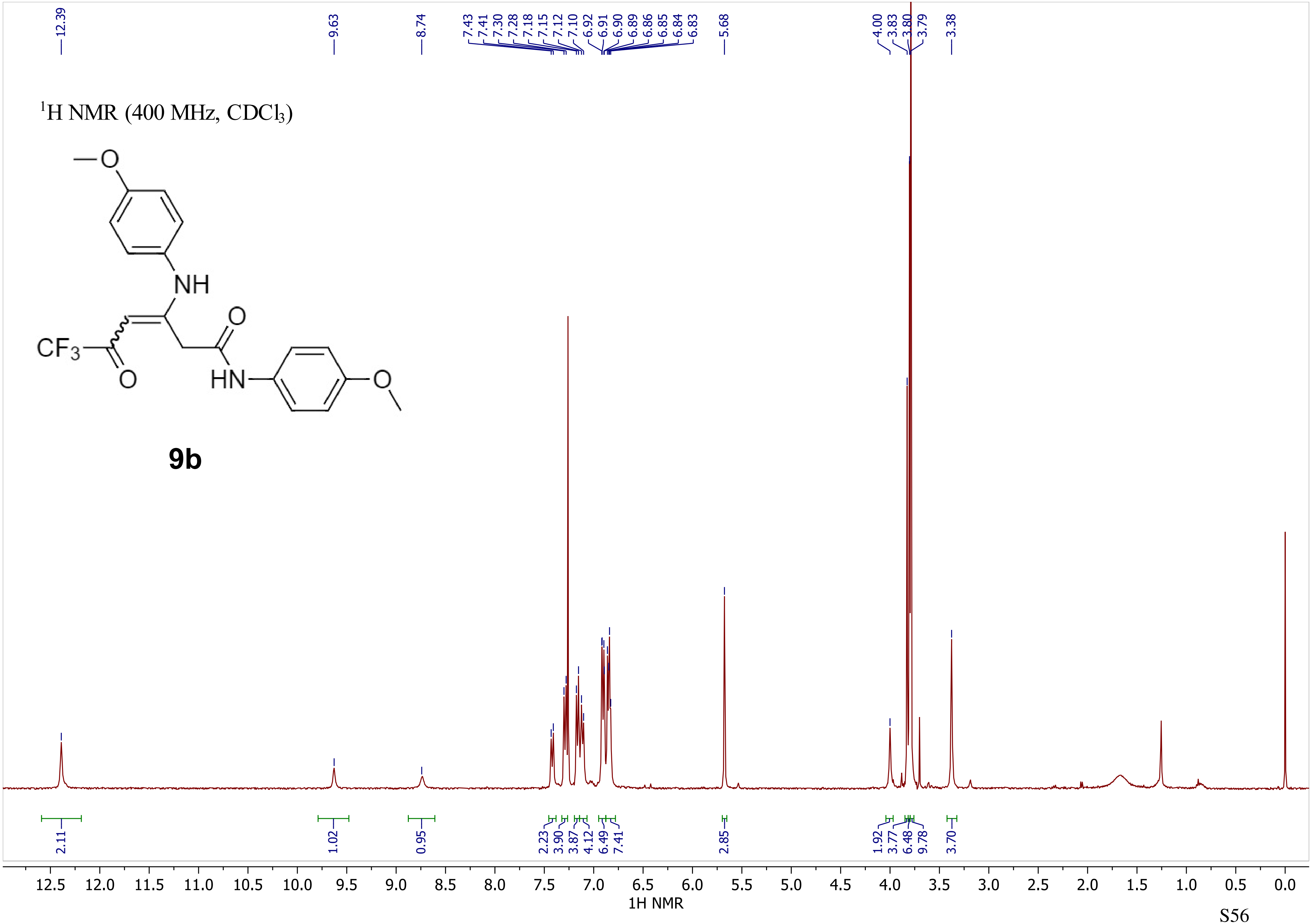

9b

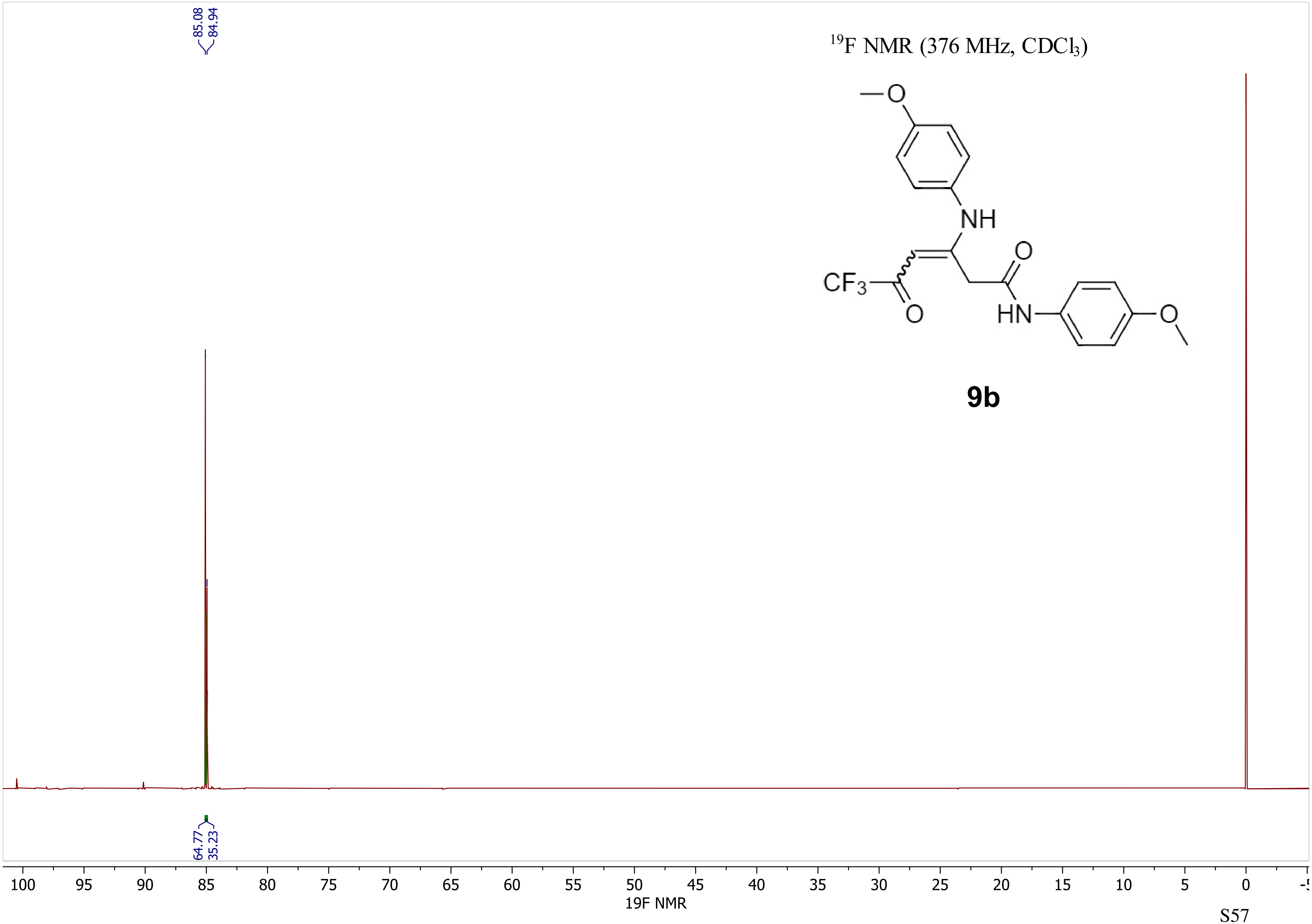
85.08
84.94
19F NMR (376 MHz, CDCl3)
NH
O
CF3
O
HN
O
O
9b
64.77
35.23
100
95
90
85
80
75
70
65
60
55
50
45
40
35
30
25
20
15
10
5
0
19F NMR

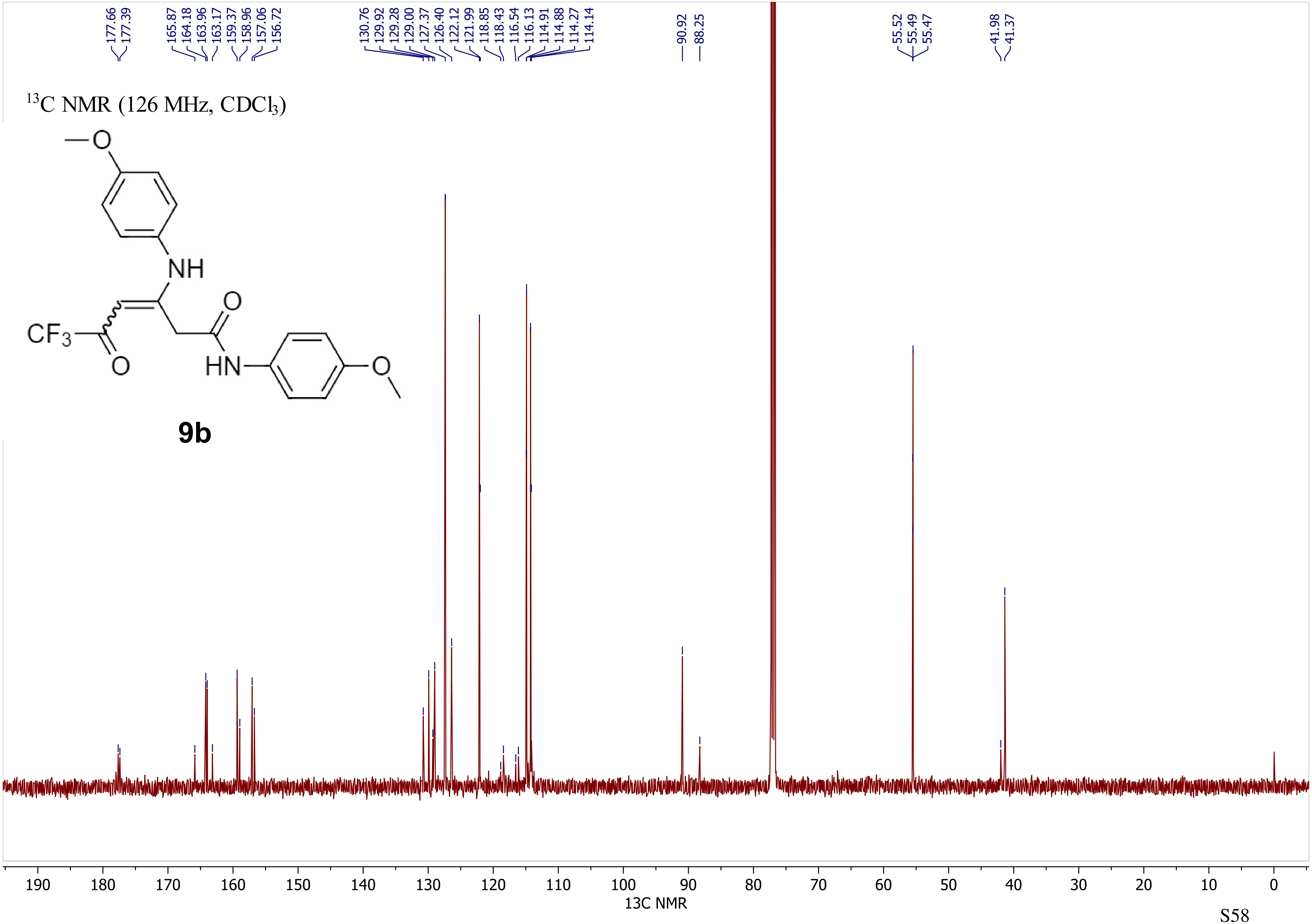
177.66
177.39
165.87
164.18
163.96
163.17
159.37
158.96
157.06
156.72
130.76
129.92
129.28
129.00
127.37
126.40
122.12
121.99
118.85
118.43
116.54
116.13
114.91
114.88
114.27
114.14
90.92
88.25
55.52
55.49
55.47
41.98
41.37
^{13}C NMR (126 MHz, $CDCl_3$)
NH
O
CF_3
O
HN
O
O
9b
190 180 170 160 150 140 130 120 110 100 90 80 70 60 50 40 30 20 10 0
13C NMR

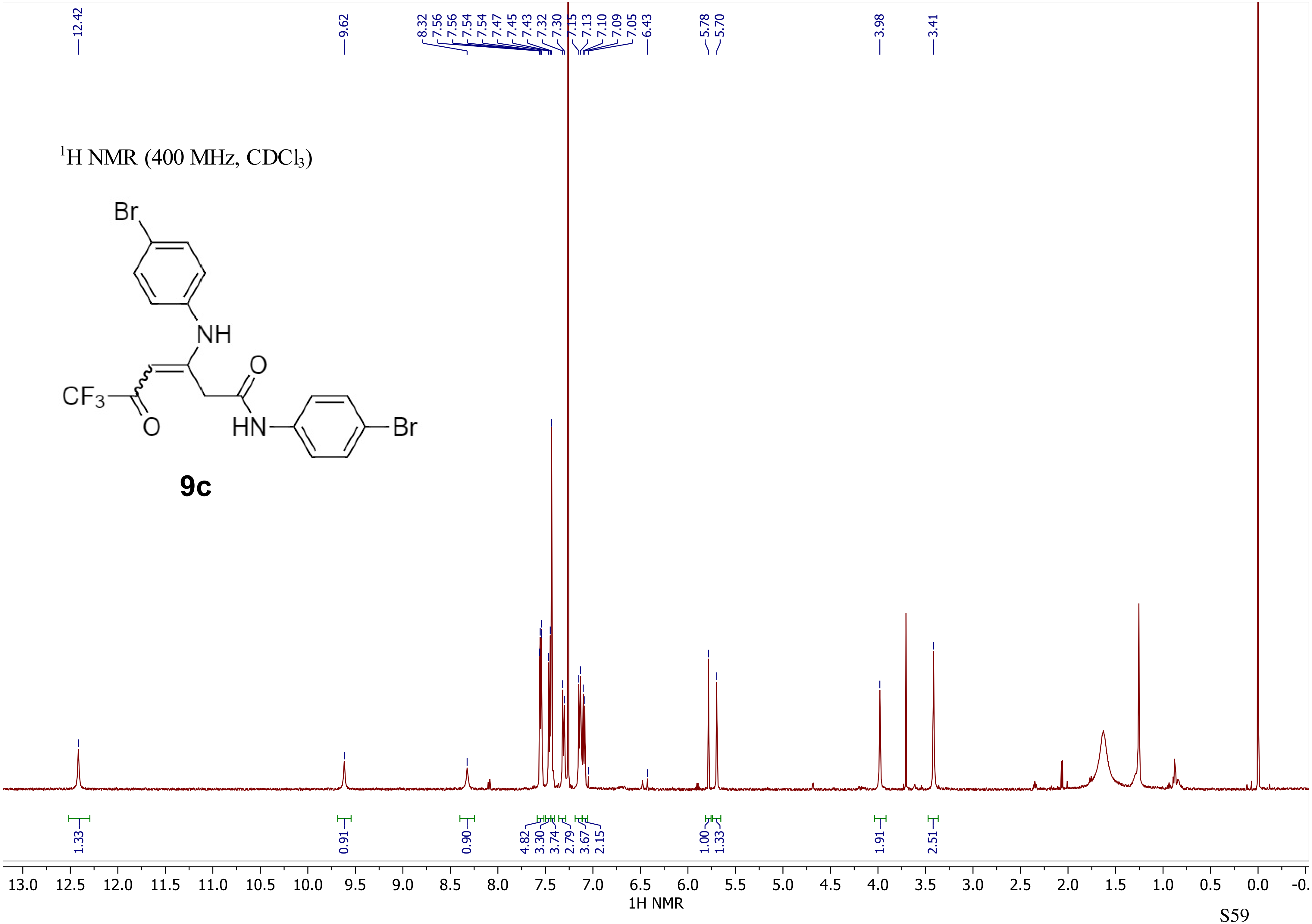

12.42
9.62
8.32
7.56
7.56
7.54
7.54
7.47
7.45
7.43
7.32
7.30
7.15
7.13
7.10
7.09
7.05
6.43
5.78
5.70
3.98
3.41
^{1}H NMR (400 MHz, CDCl$_3$)
Br
NH
O
CF$_3$
O
HN
Br
9c
1.33
0.91
0.90
4.82
3.30
3.74
2.79
3.67
2.15
1.00
1.33
1.91
2.51
13.0 12.5 12.0 11.5 11.0 10.5 10.0 9.5 9.0 8.5 8.0 7.5 7.0 6.5 6.0 5.5 5.0 4.5 4.0 3.5 3.0 2.5 2.0 1.5 1.0 0.5 0.0
1H NMR

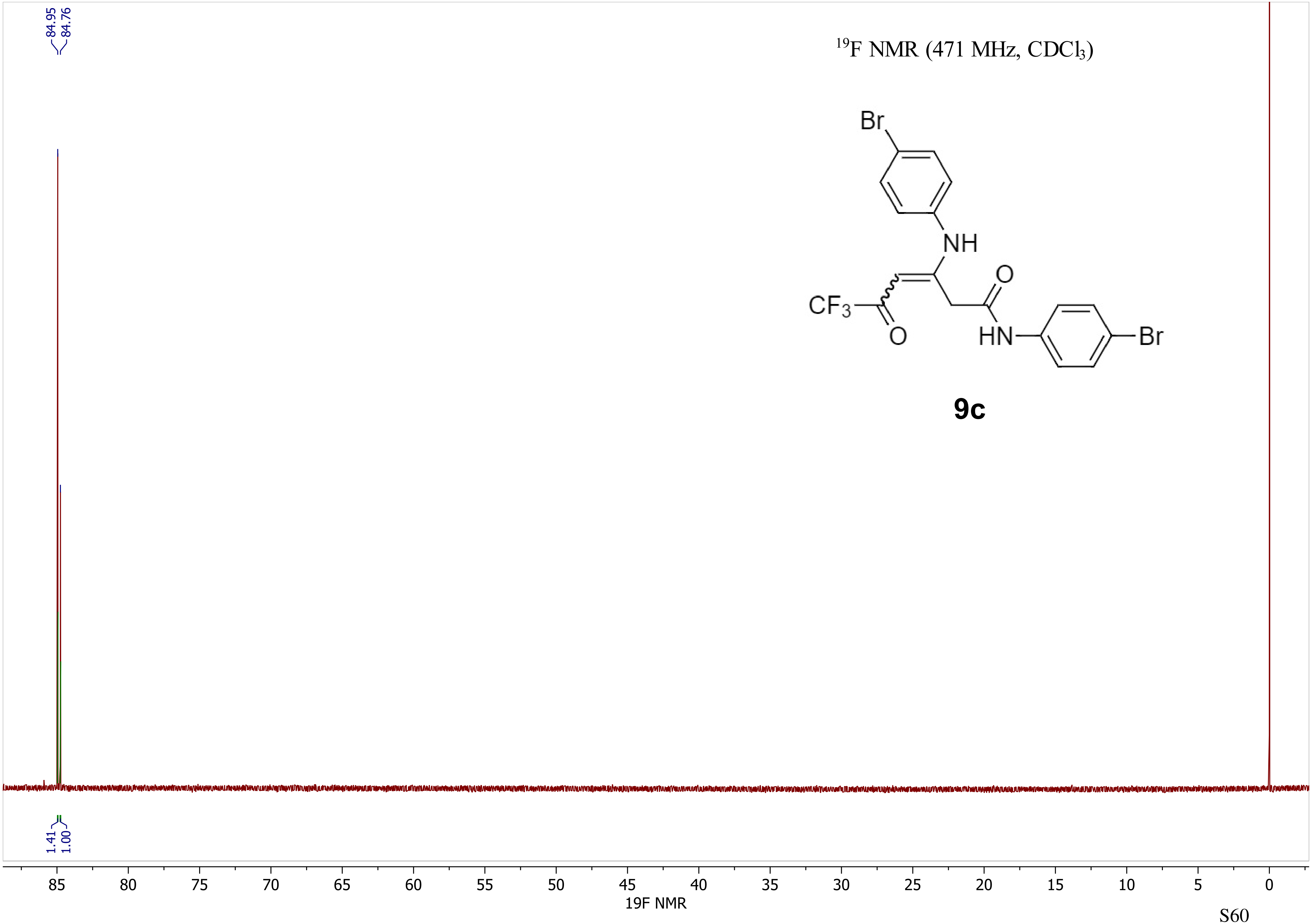
84.95
84.76
^{19}F NMR (471 MHz, CDCl$_3$)
Br
NH
O
CF3
O
HN
Br
9c
1.41
1.00
85
80
75
70
65
60
55
50
45
40
35
30
25
20
15
10
5
0
19F NMR

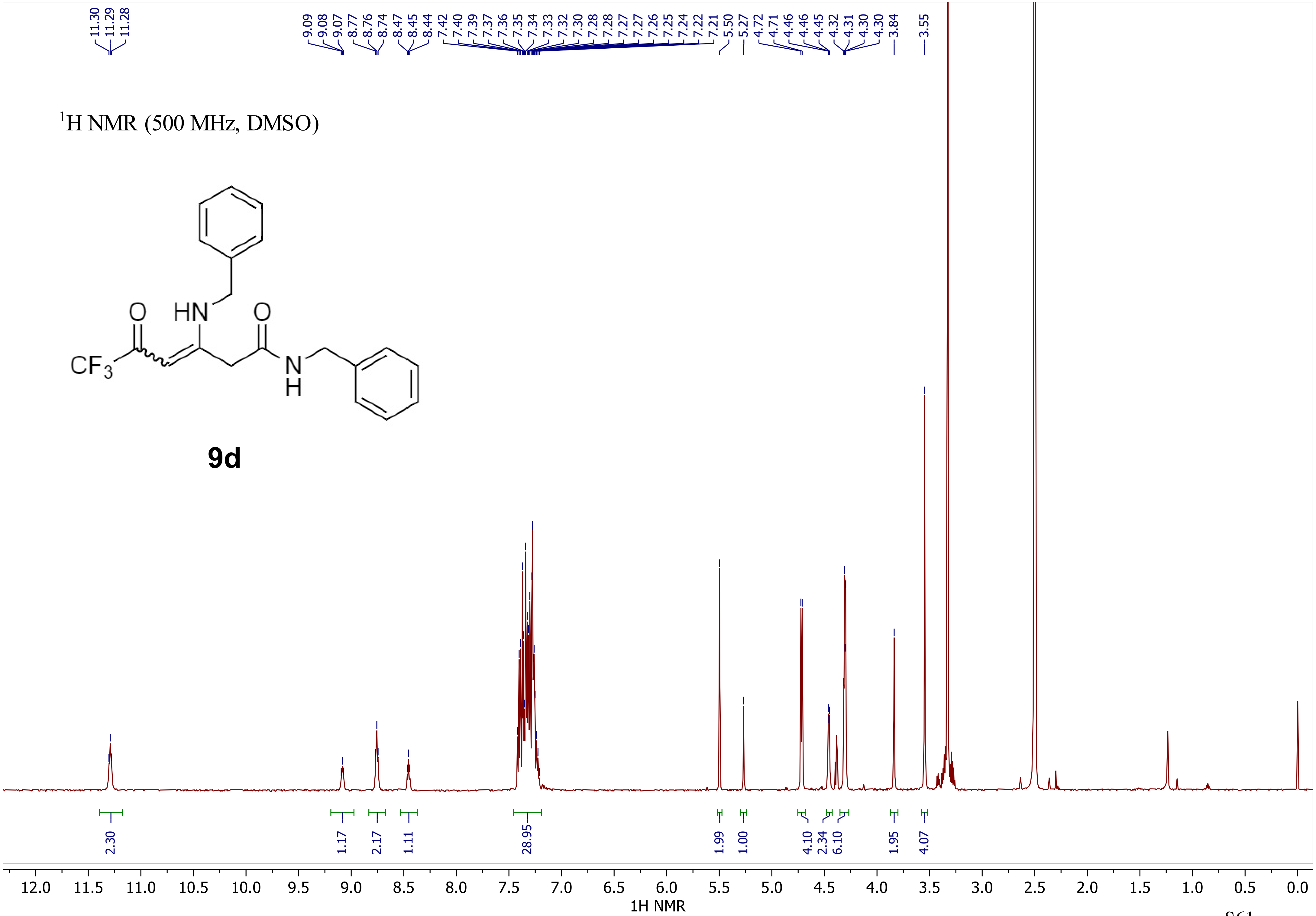

1H NMR (500 MHz, DMSO)
9d
11.30 11.29 11.28
9.09 9.08 9.07 8.77 8.76 8.74 8.47 8.45 8.44 7.42 7.40 7.39 7.37 7.36 7.35 7.34 7.33 7.32 7.30 7.28 7.28 7.27 7.27 7.26 7.25 7.24 7.22 7.21 5.50 5.27 4.72 4.71 4.46 4.46 4.45 4.32 4.31 4.30 4.30 3.84 3.55
2.30 1.17 2.17 1.11 28.95 1.99 1.00 4.10 2.34 6.10 1.95 4.07
12.0 11.5 11.0 10.5 10.0 9.5 9.0 8.5 8.0 7.5 7.0 6.5 6.0 5.5 5.0 4.5 4.0 3.5 3.0 2.5 2.0 1.5 1.0 0.5 0.0
1H NMR

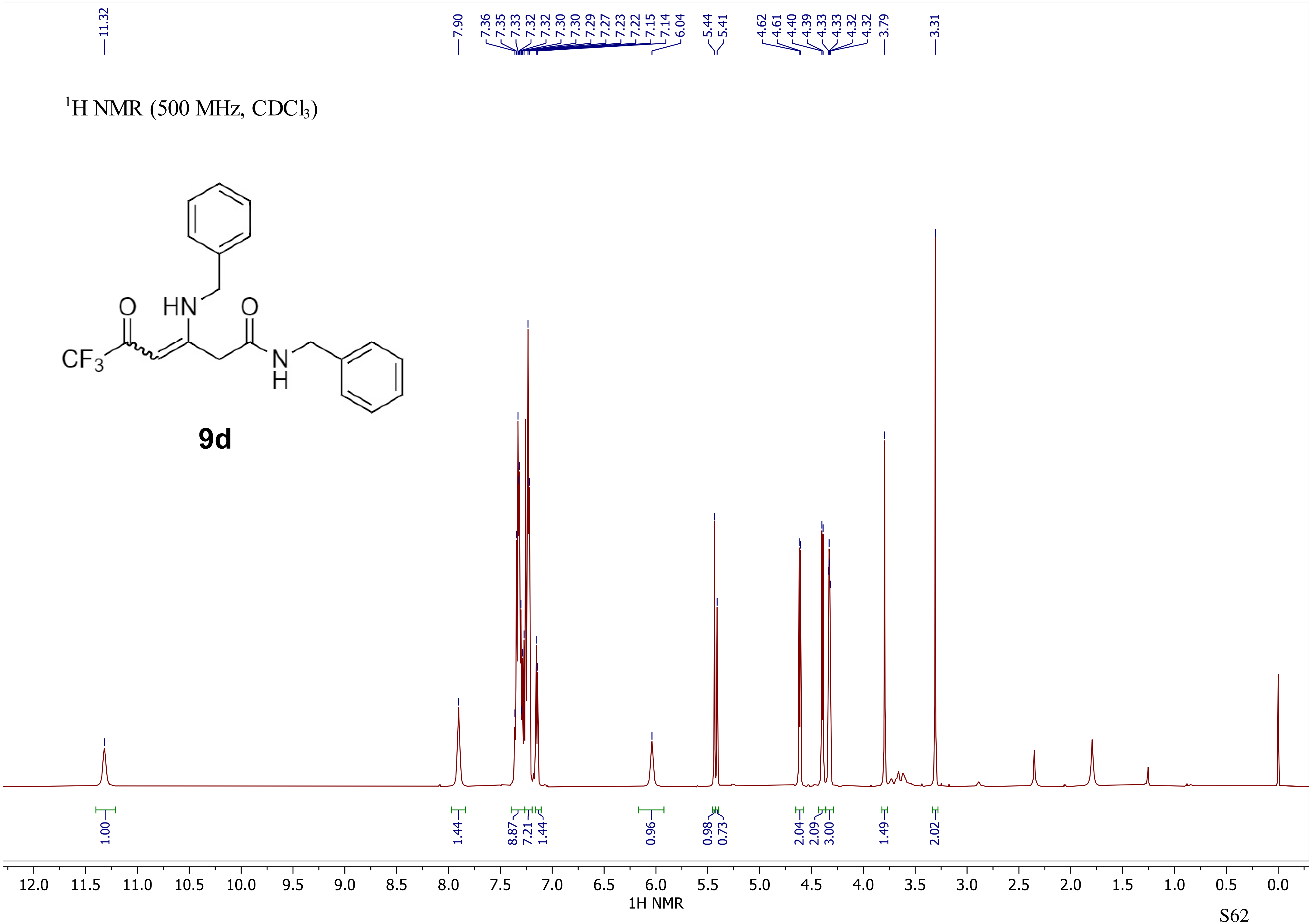

^{1}H NMR (500 MHz, CDCl$_3$)
9d
11.32
7.90
7.36
7.35
7.33
7.32
7.32
7.30
7.30
7.29
7.27
7.23
7.22
7.15
7.14
6.04
5.44
5.41
4.62
4.61
4.40
4.39
4.33
4.33
4.32
4.32
3.79
3.31
1.00
1.44
8.87
7.21
1.44
0.96
0.98
0.73
2.04
2.09
3.00
1.49
2.02
12.0
11.5
11.0
10.5
10.0
9.5
9.0
8.5
8.0
7.5
7.0
6.5
6.0
5.5
5.0
4.5
4.0
3.5
3.0
2.5
2.0
1.5
1.0
0.5
0.0
1H NMR

^{19}F NMR (471 MHz, $CDCl_3$)

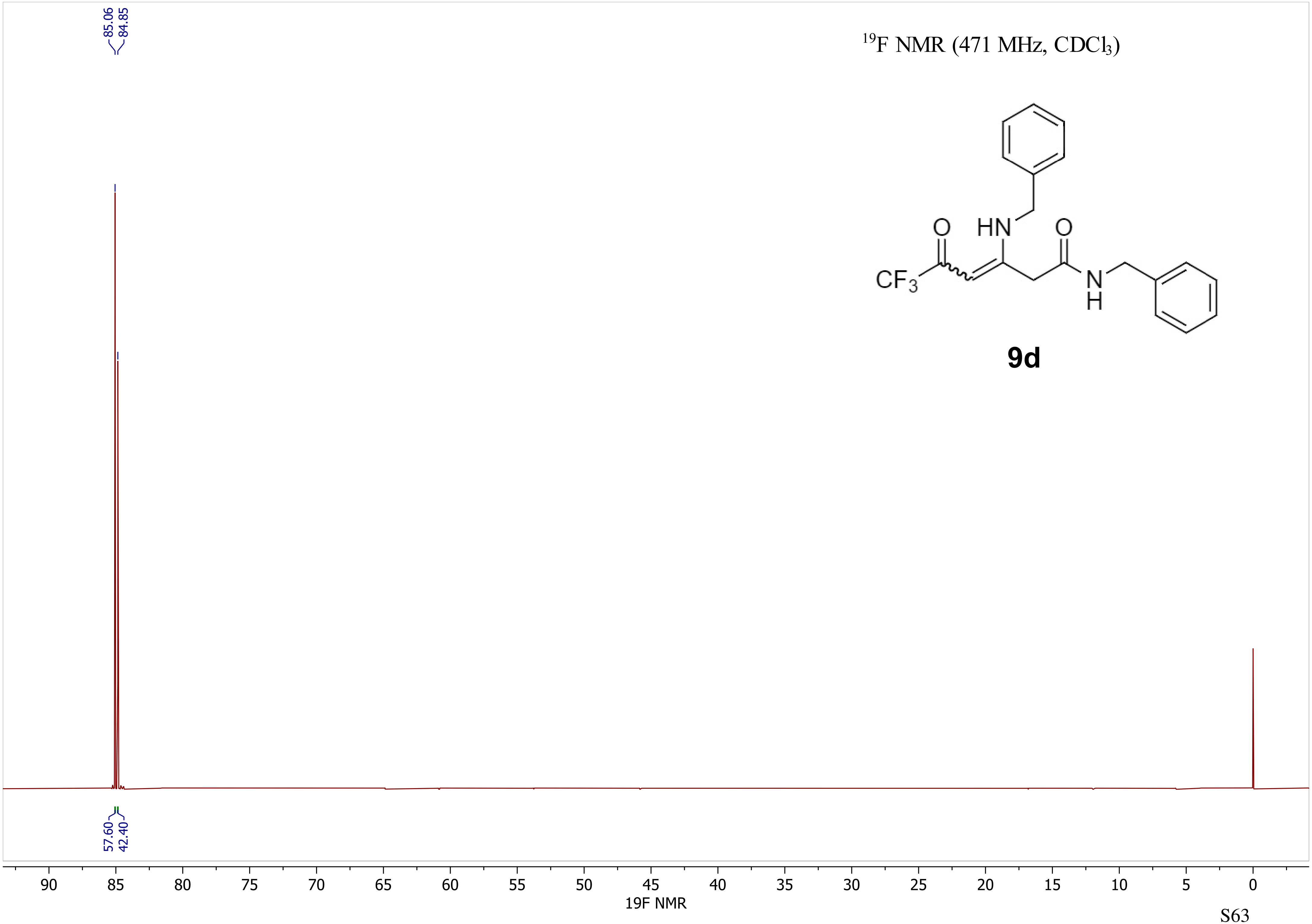

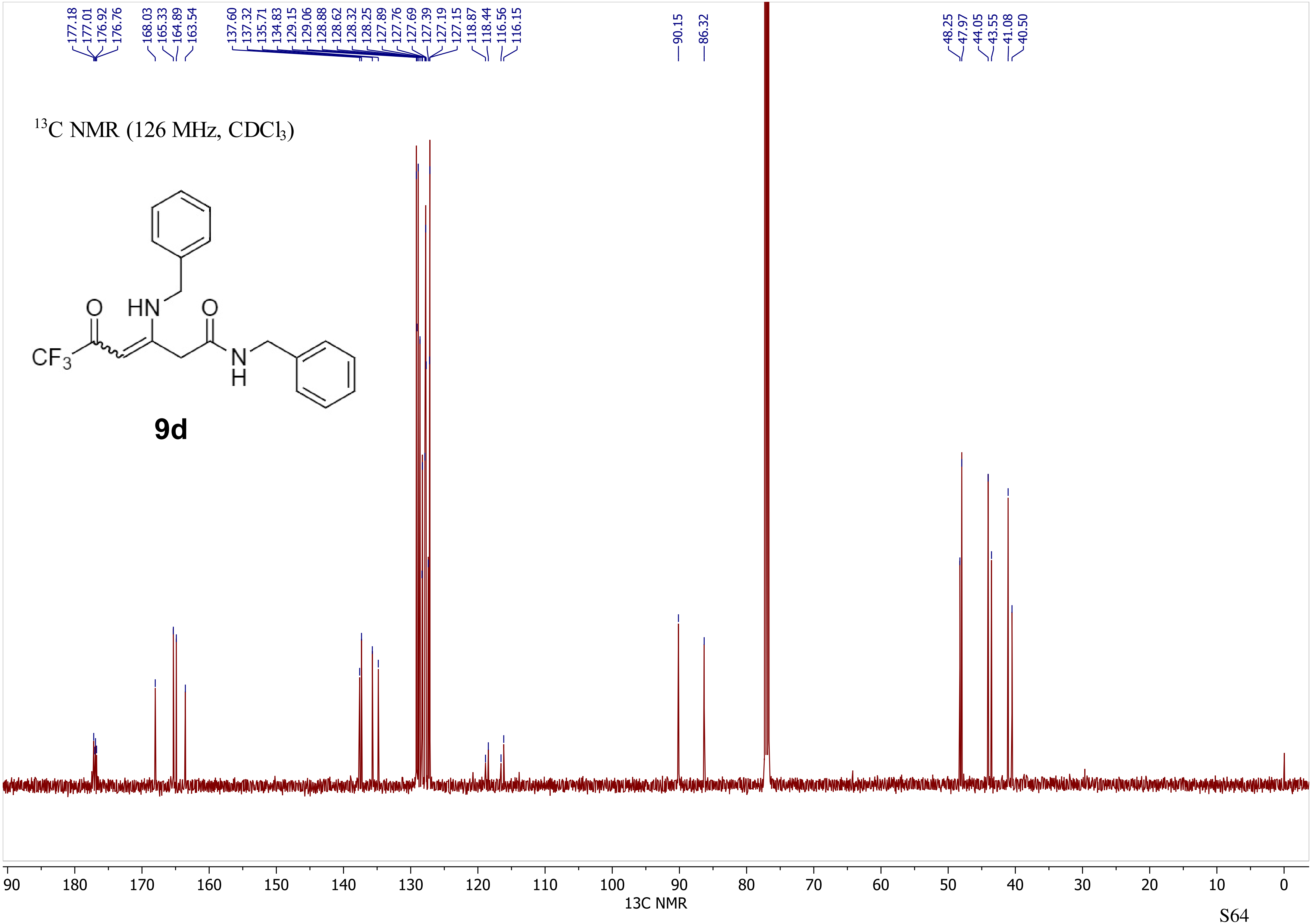

177.18
177.01
176.92
176.76
168.03
165.33
164.89
163.54
137.60
137.32
135.71
134.83
129.15
129.06
128.88
128.62
128.32
128.25
127.89
127.76
127.69
127.39
127.19
127.15
118.87
118.44
116.56
116.15
90.15
86.32
48.25
47.97
44.05
43.55
41.08
40.50
^{13}C NMR (126 MHz, $CDCl_3$)
O
HN
O
CF_3
N
H
9d
90 180 170 160 150 140 130 120 110 100 90 80 70 60 50 40 30 20 10 0
13C NMR

^{1}H NMR (500 MHz, $CDCl_3$)

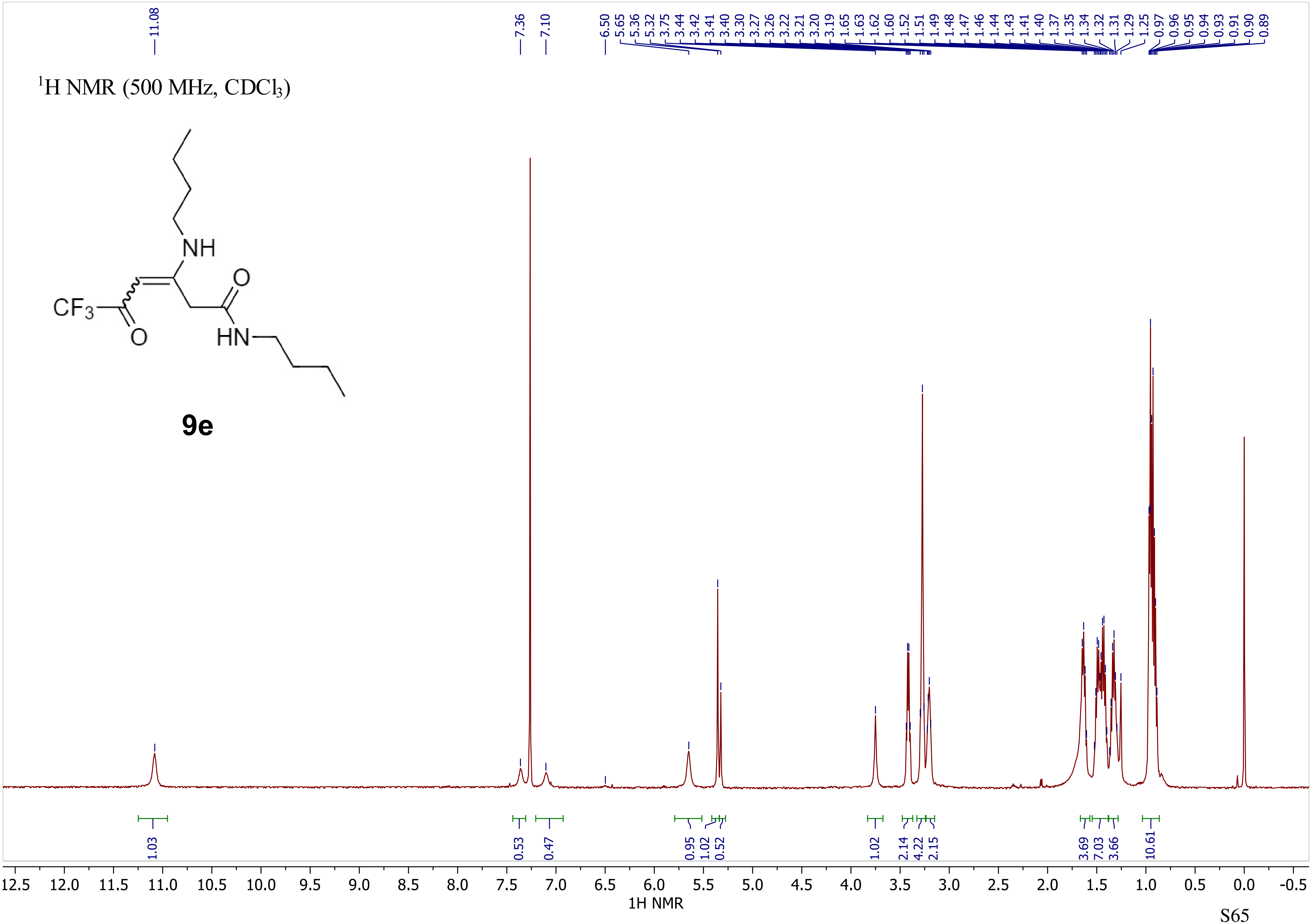

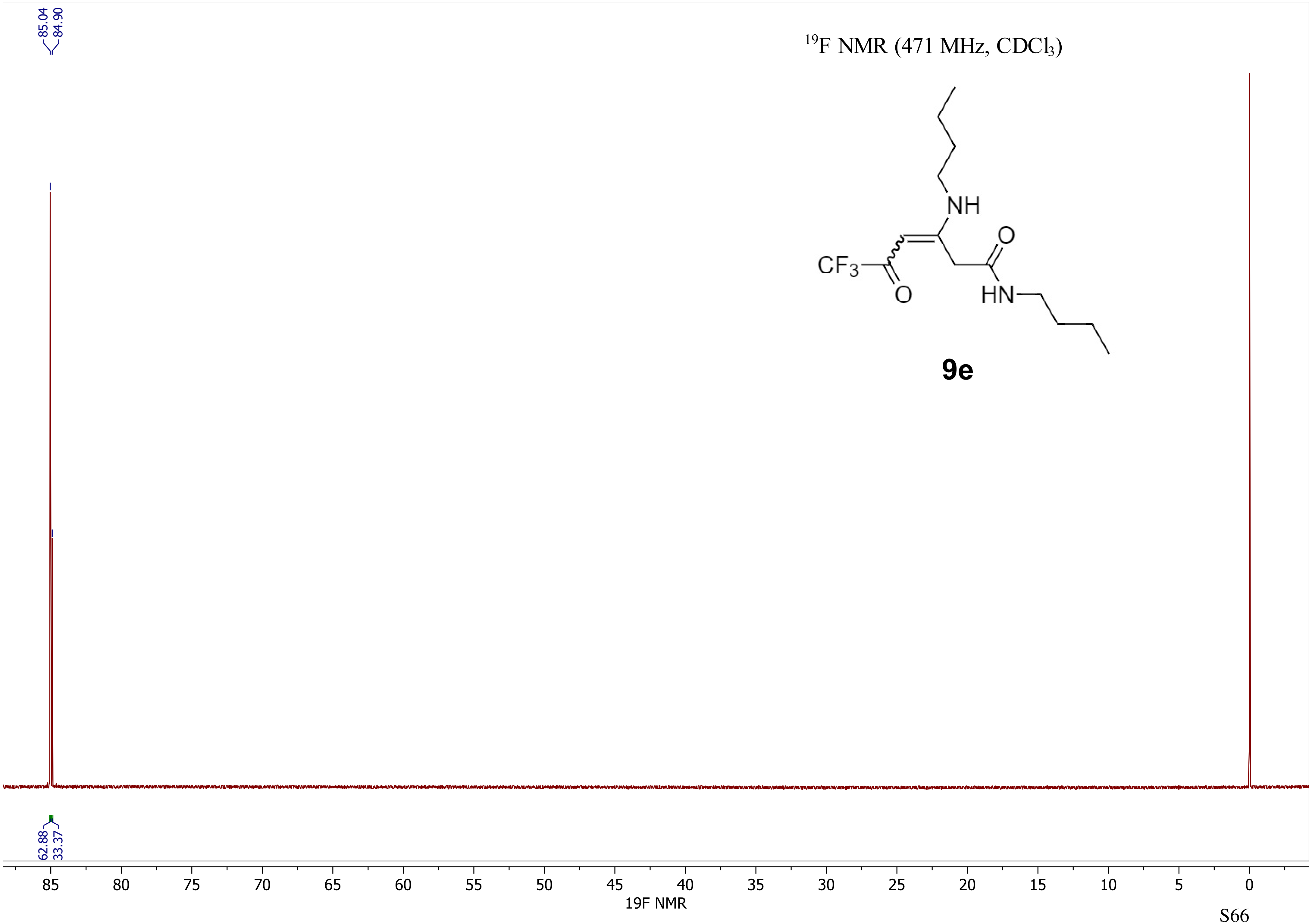

85.04
84.90
19F NMR (471 MHz, CDCl3)
NH
O
CF3
O
HN
9e
62.88
33.37
85
80
75
70
65
60
55
50
45
40
35
30
25
20
15
10
5
0
19F NMR

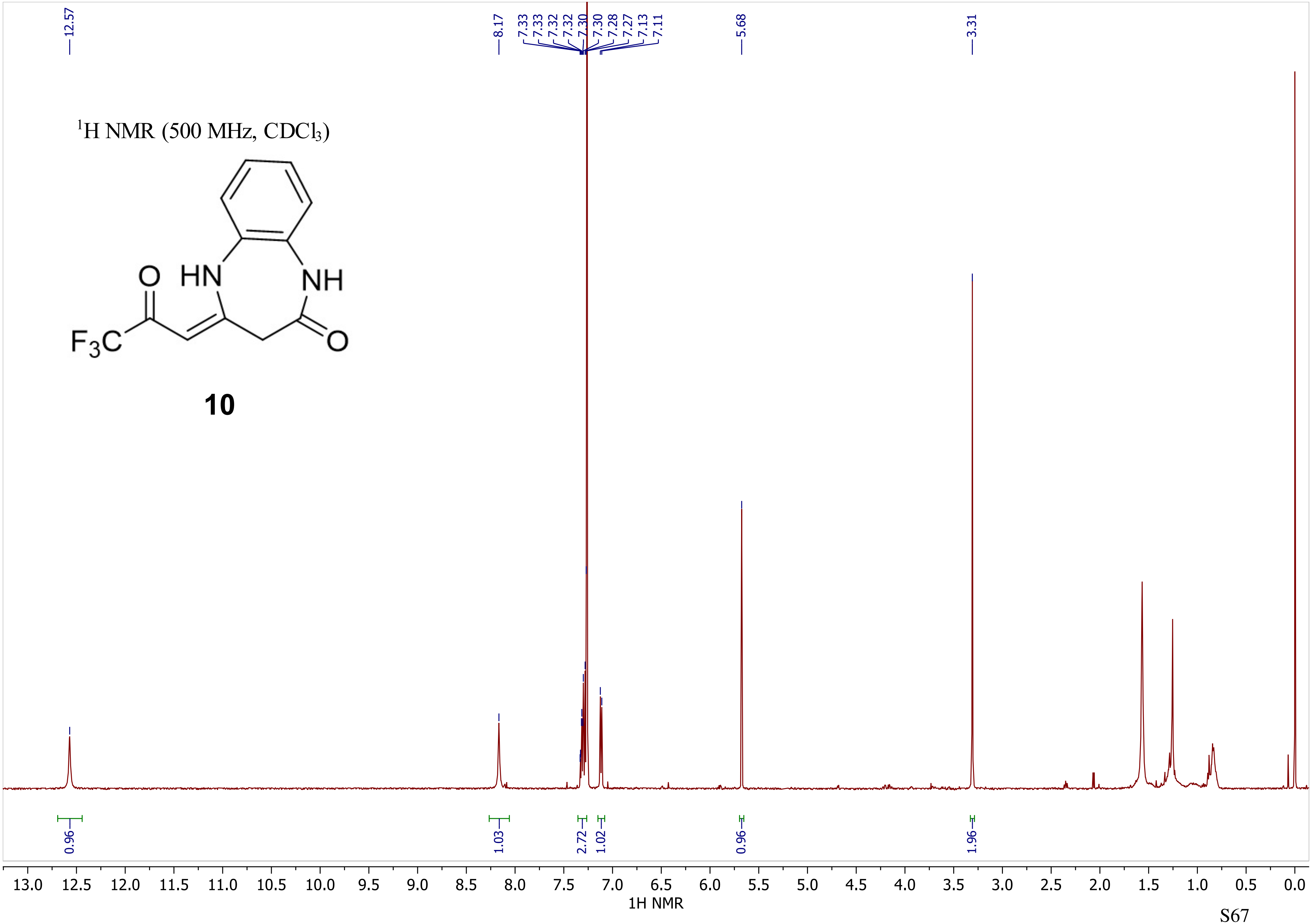

12.57
8.17
7.33
7.33
7.32
7.32
7.30
7.30
7.28
7.27
7.13
7.11
5.68
3.31
^{1}H NMR (500 MHz, $CDCl_3$)
O
HN
NH
F_3C
O
10
0.96
1.03
2.72
1.02
0.96
1.96
13.0
12.5
12.0
11.5
11.0
10.5
10.0
9.5
9.0
8.5
8.0
7.5
7.0
6.5
6.0
5.5
5.0
4.5
4.0
3.5
3.0
2.5
2.0
1.5
1.0
0.5
0.0
1H NMR

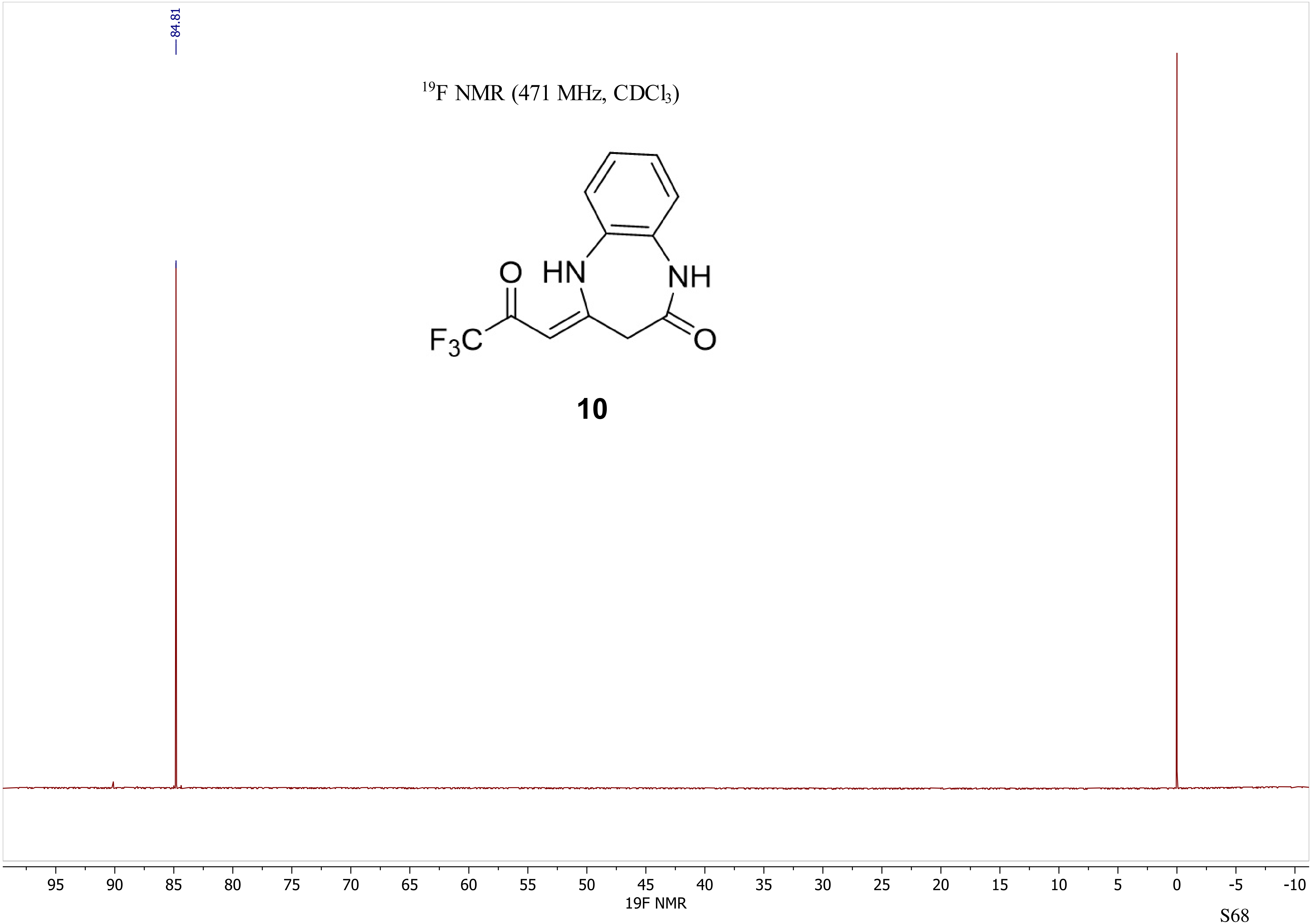
84.81
19F NMR (471 MHz, CDCl3)
O HN NH
F3C O
10
95 90 85 80 75 70 65 60 55 50 45 40 35 30 25 20 15 10 5 0 -5 -10
19F NMR

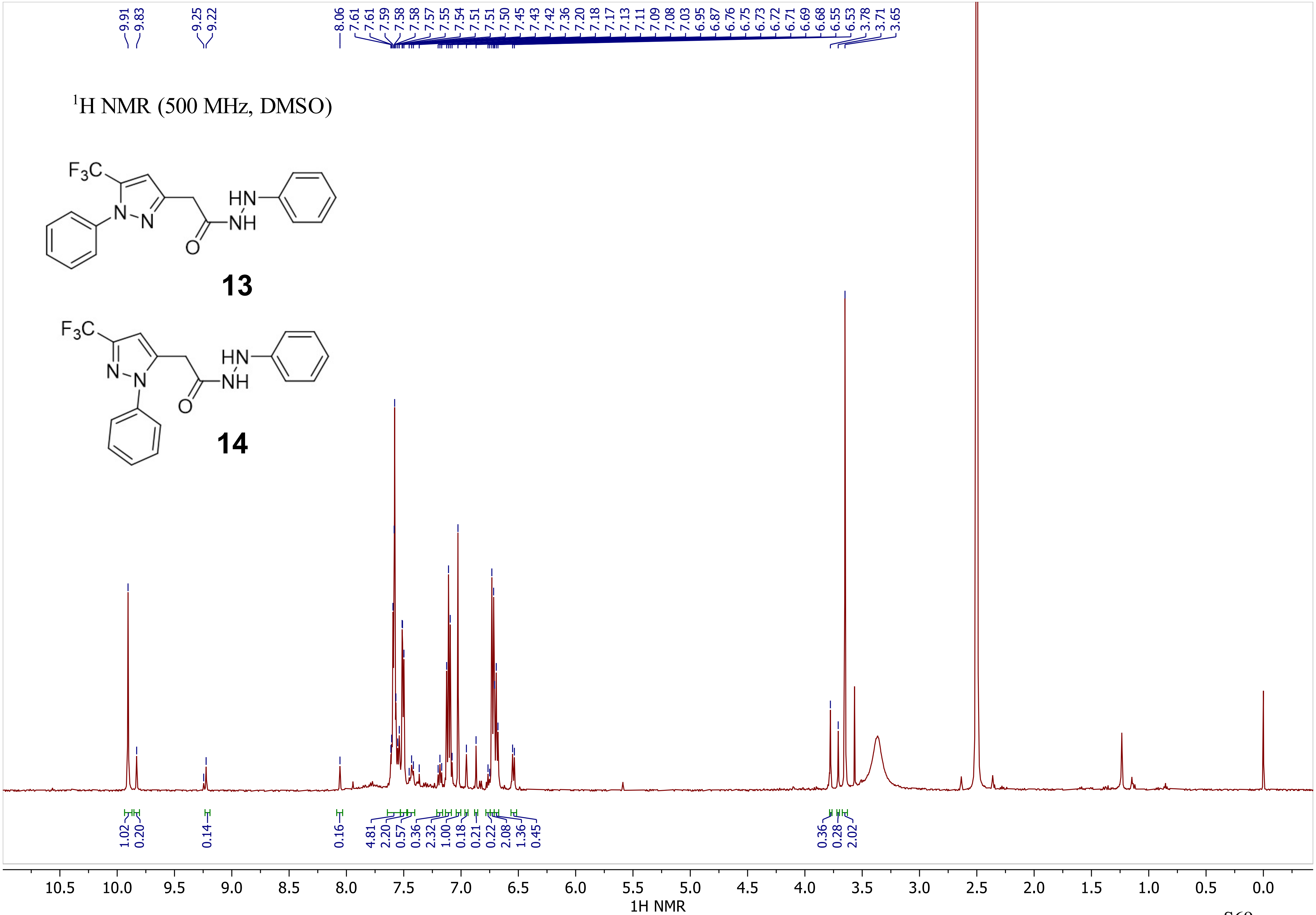

^{1}H NMR (500 MHz, DMSO)
13
14
9.91 9.83 9.25 9.22 8.06 7.61 7.61 7.59 7.58 7.58 7.57 7.55 7.54 7.51 7.51 7.50 7.45 7.43 7.42 7.36 7.20 7.18 7.17 7.13 7.11 7.09 7.08 7.03 6.95 6.87 6.76 6.75 6.73 6.72 6.71 6.69 6.68 6.55 6.53 3.78 3.71 3.65
1.02 0.20 0.14 0.16 4.81 2.20 0.57 0.36 2.32 1.00 0.18 0.21 0.22 2.08 1.36 0.45 0.36 0.28 2.02
1H NMR

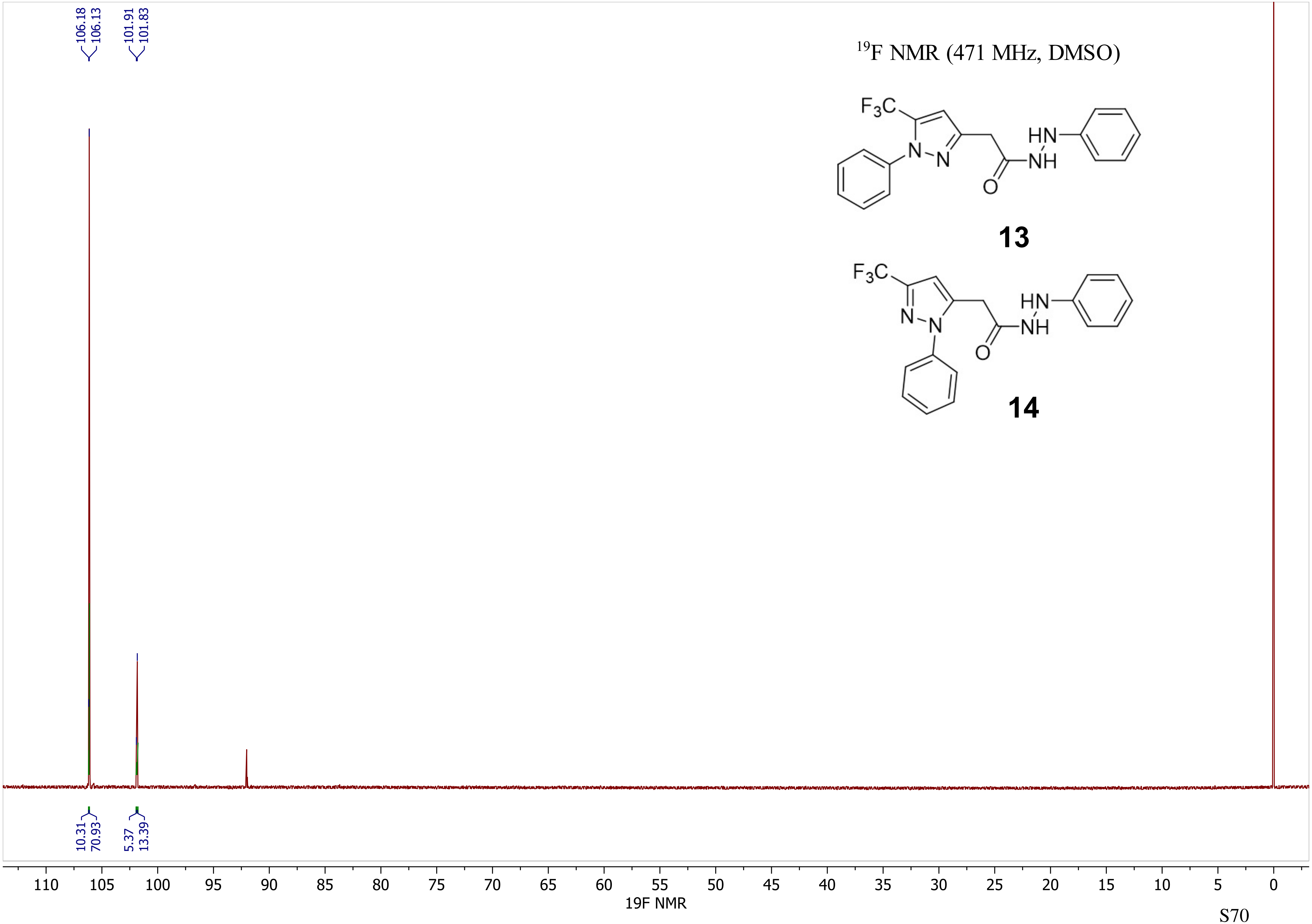

106.18
106.13
101.91
101.83
^{19}F NMR (471 MHz, DMSO)
F_3C
HN
NH
O
N
13
14
10.31
70.93
5.37
13.39
110
105
100
95
90
85
80
75
70
65
60
55
50
45
40
35
30
25
20
15
10
5
0
19F NMR
